# Isoxazole-based molecules restore NK cell immune surveillance in hepatocarcinogenesis by targeting TM4SF5 and SLAMF7 linkage

**DOI:** 10.1038/s41392-024-02106-6

**Published:** 2025-01-20

**Authors:** Ji Eon Kim, Hyun Su Kim, Wonsik Kim, Eun Hae Lee, Soyeon Kim, Taewoo Kim, Eun-Ae Shin, Kyung-hee Pyo, Haesong Lee, Seo Hee Jin, Jae-Ho Lee, Soo-Min Byeon, Dong Joo Kim, Jinwook Jeong, Jeongwon Lee, Minjae Ohn, Hyojung Lee, Su Jong Yu, Dongyun Shin, Semi Kim, Jun Yeob Yoo, Seung-Chul Lee, Young-Ger Suh, Jung Weon Lee

**Affiliations:** 1 Department of Pharmacy, College of Pharmacy, Seoul National University, Seoul, Republic of Korea; 2 Research Institute of Pharmaceutical Sciences, College of Pharmacy, Seoul National University, Seoul, Republic of Korea; 3 College of Pharmacy and Institute of Pharmaceutical Sciences, CHA University, Pocheon-si, Gyeonggi-do Republic of Korea; 4 Department of Internal Medicine and Liver Research Institute, Seoul National University College of Medicine, Seoul, Republic of Korea; 5 College of Pharmacy, Gachon University, Incheon, Republic of Korea; 6 Microbiome Convergence Research Center, Korea Research Institute of Bioscience and Biotechnology, Daejeon, Republic of Korea; 7 CHA Advanced Research Institute, Seongnam-si, Gyeonggi-do Republic of Korea

**Keywords:** Drug development, Target validation, Gastrointestinal cancer, Immunotherapy

## Abstract

Dynamic communication between hepatocytes and the environment is critical in hepatocellular carcinoma (HCC) development. Clinical immunotherapy against HCC is currently unsatisfactory and needs more systemic considerations, including the identification of new biomarkers and immune checkpoints. Transmembrane 4 L six family member 5 (TM4SF5) is known to promote HCC, but it remains unclear how cancerous hepatocytes avoid immune surveillance and whether avoidance can be blocked. We investigated how TM4SF5-mediated hepatic tumorigenesis avoids surveillance by natural killer (NK) cells, which are prevalent in the liver, and whether the avoidance can be blocked by anti-TM4SF5 agents. We used comprehensive structure activity relationship analysis to identify TM4SF5-specific isoxazole (TSI)-based small molecules that inhibit TM4SF5-mediated effects. TM4SF5 expressed by hepatocytes reduced NK cell cytotoxicity by downregulating stimulatory ligands/receptors, including signaling lymphocytic activation molecule family member 7 (SLAMF7). TM4SF5 bound SLAMF7 depending on *N*-glycosylation and caused intracellular trafficking of SLAMF7 from the plasma membrane to lysosomes for degradation. TSI treatments in cell lines and animal models of HCC blocked this binding, intracellular trafficking, and downregulation, resulting in higher levels of stimulatory NK cell ligands. In mouse xenograft models, TSI treatment abrogated HCC development by increasing the abundance and dispersion of Slamf7-positive cells in liver tissues, recapitulating the phenotype of *Tm4sf5*-knockout mice and indicating TSI-mediated restoration of NK cell surveillance. These findings suggest that TSIs can inhibit TM4SF5-mediated liver carcinogenesis by increasing NK cell surveillance.

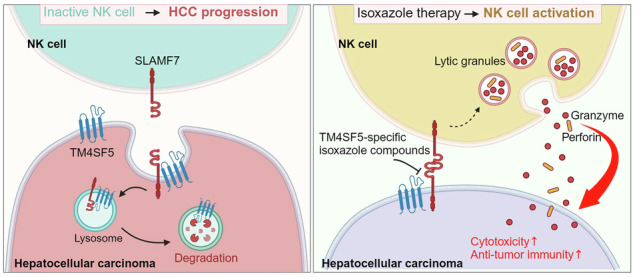

## Introduction

Liver injury causes chronic liver diseases, including metabolic dysfunction-associated steatotic liver disease (MASLD), fibrosis and cirrhosis, and eventually hepatocellular carcinoma (HCC).^1^ The inflammatory and fibrotic environment activate abnormal signaling for cell proliferation, leading to HCC.^2,3^

Although the liver is central for metabolism of nutrients and clearance of toxins via extensive vascular networks, it is also specialized for maximal immune surveillance. NK cells making up ~50% of the liver lymphocyte population,^4^ kill cancer cells by releasing cytotoxic granules^5^ and interferon-γ (IFN-γ),^6,7^ as well as by expressing cell death ligands.^8^ Unlike T cells that rely on antigen presentation,^9^ NK cells are activated via a cascade of inactivation and activation receptors on the surface. Their cytotoxicity depends on “missing-self” or “induced-self” recognition and a precise counterbalance among co-inhibitory and co-stimulatory signaling via functional receptors, eventually resulting in the inactivation or activation of NK cell cytotoxicity.^10^ NK cell-mediated killing of tumor cells can be suppressed by the activation of immune checkpoints, such as cytotoxic T lymphocyte-associated antigen 4 (CTLA-4) and programmed cell death protein 1 (PD-1), on NK cell surfaces.^1,11^ Recently, antibodies against programmed cell death ligand 1 (PD-L1)/PD-1 and CTLA-4 checkpoints have been used therapeutically in patients with cancer.^12,13^ Vascular endothelial growth factor (VEGF) inhibition is tested for synergistic effects with immunotherapy in patients with unresectable HCC in phase III clinical trials.^14^ However, upper gastrointestinal bleeding was observed as a life-threatening complication in patients with cirrhosis and HCC.^15^ Furthermore, metabolic dysfunction-associated steatohepatitis (MASH) is a rapidly rising cause of HCC, and patients with MASH-related HCC at an advanced stage have limited potential for curative strategies.^16^ Anti-PD-1 treatment triggered an increase in HCC despite increases in CD8^+^ and TNF^+^ T cells; CD8^+^ T cells can help induce MASH-related HCC rather than executing immune surveillance.^17^ Therefore, new biomarkers for the transition from MASH to HCC can be identified and targeted.^18^ Although the complex activation/inactivation pathways of NK cells are well understood,^7^ further investigations of HCC-specific checkpoints in hepatocytes and NK cells are warranted to develop better curative options.

Transmembrane 4L six family member 5 (TM4SF5)^19^ is an *N-*glycosylated membrane protein with four transmembrane domains,^20^ highly expressed in human liver cancer tissues,^21,22^ and induced by proinflammatory cytokines and chemokines, including Ccl2, Ccl5,^23^ and transforming growth factor β (Tgfβ1) in carbon tetrachloride (CCl_4_)-mediated liver fibrosis in mice.^24^ Hepatic stellate cells, endothelial cells, and Kupffer cells also express TM4SF5, via induction in inflammatory environments.^25^ TM4SF5 activates focal adhesion kinase (FAK),^26^ proto-oncogene tyrosine-protein kinase Src (c-SRC),^27^ and signal transducer and activator of transcription 3 (STAT3).^28^ Although diethylnitrosamine (DEN)-induced and Tm4sf5-mediated liver carcinogenesis involves Cd4^+^ T and NK cell populations,^29^ the mechanism by which Tm4sf5 causes downregulation of stimulatory NK cell ligands, and whether any Tm4sf5-inhibitory modality can abrogate such effects, remains largely unexplored.

We explored the mechanistic roles of hepatocyte TM4SF5 in reducing NK cell cytotoxicity during liver carcinogenesis. We found that TM4SF5 expressed in hepatocytes or NK92 cells reduced NK cell cytotoxicity in vitro and in animal HCC models by binding to the stimulatory NK cell ligand and receptor SLAMF7, resulting in intracellular trafficking of SLAMF7 toward lysosomes for degradation. TM4SF5 might thus be an NK cell immune checkpoint, and that treatment of TM4SF5-specific isoxazole-based small molecules (TSIs) recovered NK cell cytotoxicity, leading to abrogated HCC. These results suggest that TSIs hold promise as a new class of NK cell immune checkpoint inhibitors.

## Results

### TM4SF5-mediated NK cell inactivation promotes hepatocellular carcinoma

**Fig. 1:**
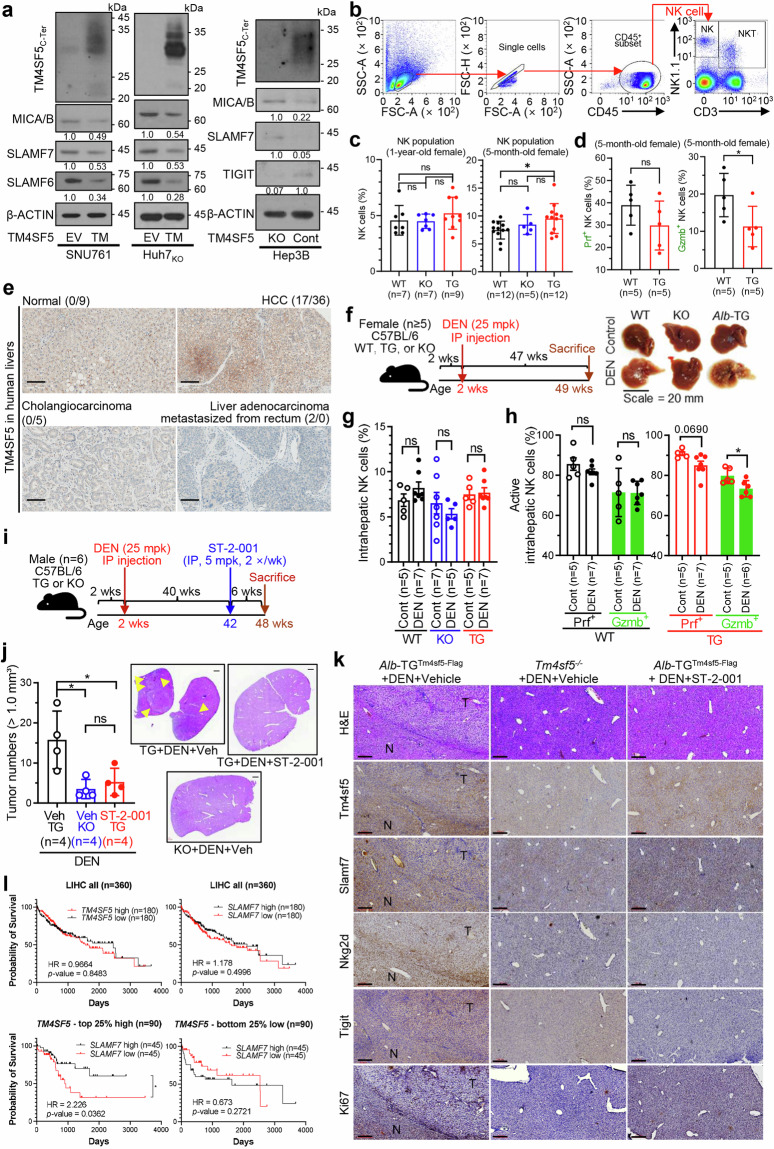
TM4SF5-mediated NK cell inactivation promotes hepatocellular carcinoma. **a** TM4SF5-negative SNU761 cells or Huh7_KO_ (*TM4SF5*-knockout) cells with stable expression of empty vector (EV) or TM4SF5 (TM) were subjected to immunoblotting for the indicated molecules. **b** Immunostaining of Cd45^+^Cd3^*−*^Nk1.1^+^ NK cells from liver tissues of C57BL/6 female mice at 5 months or 1 year of age via flow cytometry. **c**, **d** In addition to population analysis (**c**), NK cells were immunostained for perforin (Prf^+^) or granzyme (Gzmb^+^) (**d**). *P < 0.05, **P < 0.01, ***P < 0.001, ****P < 0.0001, ns = non-significant, unpaired Student’s *t* test or ordinary one-way ANOVA. Data are represented as the mean ± SEM. **e** Tissue microarrays were processed using anti-human TM4SF5 polyclonal antibody.^34^ **f** Scheme for liver cancer models with DEN administration in female WT, *Alb*-TG^Tm4sf5-Flag^ (TG), or *Tm4sf5*^*−*/*−*^ KO C57BL/6 mice (n ≥ 5). **g**, **h** Liver tissues were analyzed for intrahepatic NK cell populations and perforin (Prf) and granzyme (Gzmb) immunostaining. For Gzmb^+^ NK cell measurement of DEN-treated TG mice^,^ one sample was lost by a spill, resulting in six sample size. **i** Scheme for liver cancer models of DEN administration in the presence of vehicle or ST-2-001 treatment in WT, *Alb*-TG^Tm4sf5-Flag^ (TG), or *Tm4sf5*^*−*/*−*^ KO C57BL/6 mice (*n* = 6). **j** Tumor numbers larger than 1 mm^3^ in the livers were counted, and graphed following eliminations of the highest and smallest values in each group (left, **j**). Representative H&E images of whole liver tissues from each experimental condition (right, **j**). **k** Liver tissues were processed for immunohistochemistry for the indicated molecules. Scale bars, 100 μm. *P < 0.05, **P < 0.01, ***P < 0.001, ****P < 0.0001, ns = non-significant, unpaired Student’s *t* test or Two-way ANOVA, Tukey’s multiple comparisons test. Data represents the mean ± SEM. **l** Among LIHC dataset (*n* = 360, LIHC all) or top 25% high group of *TM4SF5* expression (*n* = 90) from TCGA, overall survival probability depending on *SLAMF7* expression levels (*SLAMF7*-high, top 50% of *SLAMF7* expression (*n* = 180 or 45, respectively); *SLAMF7*-low, bottom 50% of *SLAMF7* expression (*n* = 180 or 45, respectively) were analyzed for hazard ratio (HR) and *p* values. Data represents three independent experiments. See Figs. S1, S2, and S3

TM4SF5 is shown to be involved in HCC development^21^ via inactivation of NK cells,^29^ although it is still unclear how TM4SF5 affects NK cell activity. Since there are diverse stimulatory or inhibitory ligands expressed in hepatocytes, we explored how TM4SF5 could specifically influence NK cells. In our efforts to understand the effects of human hepatocyte TM4SF5 expression on NK cell activity, we found that TM4SF5 overexpression in TM4SF5-null SNU761 cells or TM4SF5-knockout Huh7_KO_ cells resulted in TM4SF5-dependent decreases in MICA/B, SLAMF6, and SLAMF7 expression, whereas SLAMF7 and MICA/B increased upon knockout of endogenous TM4SF5 in Hep3B cells, but TIGIT decreased (Fig. 1a). When liver tissues from 5-month-old or 1-year-old female C57BL/6 mice were further analyzed for Cd45^+^Cd3^*−*^NK1.1^+^ cells (Fig. 1b, Supplementary Gating Strategy GS1), NK cell population in the TG mice at 5-month-old age was significantly or insignificantly higher than those in WT or KO mice, respectively, although animals at 1-year-old age were non-differential (Fig. 1c); however, 5-month-old TG mice showed lower NK cell activity with significantly lower granzyme expression compared with WT mice (Fig. 1d). Thus, the lower NK cell population but higher active NK cell population in WT mice compared with TG mice suggests that TM4SF5 expression may affect NK cell activity more than population size. Furthermore, when partial liver tissues from the 5-month-old mice were analyzed by immunoblot, KO mice showed higher perforin and Slamf7 or granzyme expression compared with those of TG or WT mice, respectively, although the level variance between each animal was observed. Meanwhile, there were no differences in the expression levels of Nkg2d, Slamf6, Tigit, and Cd155 among the animal groups (Supplementary Fig. S1a). Thus, TM4SF5 expression in hepatocytes may be linked to downregulation of NK cell activity.

NK cells can account for up to 50% of total intrahepatic lymphocytes.^4^ Tissue microarray experiments using anti-TM4SF5 antibody showed that more than half of HCC tissues from patients had TM4SF5-positive immunostaining, whereas their normal liver tissue counterparts, cholangiocarcinoma tissues, and liver adenocarcinoma tissues metastasized from the rectum were TM4SF5-negative, suggesting that TM4SF5 is relevant in HCC but not in other types of liver cancer (Fig. 1e). Furthermore, DEG analysis of RNA-Seq datasets from the livers of 3-month-old male WT or *Tm4sf5*^*−*/*−*^ C57BL/6 mice (*n* = 4) showed clear differences in the expression of signaling pathways related to NK cells and HCC (Supplementary Fig. S1b and S1c). These observations suggest that hepatic Tm4sf5 expression may be involved in NK cell inactivation, leading to HCC development.

We next analyzed NK cells from liver tissues of C57BL/6 mice (*n* = 3) treated with DEN with or without additional CCl_4_ treatment for 26 weeks, prior to sacrifices at 38 weeks of age (Supplementary Fig. S2a). When WT or *Tm4sf5*^*−*/*−*^ (KO) C57BL/6 mice were administered with DEN+CCl_4_, liver weights and spleen sizes were not different between animal groups; but tumor volume and nodule diameter (>1.0 mm) were greater in WT mice than in KO mice under DEN+CCl_4_ treatment, although the treatment did not significantly change their levels in KO mice (Supplementary Fig. S2b and S2c). Furthermore, *Tm4sf5* knockout-mediated tumor surveillance appeared to involve increased Prf and Slamf7 expression in splenic NK cells, although the trend of increasing Slamf7 expression was not statistically significant (*p* = 0.0760; Supplementary Fig. S2d, Supplementary Gating Strategy GS2). However, the splenic or intrahepatic NK cell populations and intrahepatic Slamf7^+^ NK cells were not different between the animal groups (Supplementary Fig. S2d). When WT, KO, or TG C57BL/6 female mice (*n* ≥ 5) were administered with DEN alone for 47 weeks (Fig. 1f), there were no changes in intrahepatic NK cell numbers among the groups (Fig. 1g, Supplementary Gating Strategy GS1), whereas the TG mice had marginally lower numbers of Prf^+^ NK cells (*p* = 0.0690) and significantly fewer Gzmb^+^ NK cells than the WT mice (Fig. 1h, Supplementary Gating Strategies GS3, 4). Such effects of TM4SF5 expression might be limited to certain levels, presumably due to possible random HCC development of female C57BL/6 mice following a long-term DEN administration (Fig. 1g, h).

We next examined whether treatment with ST-2-001 could abrogate TM4SF5-mediated tumor development in DEN-treated TG mice. ST-2-001 (5 mpk) was intraperitoneally injected into mice twice per week for the last 6 weeks of the 46-week DEN treatment (Fig. 1i). Liver tissue analysis revealed obvious tumor development in TG mice treated with DEN alone, unlike KO mice, whereas there was no obvious tumor formation in TG mice treated with DEN and ST-2-001 (Fig. 1j). Analysis of mRNAs from the liver tissues showed that *Slamf7*, *Nkg2d*, *Crtam*, and *Dnam1* mRNA levels were lower in DEN-administered TG mice than in DEN-administered *Tm4sf5*^*−*/*−*^ mice, and that ST-2-001 treatment tended to restore these levels, albeit not to a statistically significant degree (Supplementary Fig. S2e). Experiments with in-vitro hepatocyte systems also showed TM4SF5-dependent modulation of inhibitory or stimulatory NK cell ligands, which was reversed by ST-2-001 treatment (Supplementary Fig. S2f and S2g). Furthermore, the tumor lesions from DEN-treated TG mice showed weaker Slamf7 and Nkg2d immunostaining and stronger Tigit and nuclear Ki67 staining compared with non-tumor areas, although TM4SF5 staining was comparable between tumor and non-tumor regions (Fig. 1k). This result may be due to TM4SF5’s involvement not only in HCC but also in pre-cancerous liver features, including MASH-associated fibrosis.^23,25^ These cancerous effects were not seen in DEN-treated *Tm4sf5*^*−*/*−*^ mice or in TG mice treated with DEN and ST-2-001 (Fig. 1k). Thus, the anti-TM4SF5 isoxazole ST-2-001 appeared to block the TM4SF5-mediated suppression of stimulatory NK cell ligands, including Slamf7. Analysis of LIHC data from TCGA further showed the functional relationship between TM4SF5 and SLAMF7 in liver cancer. Among patients with LIHC, higher *TM4SF5* expression was linked to lower *SLAMF7* expression, resulting in poor survival rates; patients with TM4SF5^high^ SLAMF7^low^ tumors showed poorer survival compared with patients with TM4SF5^high^ SLAMF7^high^ tumors (Fig. 1l). However, other NK cell ligands had not appeared significantly linked to the TM4SF5^high^ phenotype for survival probability (Supplementary Fig. S3). These results from in vitro cells, in vivo mice, and clinical patient datasets suggest that hepatocyte TM4SF5 promotes HCC development by downregulating SLAMF7, leading to NK cell inactivation, which can be blocked by anti-TM4SF5 isoxazoles.

### Isoxazoles abrogate HCC development by inhibiting TM4SF5-dependent signaling

**Fig. 2:**
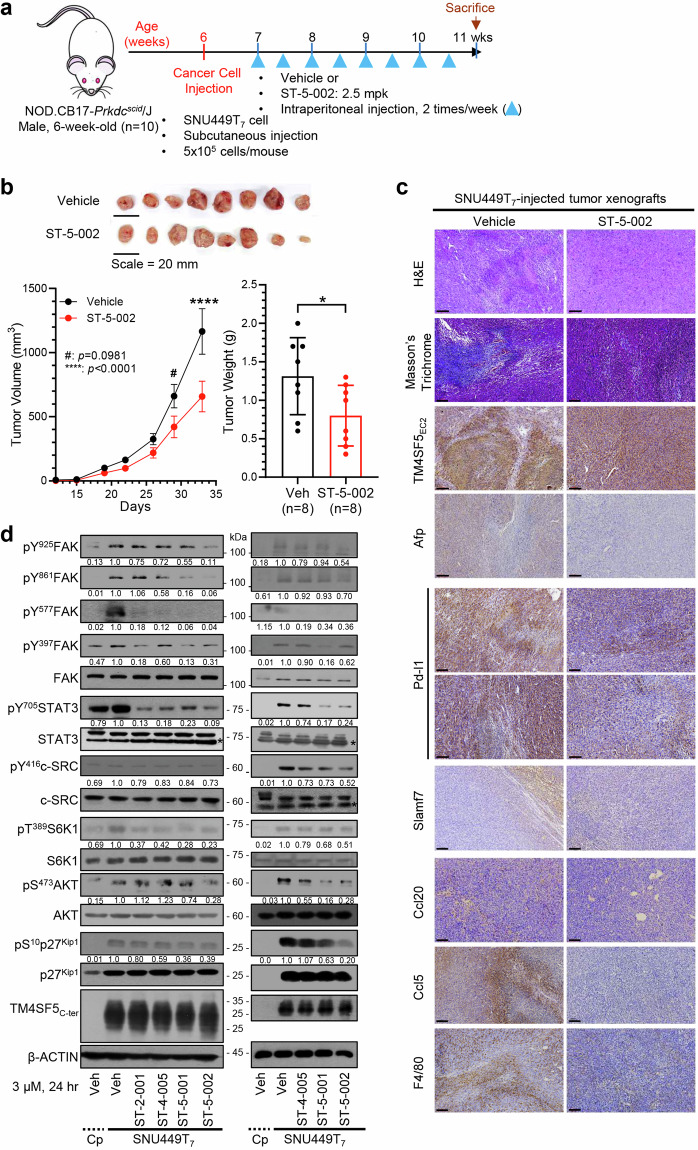
Isoxazoles abrogated HCC development via inhibitions of signal activity depending on TM4SF5 expression. **a** Scheme for TM4SF5-expressing SNU449T_7_ cell xenograft models with NOD-SCID male mice (6-week-old, *n* = 10). **b** Tumor volumes were measured twice a week with caliper and calculated. Tumor weight was measured right after sacrifice and tumor extraction. The maximal and minimal tumor weight values were eliminated as outliers for animal variances upon drug treatment, resulting in data values at *n* = 8 for graphs and tumor images. **c** Liver tissues were processed to immunostaining for the indicated molecules. Representative images were shown. Scale bars, 100 μm. **d** Subconfluent TM4SF5-negative SNU449Cp (Cp) or TM4SF5-positive SNU449T_7_ cells were treated with vehicle or isoxazoles (3 μM) for 24 h, prior to harvests and immunoblots. * in the immunoblots indicate non-specific bands. *P < 0.05, unpaired Student’s *t* tests or Two-way ANOVA. Data are represented as mean ± SEM. Data stands for three independent experiments. See also Fig. S4

Although we found the specific linkage between hepatocyte TM4SF5 expression and SLAMF7 downregulation on NK cells, we further wondered whether TM4SF5-specific inhibition might lead to inhibiting of TM4SF5-mediated tumor formation in immune-deficient mice via downregulations of TM4SF5-mediated signaling activities. We used structure-activity relationship analysis to identify more advanced isoxazoles based on ST-2-001, focusing on systematic structural modification of substituents in the 3,5-bisaryl isoxazole core. We then established an in vivo model using intraperitoneal injection of vehicle or ST-5-002 in male NOD-SCID mice (*n* = 10) that were subcutaneously inoculated with SNU449T_7_ cells (Fig. 2a). At 33 days after inoculation with SNU449T_7_ cells, the tumor volumes of mice treated with ST-5-002 were significantly lower than those of mice treated with vehicle (Fig. 2b). Furthermore, histological analysis showed more malignancy in the vehicle-treated xenografts compared with the ST-5-002–treated xenografts. The vehicle-treated xenografts showed clear expression of TM4SF5 and Afp, which are highly expressed in HCC, as well as inflammatory molecules, and the livers in these mice were more disorganized, fibrotic, and damaged compared with those in ST-5-002–treated mice. The vehicle-treated xenografts also showed higher Pd-l1 expression and more restricted Slamf7 expression in non-tumor regions compared with the ST-5-002–treated xenografts (Fig. 2c). In vitro systems using SNU449Cp cells or SNU449T_7_ cells were further analyzed for the effects of TSIs on TM4SF5-mediated intracellular signaling. Phosphorylation of FAK, c-SRC, STAT3, p70 ribosomal S6 kinase (S6K1), AKT serine/threonine kinase 1, and p27^Kip1^ was higher in SNU449T_7_ cells than in SNU449Cp cells and was mostly abolished by ST-2-001, ST-4-005, ST-5-001, or ST-5-002 treatment (Fig. 2d). Although ST-5-001 and ST-5-002 treatment of TM4SF5-positive hepatocytes decreased intracellular signaling activities, it did not alter the signaling activities of TM4SF5-negative cells, showing specific TSI inhibitory effects against TM4SF5-mediated signaling activity (Supplementary Fig. S4a). In addition, these anti-TM4SF5 isoxazoles showed inhibitory effects on TM4SF5 binding to membrane proteins and receptors involved in chronic liver malignancy^25,30–34^ and inhibited TM4SF5-dependent sphere growth in aqueous 3D culture conditions (Supplementary Fig. S4b and S4c). These results suggest that TSIs inhibit the signaling activities mediated by hepatocyte TM4SF5 during tumor development.^21,22,35,36^

### TM4SF5-mediated downregulation of SLAMF7 promotes HCC development

**Fig. 3:**
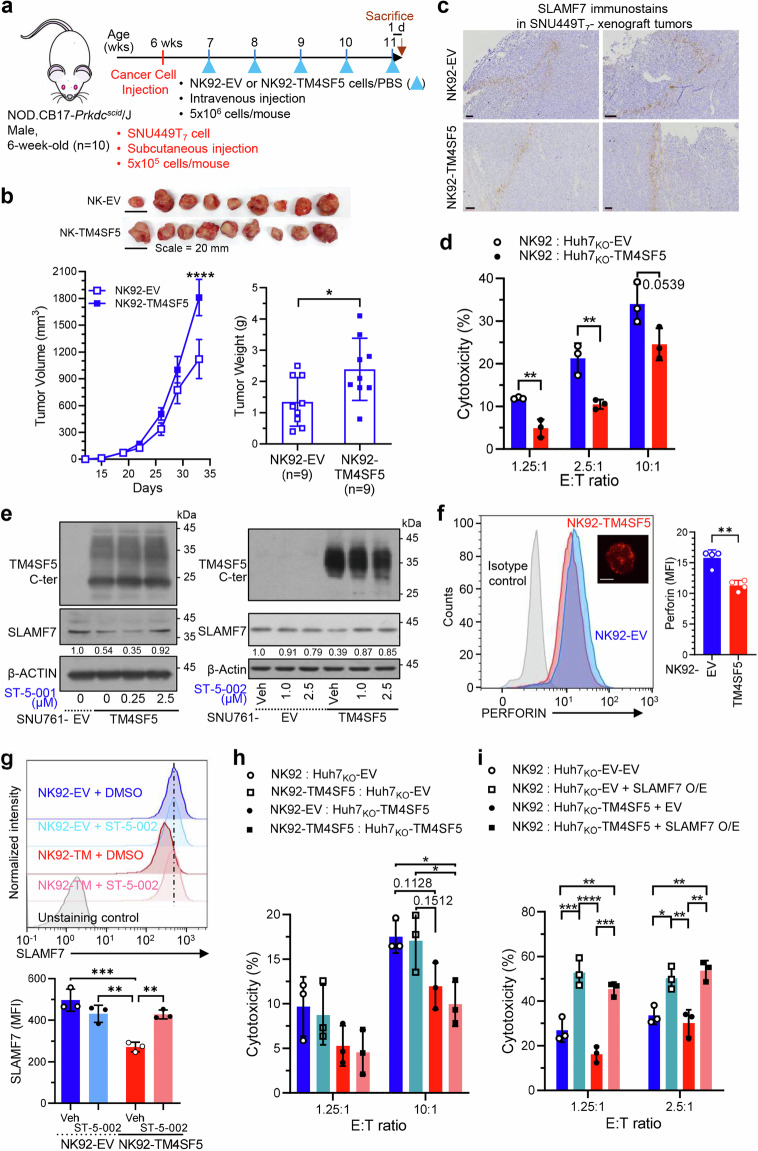
TM4SF5-mediated downregulation of SLAMF7 leads to hepatocarcinoma development. **a** Scheme for NK cell adoptive transfer models in male NOD-SCID mice (6-week-old, *n* = 10). **b** Tumor size was measured twice a week with calipers. Tumor weight was measured after sacrifice and tumor extraction. *P < 0.05, unpaired Student’s *t* test or Two-way ANOVA. Data are represented as the mean ± SEM. **c** Tumor tissues were immunostained with Slamf7 antibody. **d** Analysis of NK92 cell cytotoxicity toward Huh7_KO_-EV or Huh7_KO_-TM4SF5 cells. **e** Immunoblots of SNU761-EV or SNU761-TM4SF5 cells treated with vehicle (0) or ST-5-001 at the indicated concentrations for 24 h. **f**–**i** Flow cytometry analysis of perforin (**f**) and SLAMF7 (**g**) expression in NK92-EV and NK92-TM4SF5 cells without (**f**) or with (**g**) vehicle DMSO or ST-5-002 treatment. MFI of perforin or SLAMF7 were graphed. The representative fluorescent image of TM4SF5 (red) in NK92-TM4SF5 cells was inserted (**f**). Analyses of NK92-EV and NK92-TM4SF5 cell cytotoxicity toward Huh7_KO_-EV and Huh7_KO_-TM4SF5 cells without (**h**) or with (**i**) overexpression (O/E) of human SLAMF7 in the hepatocytes. *P < 0.05, **P < 0.01, ***P < 0.001, ***P < 0.0001, ns non-significant, unpaired Student’s *t* test or Two-way ANOVA. Data are represented as the mean ± SEM. Data represents three isolated experiments

Given that hepatocyte TM4SF5 expression is specifically linked to SLAMF7 downregulation, we further explored whether TM4SF5 expression in NK cells might also lead to NK cell inactivation for tumor development, and how TSI treatment might recover the TM4SF5-mediated SLAMF7 downregulation. Because hepatocyte TM4SF5 influenced the levels of stimulatory NK cell ligands during HCC development in the xenograft models, we further examined TM4SF5-mediated effects on NK cells. Since cross-talks between cancerous hepatocytes and NK cells may indirectly or directly (via trogocytosis) affect TM4SF5 expression and location on NK cells, we first engineered NK92 cells expressing empty vector or ectopic TM4SF5-HA via lentiviral infection. Then, starting 1 week after SNU449T_7_ cells were subcutaneously xenografted into male NOD-SCID mice (*n* = 10), we intravenously injected NK92-EV or NK92-TM4SF5 cells into the mice every week for 5 weeks. Tissues were harvested and analyzed 1 day after the last NK92 cell injection (Fig. 3a). We found that compared with NK92-EV cells, NK92-TM4SF5 cells caused more aggressive tumor formation (Fig. 3b), although Slamf7 immunostaining in the xenografted tumors was not clearly different between the two NK92 cell lines (Fig. 3c). The SLAMF7-positive staining was unexpectedly in strip-like and somewhat patched patterns, presumably due to more standing-out signals from the injected NK cells not enough for wider infiltrations shortly (1 day) before the analysis.

We then asked whether TM4SF5 expression could affect NK cell cytotoxicity. In-vitro NK92 cell cytotoxicity against TM4SF5-negative Huh7_KO_-EV cells was greater than that against TM4SF5-positive Huh7_KO_-TM4SF5 cells (Fig. 3d). TM4SF5 expression in hepatocytes reduced SLAMF7 protein expression, and this effect was reversed by ST-5-001 or ST-5-002 treatment (Fig. 3e). Perforin was slightly expressed in most NK92 cells upon stable TM4SF5 expression (i.e., NK92-TM4SF5 cells) but with decreased median fluorescence intensity, compared with that of control NK92-EV cells (Fig. 3f). When NK92-EV and NK92-TM4SF5 cells with or without ST-5-002 treatment were analyzed for SLAMF7 expression, the NK92-TM4SF5 cells showed lower SLAMF7 expression than the NK92-EV cells in the absence of ST-5-002 treatment, although ST-5-002 treatment of NK92-TM4SF5 cells resulted in a recovery of SLAMF7 expression compared with DMSO treatment (Fig. 3g). These results suggest that TM4SF5 inhibition can modulate NK cell activity. Analyses of the in-vitro cytotoxicity of NK92-EV and NK92-TM4SF5 cells against TM4SF5-negative or TM4SF5-positive hepatocytes revealed TM4SF5-dependent inactivation of NK92 cytotoxicity, wherein TM4SF5 expression in both the effector cells and the target cells resulted in lower cytotoxic activity (Fig. 3h). A further analysis of NK92 cell cytotoxicity against hepatocytes with or without SLAMF7 overexpression showed that SLAMF7 overexpression reversed the TM4SF5-mediated reduction of cytotoxicity, independently of TM4SF5 expression (Fig. 3i). These observations support the hypothesis that TM4SF5 expressions in hepatocytes or NK cells cause SLAMF7 downregulation, leading to NK cell inactivation. Therefore, it is likely that treatment with TSIs can recover TM4SF5-mediated NK cell inactivation via modulating SLAMF7 levels.

### Binding of TM4SF5 to SLAMF7 depending on *N*-glycosylation causes lysosomal degradation of SLAMF7

**Fig. 4:**
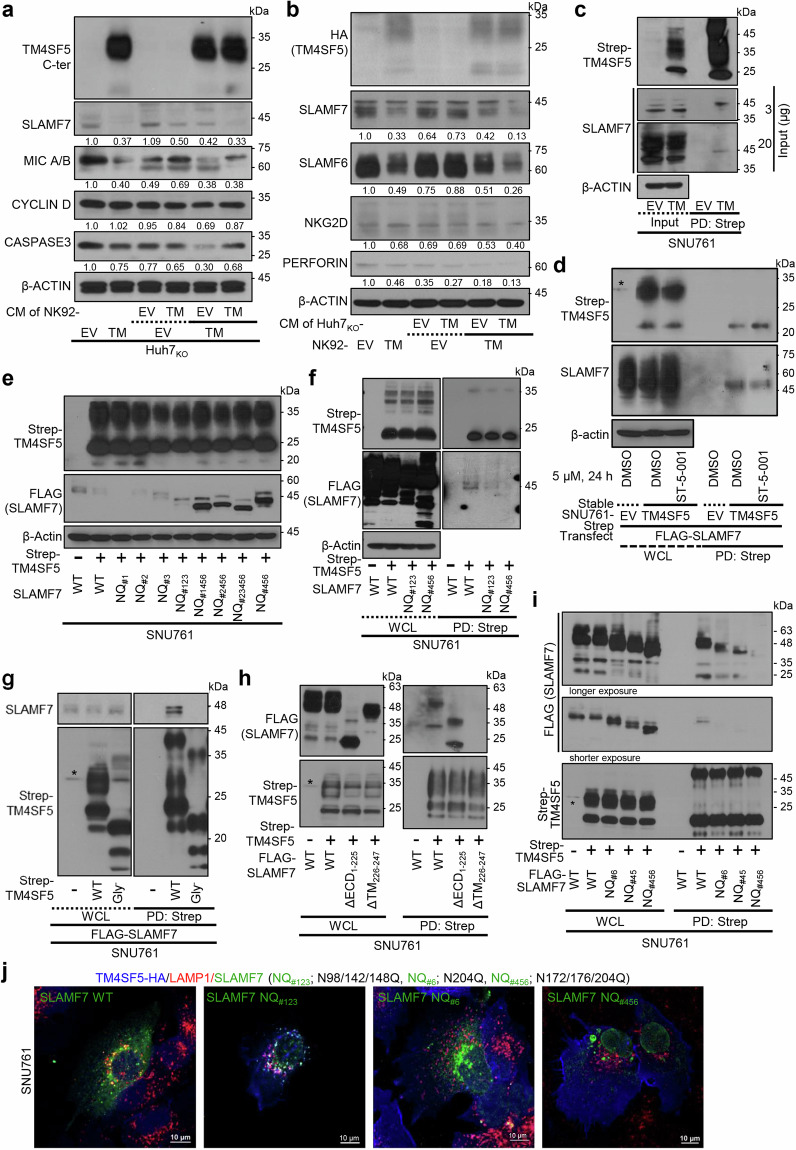
Binding of TM4SF5 to SLAMF7 depending on *N*-glycosylation causes lysosomal degradation of SLAMF7. **a**, **b** Huh7_KO_-HA-EV or Huh7_KO_-HA-TM4SF5 cells were treated with conditioned media (CM) of NK92-EV or NK92-TM4SF5 cells (**a**) or vice versa (**b**) for 24 h before whole-cell lysates were prepared and immunoblotted for the indicated molecules. **c**, **d** Subconfluent SNU761 cells expressing Strep empty vector (Strep-EV) or Strep-TM4SF5 (TM) were treated without (**c**) or with (**d**) DMSO or ST-5-001 were harvested. Cell lysates were pulled down using streptavidin-agarose beads and immunoblotted for SLAMF7. Different amounts of input proteins were loaded for SLAMF7 immunoblotting to see clear coimmunoprecipitation of endogenous SLAMF7 (**c**). **e**, **f** SNU761 cells were transiently transfected with WT or various SLAMF7 mutants of *N*-glycosylation residues (N98, N142, N148, N172, N176, and/or N204), prior to immunoblots for the indicated molecules (**e**) or for coimmunoprecipitation (**f**). **g** SNU761 cells transfected with FLAG-SLAMF7 and Strep-TM4SF5 WT or Gly^–^ were harvested and lysed with Brij58-containing lysis buffer. Whole-cell lysates were subjected to streptavidin pulldown and immunoblot. **h**, **i** SNU761 cells transfected with Strep-TM4SF5 and FLAG-SLAMF7 deletion mutants (**h**) or *N-*glycosylation mutants (**i**) were harvested and lysed with Brij58-containing lysis buffer. Whole-cell lysates were subjected to streptavidin pulldown and immunoblot. ΔECD: Δ1–225; ΔTM: Δ226–247; NQ_#6_:N204Q; NQ_#45_:N172/176Q; NQ_#456_:N172/176/204Q. * in the immunoblots indicate non-specific bands. **j** SNU761 cells were transfected with HA-TM4SF5 together with FLAG-SLAMF7 WT, NQ_#6_, or NQ_#456_. SLAMF7 with *N*-glycosylation mutations; NQ_#1_; N98Q, NQ_#2_; N142Q, NQ_#3_; N148Q, NQ_#123_; N98/142/148Q, NQ_#456_; N1172/176/204Q, NQ_#1456_; N98/172/176/204Q, NQ_#2456_; N142/172/176/204Q, NQ_#23456_; N142/148/172/176/204Q. Cells were randomly stained with HA (blue), FLAG (green), and LAMP1 (red). Images were randomly captured by a confocal microscope. Data represent three independent experiments. See also Fig. S5

We next investigated the mechanism of TM4SF5-mediated downregulation of stimulatory NK cell ligands and receptors, especially with regard to SLAMF7 expression levels and intracellular traffic depending on TM4SF5. First, we used experiments with conditioned media (CM) to determine if indirect interaction between cancerous hepatocytes and NK92 cells leads to modulation of the ligands or receptors. When we treated Huh7_KO_-TM4SF5 or Huh7_KO_-EV cells with CM from cultures of NK92-EV or NK92-TM4SF5 cells, SLAMF7 and MICA/B levels were lower in the CM-treated Huh7_KO_-TM4SF5 cells than in the CM-treated Huh7_KO_-EV cells, and the CM from NK92-TM4SF5 cells had a greater negative effect on SLAMF7 and MICA/B expression than the CM from NK92-EV cells (Fig. 4a). We observed similar effects in reciprocal experiments where NK92 cells were treated with CM from Huh7_KO_ cells; treatment of NK92-TM4SF5 cells with CM from Huh7_KO_-TM4SF5 cells led to lower SLAMF7, SLAMF6, NKG2D, and perforin levels in NK92 cells, indicating inactivation of NK cell cytotoxicity (Fig. 4b). These results are in accordance with our in vivo observations of more aggressive tumor formation in mice injected with NK92-TM4SF5 cells than in mice injected with NK92-EV cells (Fig. 3a, b). They also highlight the possibility of direct effects of immune synapse formation or trogocytosis in TM4SF5-mediated communications between hepatocytes and NK cells, which we will explore in the future.

TM4SF5 was directly bound to different stimulatory NK cell ligands, including endogenous SLAMF7, even when the expression levels of the ligands were reduced (Supplementary Fig. S5 and Fig. 4c). The binding of TM4SF5 to SLAMF7 appeared possible with a certain type of *N*-glycosylation pattern in SLAMF7, upon comparison between input or coimmunoprecipitated SLAMF7 (Fig. 4c). The binding between TM4SF5 and SLAMF7 was abolished by ST-5-001 (Fig. 4d). Compared with WT SLAMF7 and TM4SF5 expression in SNU449 cells, expression of mutant SLAMF7 at *N*-glycosylation residues (e.g., N98, N142, and/or N148 to Q) resulted in un-changed or rather reduced SLAMF7 expression in the presence of TM4SF5 expression, whereas mutants containing the changes of N172/176/204Q showed dramatically increased SLAMF7 levels (Fig. 4e), indicating that the N172/176/204 residues might be important for TM4SF5-mediated SLAMF7 suppression. Further, we found that the mutant NQ_#123_ (N98/142/148Q) was still slightly bound to TM4SF5 (Fig. 4f), supporting the idea that TM4SF5-bound SLAMF7 can traffic to lysosomes for eventual degradation. In SNU761 cells expressing SLAMF7 and either WT or *N*-glycosylation-deficient (Gly^*−*^, N138A/N155Q) TM4SF5, SLAMF7 was bound by WT TM4SF5 but not by Gly^*−*^ TM4SF5, and the SLAMF7 expression level was reduced in the cells expressing WT TM4SF5 but not in those expressing Gly^*−*^ TM4SF5 (Fig. 4g), suggesting that the binding might affect the SLAMF7 protein level. Deletion of the transmembrane domain (amino acids 226 to 247) of SLAMF7 abolished binding to TM4SF5, whereas deletion of the extracellular domain (amino acids 1 to 225) did not (Fig. 4h). Furthermore, mutations of *N*-glycosylation residues in SLAMF7 (NQ_#6_: N204Q; NQ_#45_: N172/176Q; or NQ_#456_: N172/176/204Q) appeared to abolish the binding to TM4SF5 (Fig. 4i). Thus, it appears likely that the 2^nd^ extracellular (longer) loop of TM4SF5, which includes *N*-glycosylation residues, can associate with the extracellular domain of SLAMF7, which also includes *N*-glycosylation residues and might be functionally linked to the transmembrane domain. Immunofluorescence images revealed efficient colocalization of TM4SF5 WT and SLAMF7 WT at LAMP1-positive lysosomes; by contrast, SLAMF7 NQ_#6_ or NQ_#456_ mutants did not colocalize with TM4SF5, although SLAMF7 NQ_#123_ mutant still showed colocalization with TM4SF5 and/or LAMP1 (Fig. 4j, left). Furthermore, the intracellular localization of the NQ_#456_ mutant appeared within circular boundaries not related to organelles, which was presumably a result of liquid–liquid phase separation for the purpose of stress granule formation^37^ with the *N-*glycosylation-deficient mutant at least at N172/176/204 residues unable to bind to TM4SF5 (Fig. 4j).

### TM4SF5-dependent downregulation of SLAMF7 via lysosomal trafficking

**Fig. 5:**
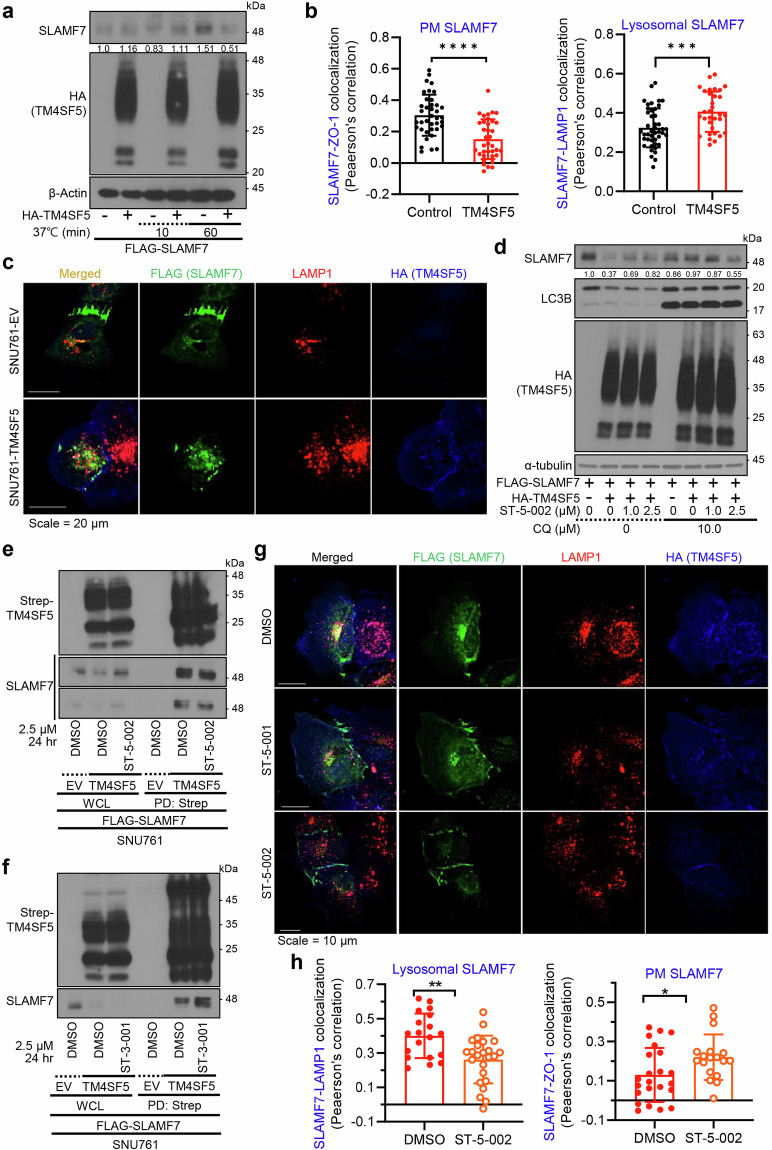
TM4SF5-dependent downregulation of SLAMF7 via lysosomal trafficking. **a** SNU761 cells expressing FLAG-SLAMF7 and HA-EV or HA-TM4SF5 were incubated at 4 °C and then at 37 °C for the indicated times. Cells were harvested and processed for immunoblots using the indicated antibodies. **b**, **c** SNU761 cells were transfected with HA-TM4SF5 and FLAG-SLAMF7 and immunostained with HA, FLAG, and LAMP1 or ZO-1 antibodies. Colocalization coefficients were determined by Pearson’s correlation (**b**). Each dot depicts a correlation value per cell in one of three independent experiments. Representative images are shown (**c**). **d** SNU761 cells were transfected with FLAG-SLAMF7 and HA-EV or HA-TM4SF5 and treated with ST-5-002 with or without chloroquine (CQ, 10 μM) for 24 h. Cells were harvested for immunoblots with the indicated antibodies. Scale bar, 100 μm. **e**–**h** SNU761 cells were transfected with Strep-TM4SF5 and FLAG-SLAMF7 and treated with ST-5-002 (**e**) or ST-3-001 (**f**) for 24 h. Cells were harvested and lysed with Brij58-containing lysis buffer. Whole-cell lysates were subjected to streptavidin pulldown and immunoblots. SNU761 cells were transfected with HA-TM4SF5 and FLAG-SLAMF7 and treated with ST-5-001 or ST-5-002 (2.5 μM, 24 h). Cells were fixed with ice-cold methanol and immunostained with the indicated antibodies. Representative images are shown (**g**). Colocalization coefficients were determined by Pearson’s correlation. Scale bars, 100 μm (**h**). Each dot depicts a correlation value per cell in one of three independent experiments. *P < 0.05, **P < 0.01, ***P < 0.001, ***P < 0.0001, ns non-significant, unpaired Student’s *t* test or Two-way ANOVA. Data is represented as the mean ± SEM. Data represent three isolated experiments. See also Fig. S6

Because we observed colocalization of TM4SF5 and SLAMF7 at lysosomes, we rationalized that TM4SF5 might cause lysosomal degradation of SLAMF7. To measure SLAMF7 internalization, we incubated cells at 4 °C and then returned them to 37 °C for the indicated periods. SNU761-TM4SF5 cells showed lower SLAMF7 expression compared with SNU761-EV cells (Fig. 5a). Furthermore, quantitative analysis of SLAMF7 intracellular translocation showed TM4SF5-dependent localization of SLAMF7 from the plasma membrane to lysosomal membranes (Fig. 5b), where lysosomal degradation might occur (Fig. 5c). Indeed, immunoblots supported such a TM4SF5-dependent decrease in SLAMF7, which was recovered by ST-5-002, and blocking of lysosomal protein degradation with chloroquine prevented TM4SF5-mediated SLAMF7 loss (Fig. 5d). Treatment with ST-5-002 in SNU761 cells expressing Strep-TM4SF5 reduced the TM4SF5 binding to SLAMF7 (a shorter exposure, bottom) concomitantly with a recovery of SLAMF7 from TM4SF5-mediated loss (a longer exposure, upper; Fig. 5e), whereas treatment with the ST-3-001 (Supplementary Fig. S6) neither reduced (but rather increased) the binding nor recovered the TM4SF5-mediated SLAMF7 loss (Fig. 5f). Immunofluorescence showed that treatment with ST-5-001 or ST-5-002 reduced colocalization of TM4SF5 and SLAMF7 at lysosomes, leaving more SLAMF7 at the plasma membrane (Fig. 5g). Quantification of SLAMF7 and LAMP1 colocalization (at lysosomal membranes) or SLAMF7 and ZO1 marker colocalization (at the plasma membrane) indicated translocation of SLAMF7 from lysosomes to the plasma membrane upon ST-5-002 treatment (Fig. 5h). These observations support the hypothesis that TSIs inhibit SLAMF7 degradation due to TM4SF5 binding and lysosomal translocation.

### Anti-TM4SF5 isoxazoles recovered SLAMF7-positive cell populations to block cellular growth in an HCC model

**Fig. 6:**
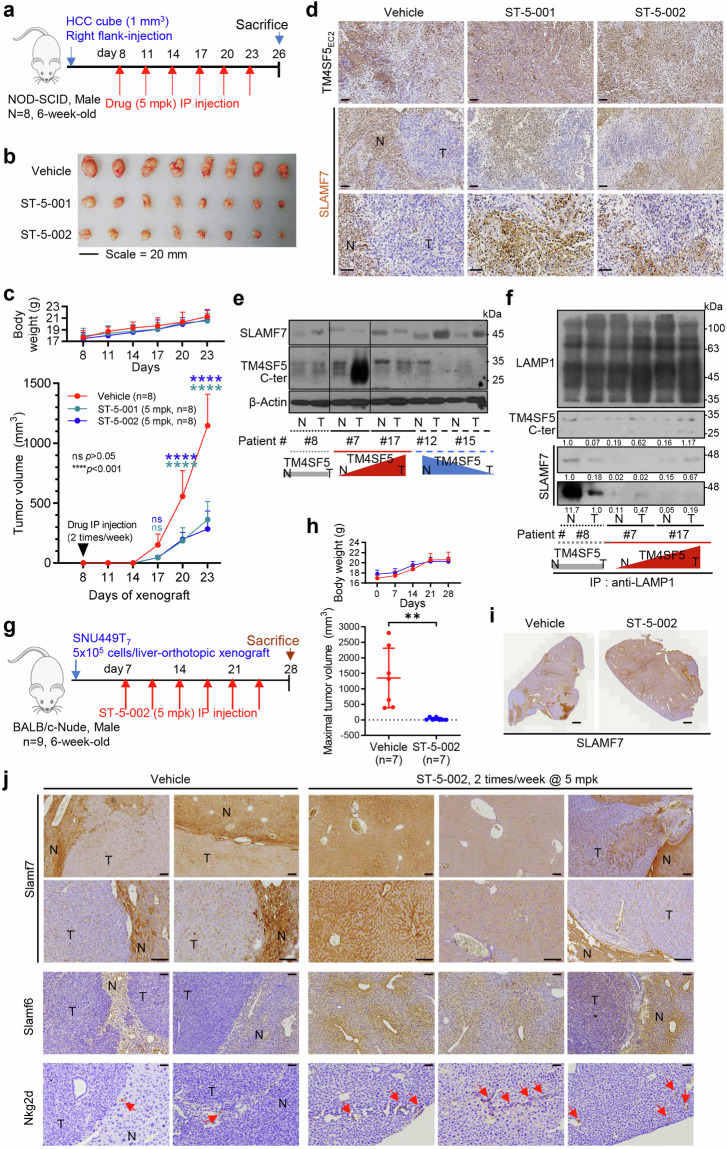
Anti-TM4SF5 isoxazoles recovered SLAMF7-positive cell populations to block cellular growth in an HCC model. **a** Scheme for an HCC PDX xenograft model in NOD-SCID male mice (6-week-old, *n* = 8). **b** Tumors were extracted from the right flank of mice 36 days after tumor inoculation. **c** Tumor size was measured twice a week with a caliper. Tumor weight was measured after sacrifice and tumor extraction. **d** Tumor tissues were immunostained for the indicated molecules. Representative images are shown. **e**, **f** HCC patient tissues [two TM4SF5-positive cases of #7 and #17 (red); two TM4SF5-negative cases of #12 and #15 (blue) and one case of #8 without increased TM4SF5 (gray)] were immunoblotted for TM4SF5 and SLAMG7 (**e**). Whole tissue extracts were processed for immunoprecipitation using anti-LAMP1 antibody before immunoblotting for the molecules (**f**). The ratios of normalized band intensities were compared between conditions. **g** Experimental protocol for an HCC model with liver-orthotopic SNU449T_7_ cell implantation into male BALB/c-Nude mice (*n* = 9). **h** Body weight and maximal tumor volume changes during the liver-orthotopic SNU449T_7_ xenograft experiments. **i** Slamf7-immunostained liver images of tumors from mice with vehicle or ST-5-002 treatment. **j** Liver tissues were analyzed for immunohistochemistry using antibodies against NK cell ligands. Two Slamf7 images in each experimental condition were at two different resolutions. Scale bars, 100 μm. *P < 0.05, **P < 0.01, ns = non-significant, unpaired Student’s *t* test or Two-way ANOVA, Tukey’s multiple comparisons test. Data are represented as the mean ± SEM. See also Figs. S7 and S8

We further investigated the clinical significance of our observations using a TM4SF5-positive PDX model using immune-deficient animals or clinical HCC patient tissue samples. We wondered whether TSI treatment could lead to NK cell surveillance during TM4SF5-mediated HCC development. Starting 1 week after HCC cubes were injected into the right flanks of 6-week-old male NOD-SCID mice (*n* = 8), TSIs were intraperitoneally injected a total of six times at 3-day intervals prior to sacrifice for analyses (Fig. 6a). Compared with vehicle treatment, ST-5-001 or ST-5-002 treatment significantly reduced tumor development without causing body weight loss (Figs. 6b, c). Immunohistochemistry of the tumor tissues showed that TM4SF5 expression was not different among treatments, but Slamf7 expression was more restricted to non-tumor regions in vehicle-treated animals and more widely dispersed, even in smaller tumor lesions, in TSI-treated animals, indicating more SLAMF7-positive hepatocytes and less aggressive HCC growth (Fig. 6d). We further examined the relationship between TM4SF5 and SLAMF7 expressions in HCC patient tissues. HCC tumor tissues that were positive or negative for TM4SF5 (i.e., increased or decreased TM4SF5 levels, respectively, in tumor tissues compared with non-tumor tissues), showed an inverse pattern in their expression (Fig. 6e). Further, we found slightly more TM4SF5 and SLAMF7 in the LAMP1 immunoprecipitates prepared from tumor tissues of TM4SF5-positive patients, compared with those from non-tumor tissues (Fig. 6e, lanes 3 to 6). However, the liver tissues of a patient with insignificantly changed TM4SF5 expression (i.e., TM4SF5-independent) showed less SLAMF7 in the LAMP1 immunoprecipitates from tumor tissue compared with that from non-tumor tissue (Fig. 6f, lanes 1 to 2). Thus, these findings suggest that the TM4SF5-mediated SLAMF7 decrease could involve LAMP1-positive lysosomes also present in HCC tumor tissues.

We next asked whether the hepatic environment would be a barrier to the effects of ST-5-002 in liver-orthotopic SNU449T_7_-xenografts in BALB/c nude mice (Fig. 6g). The liver-orthotopic SNU449T_7_ xenografts produced clear tumor growth, which was inhibited by ST-5-002 without body weight loss (Fig. 6h and Supplementary Fig. S7a). Immunohistochemistry showed increased levels of HCC-related molecules (Ki67, Afp, and Tm4sf5) and inflammatory factors (F4/80, Ccl2, and Ccl20) in the xenografted livers, which were reduced by ST-5-002 (Supplementary Fig. S7b). Furthermore, immunostaining of liver sections showed that Slamf7 stains were mostly restricted to areas outside tumor lesions in the vehicle-treated condition, whereas ST-5-002 treatment resulted in more dispersed Slamf7 stains, presumably indicating the presence of more Slamf7-positive hepatocytes or NK cells throughout the tissue (Fig. 6i). In addition to Slamf7 staining, Slamf6 and Nkg2d immunostaining were more spread out in ST-5-002-treated mice compared with those in mice that did not receive isoxazole treatment, which were more restricted to non-tumor regions (Fig. 6j). To examine whether TM4SF5-mediated tumor formation in immune-competent mice might be also targeted by the TSI, we liver-orthotopically injected SNU449Cp or SNU449T_7_ cells into WT C57BL/6 female mice (*n* = 4) 1 week before starting 2-week treatment with vehicle DMSO or ST-5-002 (Supplementary Fig. S8a). Liver tumors formed following injection of SNU449T_7_ cells but not SNU449Cp cells (Supplementary Fig. S8b). Presumably due to the immune-rejection activity in immune-competent C57BL/6 mice, we might not have more and greater tumor nodules, although the SNU449T_7_ cells (with human TM4SF5 overexpression) have been shown to have an aggressive tumor-initiating cell property.^30^ The livers with tumors formed via TM4SF5-mediated SNU449T_7_ showed lower Slamf7 expression, which was slightly recovered by TSI treatment (Supplementary Fig. S8c). Further, SNU449T_7_-injected livers without TSI treatment insignificantly reduced Prf^+^ splenic NK cells and significantly reduced Slamf7^+^ or Gzmb^+^ intrahepatic NK cells, and these changes were reversed by the ST-5-002 treatment (Supplementary Fig. S8d and S8e, Supplementary Gating Strategy). Thus, these observations support that TSI could abolish the TM4SF5-mediated HCC development via an inhibition of TM4SF5-bound SLAMF7 traffic to lysosome and its degradation, presumably leading to NK cell surveillance.

### The interface of TM4SF5 protein and TSIs

**Fig. 7:**
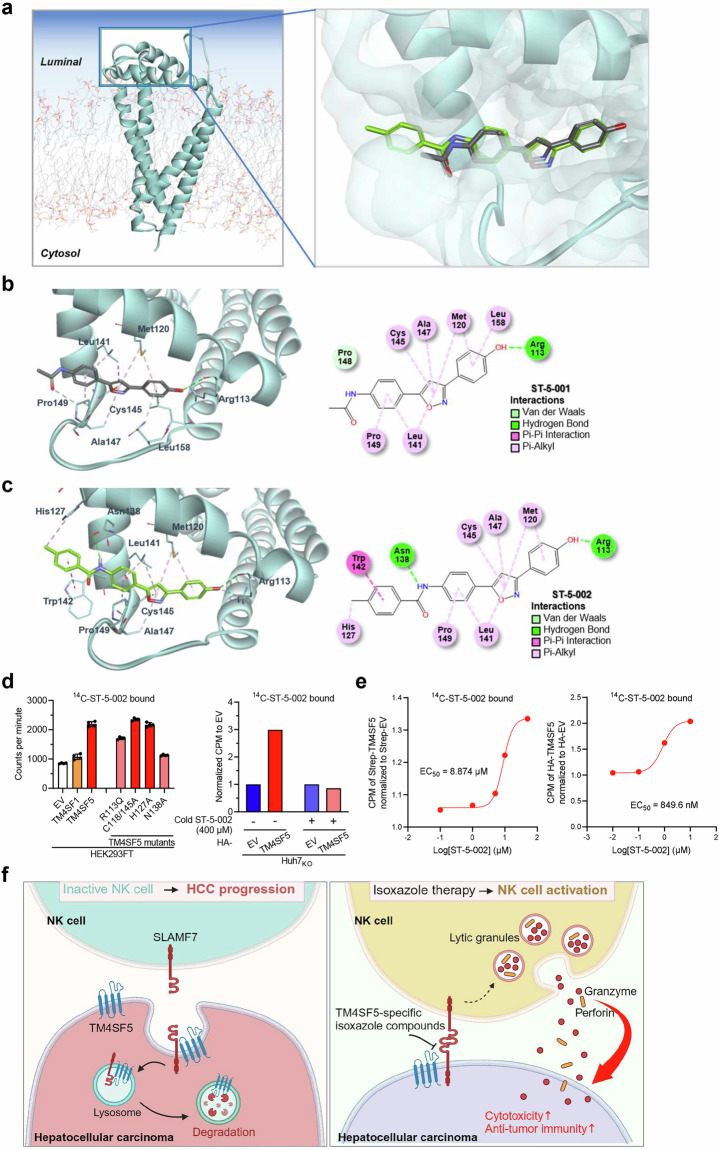
Interface of TM4SF5 protein and TSIs. **a**–**c** Structural modeling of TM4SF5 and TSI binding. **a** Structural model of TM4SF5 (ribbon form) with simulated phospholipid membrane (stick form) depicted with the docking result at the luminal side of the protein. Refined docking models of ST-5-001 (**b**) and ST-5-002 (**c**) show that the isoxazoles fit into the long extracellular loop region positioned at the luminal side of TM4SF5. Both isoxazoles made a hydrogen bond to Arg113, while ST-5-002 made additional favorable interactions toward Asn138 (hydrogen bond) and Trp142 (π–π stacking). **d**, **e** Binding of ^14^C-ST-5-002 to pulled-down (PD) or immunoprecipitated (IP) Strep-TM4SF5 (left) or HA-TM4SF5 (right) from HEK293FT cells or Huh7_KO_ cells, respectively, was assayed after a 1 h incubation. Cold ST-5-002 was pretreated to the reaction mixture for 1 h. The indicated cDNAs were transiently (48 h, left) or stably (right) introduced before TM4SF5 PD or IP. Different concentrations of ^14^C-ST-5-002 were reacted with TM4SF5 PD or IP at the same protein amount for 1 h at 4 °C before β-particle counting. Data represent three isolated experiments. **f** A working model of the inhibitory effects of TSIs on TM4SF5-mediated SLAMF7 downregulation and NK cell inactivation. TM4SF5 in cancerous hepatocytes binds SLAMF7, a stimulatory NK cell ligand, and causes SLAMF7 internalization and trafficking toward lysosomes where it can be degraded, leading to less availability of the ligand for the receptor on NK cells (SLAMF7), resulting in NK cell inactivation and HCC progression (left). TSIs can bind to TM4SF5 and thereby interrupt its binding to SLAMF7, thus preventing SLAMF7 degradation and making SLAMF7 available to its receptor on NK cells. Thereafter, NK cells are activated for the secretion of lytic granules including granzyme and perforin to kill TM4SF5-positive HCC (right). It was generated from online tools of Biorenders

Despite the crucial roles of TM4SF5 in HCC development, questions remain regarding the structural features and capacity for TM4SF5 binding to TSIs. To confirm the specific binding modes of ST-5-001 and ST-5-002 with TM4SF5, we created a homology model of TM4SF5 based on multiple tetraspanin structures. Secondary structure alignment and subsequent structure calculation revealed that TM4SF5 is composed of the typical four alpha helices, which work as the transmembrane region, and a longer extracellular region used to sense lysosomal arginine, as shown previously^33^ (Fig. 7a). However, the long extracellular loop, as the second extracellular domain, adopts a different conformation depending on whether the helical transmembrane region adopts an open or closed form. Docking studies revealed that ST-5-001 and ST-5-002 bind to the spread-out open form of TM4SF5, between the secondary structural motifs within the long extracellular loop and the other parts that presumably shield the motifs. Both TSIs were successfully docked to TM4SF5; however, ST-5-002 showed a stronger interaction with a lower interaction energy of –46.66 kcal/mol versus that of ST-5-001 at –39.32 kcal/mol. This additional affinity is well reflected in intermolecular interactions, where both isoxazoles create a hydrogen bond toward the backbone carbonyl of Arg113. Specifically, ST-5-002 forms an additional hydrogen bond with the side-chain amide of Asn138 and a π-π stacking interaction with Trp142, which implies ST-5-002 as a promising anti-TM4SF5 agent (Figs. 7b, c). Because the anti-TM4SF5 isoxazoles recovered SLAMF7 expression from TM4SF5-mediated downregulation, we next sought to confirm whether ^14^C-labeled ST-5-002 (^14^C-ST-5-002) could bind TM4SF5. TM4SF5 WT efficiently bound to ^14^C-ST-5-002, whereas TM4SF5 R113Q or N138A mutants bound less efficiently, and pretreatment with cold ST-5-002 blocked the ^14^C-ST-5-002 binding to TM4SF5 (Fig. 7d). Differently tagged TM4SF5 proteins were titrated for binding to ^14^C-ST-5-002 following precipitation, resulting in EC_50_ values of approximately 8.874 μM or 849.5 nM (Fig. 7e). These observations support the hypothesis that treatment with TSIs can block the TM4SF5-mediated downregulation and -restricted distribution of stimulatory NK cell ligands, leading to NK cell activation against HCC.

## Discussion

This study reveals that hepatocyte TM4SF5 mediated the downregulation of stimulatory NK cell ligands, including SLAMF7, promoting HCC development. Synthetic TSIs inhibited the binding of TM4SF5 to SLAMF7 and their trafficking to lysosomes, resulting in blockade of TM4SF5-mediated SLAMF7 degradation. TSI treatments eventually inhibited HCC development in animal models by reducing NK cell-based immune evasion (Fig. 7f). Our observations that TM4SF5 promoted HCC in different in-vivo models by downregulating Slamf7 levels and NK cell cytotoxicity suggest that anti-TM4SF5 isoxazoles act as NK cell immune-checkpoint inhibitors (ICIs). Indeed, NK cells account for ~50% of the lymphocytes in the liver.^4^ SLAMF7 stimulates NK cell cytotoxicity as both a ligand and a receptor, and the anti-SLAMF7 agent elotuzumab is approved by the FDA for the treatment of multiple myeloma.^38^ HCC has low cure and survival rates,^39^ and among the various and complicated HCC etiologies, non-viral HCC, particularly MASH-related HCC, is unresponsive to immunotherapy with anti-PD-1 antibody.^17^ Therefore, the possibility that TSIs can suppress the immunosuppressive effect of TM4SF5 on NK cells has clinical implications for the treatment of HCC.

New biomarkers and/or immune checkpoints (ICs) to target HCC within complicated tumor microenvironments need to be identified. Chronic or metabolic dysfunction-associated steatotic liver disease (MASLD) develops as a result of metabolic and immune dysfunction within the complicated hepatic blood system. TM4SF5 is a tetraspan(in) with four transmembrane domains, two (short and long) extracellular loops, one intracellular loop, and a cytosolic N- or C-terminus. Because TM4SF5 is involved in the development of chronic liver diseases, including MASLD,^40,41^ MASH,^25^ fibrosis,^23^ portal hypertension,^42^ and HCC,^21,29^ chalcone-based small molecules^36^ and monoclonal antibodies^22,43^ targeting TM4SF5 have been under development for clinical benefits; systemic or hepatocyte-specific overexpression of TM4SF5 in mice promotes chronic liver diseases in diet- or chemical-mediated liver malignancy models, whereas systemic *Tm4sf5*^−/−^ knockout mice show fewer MASLD features and less HCC development. Although chalcone TSAHC (4’-[*p*-toluenesulfonylamido]-4-hydroxychalcone) showed therapeutic potential to treat TM4SF5-related tumors in mice,^36^ the electrophilic enone system of the pharmacophoric chalcone moiety in TSAHC may render limitations due to its intrinsic cellular toxicity and multiple off-target effects. We thus replaced the enone moiety with aromatic heterocyclic scaffolds, such as isoxazole, isoxazoline, and triazole and modified the two aryl groups in the heterocyclic scaffolds. The resulting new heterocyclic scaffolds were anticipated to provide improved selectivity for TM4SF5 and potency due to their enforced rigidity and high binding affinity.^44^ We created a series of TM4SF5 inhibitors using the 3,5-bisaryl isoxazole framework as the core structure and investigated the function of the 3,5-bisaryl groups for further structural optimization.^45^ In continuation of our effort to find TSIs with improved anti-TM4SF5 activity, we identified several TSIs, including ST-4-005, ST-5-001, and ST-5-002, which were superior to ST-2-001 in terms of inhibitory activity upon structure optimization. Structure optimization, including position switching of the western part (3-Ar) and the eastern part (5-Ar), was carried out based on the SAR analysis of 3,5-bisaryl isoxazole. Among them, ST-5-001 and ST-5-002 were selected as lead compounds for further in-vivo experimental evaluation because they exhibited outstanding inhibitory activities of hepatocyte TM4SF5-mediated signaling. Furthermore, the TSIs have been checked for cardio-toxic risk by hERG assay and pharmacokinetic profiling and were shown to be suitable for the in-vivo studies. Because TM4SF5 is involved in chronic liver diseases, targeting TM4SF5 and its effector SLAMF7 might be possible to directly target HCC or to interrupt the progression from MASH-fibrosis to HCC. Proper biomarkers and therapeutic reagents for MASH and HCC have not been available outside of platforms for combinatory immunotherapies with angiogenesis modulation, so the discovery from this study might be valuable for the development of therapeutic reagents.

There have been several recent immunotherapy trials for advanced HCC, such as IMbrave150^14^ and HIMALAYA.^46^ Although combination regimens of the anti-PD-L1 agent atezolizumab and the anti-VEGF agent bevacizumab resulted in superior overall survival compared with sorafenib, earlier studies suggest that the particular HCC etiology plays a role in modulating the response to these drugs.^47^ The actions of adaptive immune cells in the MASH-HCC transition can lead to a reduction of anti-tumor CD4^+^ T cells and aberrant CD8^+^PD-1^+^ T cell activation within the tumor, possibly leading to hepatocarcinogenesis^48^ and liver damage,^17,49^ respectively. TM4SF5 is also involved in MASH^23,25^ and HCC.^21,43^ Therefore, TM4SF5 and SLAMF7 might serve as therapeutic targets in MASH-related HCC.

TM4SF5, as a member of the tetraspan(in), has characteristics for protein-protein binding and intracellular trafficking,^50–54^ which are important for immune-checkpoint actions.^55^ TM4SF5 expression in hepatocytes is induced by actions of inflammatory cytokines and chemokines^25^ and involved in immune escape,^28^ drug resistance,^56,57^ tumor metastasis^31,32^ and angiogenesis,^35^ via associations with other membrane receptors and/or cytosolic signaling molecules. Furthermore, hepatocyte TRM4SF5 is located at various membrane sources via being associated with different transporters for glucose,^34^ fructose,^40^ amino acids,^33,58^ fatty acids,^59^ or cholesterol.^54^ Thus, the roles of hepatocyte TM4SF5 may involve protein-protein binding and their cellular traffic during HCC development, as speculated in a report.^55^ Although our ongoing project suggests TM4SF5-involved T-cell activation (data not included), macrophage activation and polarization are involved in the TM4SF5-mediated NASH-associated fibrosis.^25^ In addition to transcriptional suppression,^29^ this study reveals a mode of action by which TM4SF5 downregulates SLAMF7 resulting in NK cell inactivation. Here TSIs could act as NK cell immune-checkpoint inhibitors targeting the TM4SF5/SLAMF7 axis, which is highly expressed in HCC.^60^ The inverse linkage between TM4SF5 and SLAMF7 appears valid in the highly TM4SF5-expressing HCC group. However, TSI treatment may be less effective against TM4SF5-independent or TM4SF5-negative and SLAMF7-independent HCC patients, so alternative strategies could involve combination therapies. It is thus worthwhile to explore antibody-drug conjugates or combinations of ICIs to target HCC, involving sorafenib, anti-VEGF(R) reagents, and/or anti-TM4SF5 reagents like TSIs and antibodies.^22,43^

Altogether, HCC with high TM4SF5 expression appeared to be targeted by the TSIs, presumably resulting in SLAMF7-mediated NK cell cytotoxicity, so that TM4SF5-SLAMF7 axis may be considered as NK cell ICs and targets against HCC.

## Materials and methods

### General information for chemistry

Unless noted otherwise, all starting materials and reagents were obtained from commercial suppliers and were used without further purification. All solvents used for routine product isolation and chromatography were reagent grade and glass distilled. Reaction flasks were dried at 100 °C. Air- and moisture-sensitive reactions were performed in an argon atmosphere. Flash column chromatography was performed using silica gel 60 (230–400 mesh; Merck, Darmstadt, Germany) with the indicated solvents. Thin-layer chromatography was performed using 0.25 mm silica gel plates (Merck). Low- and high-resolution mass spectra were recorded using a JMS-700 (JEOL, Akishima, Tokyo, Japan) using fast atom bombardment (FAB) mass spectrometry for **ST-2-001,** **ST-3-001,** **ST-4-005,** **ST-5-001**, and **ST-5-002** and LCQ-Fleet (Thermo Scientific, Waltham, MA, USA) using atmospheric-pressure chemical ionization (APCI) mass spectrometry for ^14^C-labeled **ST-5-002**. ^1^H and ^13^C NMR spectra were recorded on a Bruker Avance III HD spectrometer (800 MHz, with a 5 mm CPTCI CryoProbe; Bruker, Billerica, MA, USA). Chemical shifts are expressed in parts per million (ppm, *δ*) downfield from tetramethylsilane and are referenced to the deuterated solvents (DMSO-*d*_6_ or CDCl_3_ for ^1^H and ^13^C NMR). ^1^H NMR data are reported in the order of chemical shift, multiplicity (s, singlet; brs, broad singlet; d, doublet; m, multiplet), number of protons, and coupling constant (Hz). The purity (>95%) of the final compounds for in vitro and in vivo experiments was confirmed using a SHIMADZU LC-2050C 3D HPLC system (SHIMADZU, Kyoto, Japan).

### Chemistry for isoxazoles

The chemical structures, detailed experimental procedures, spectral data for all synthetic compounds are fully described in Supplementary Chemical Information. They include **ST-2-001 (**Supplementary Chemical Information (CI) 1c and CI 2a), **ST-3-001** (Supplementary CI 1e and CI 2d), **ST-4-005** (Supplementary CI 1j and CI 2b), **ST-5-001** (Supplementary CI 1h and CI 2c), **ST-5-002** (Supplementary CI 1l and CI 2e), and ^14^C-labeled **ST**-**5**-**002** (Supplementary CI 4e and CI 4g). Other parts in Supplementary CI are for synthesized intermediates.

*N-(4-(3-(4-hydroxyphenyl)isoxazol-5-yl)phenyl)acetamide* **(ST-5-001)**: ^1^H NMR (800 MHz, DMSO-*d*_6_) *δ* 10.21 (s, 1H), 9.95 (brs, 1H), 7.82 (d, *J* = 8.7 Hz, 2H), 7.75 (d, *J* = 8.7 Hz, 2H), 7.72 (d, *J* = 8.6 Hz, 2H), 7.33 (s, 1H), 6.89 (d, *J* = 8.6 Hz, 2H), 2.08 (s, 3H); ^13^C NMR (200 MHz, DMSO-*d*_6_) *δ* 169.1, 168.7, 162.3, 159.2, 141.1, 128.1 (2 C), 126.3 (2C), 121.6, 119.4, 119.1 (2C), 115.8 (2C), 97.1, 24.1; LR-MS (FAB+) *m/z* 295 (M + H^+^); HR-MS (FAB+) calcd for C_17_H_15_N_2_O_3_ (M + H^+^) 295.1083, found 295.1089.

*N-(4-(3-(4-hydroxyphenyl)isoxazol-5-yl)phenyl)-4-methylbenzamide* **(ST-5-002)**: ^1^H NMR (800 MHz, DMSO-*d*_6_) *δ* 10.42 (s, 1H), 9.96 (brs, 1H), 7.99 (d, *J* = 8.7 Hz, 2H), 7.90 (d, *J* = 8.1 Hz, 2H), 7.88 (d, *J* = 8.7 Hz, 2H), 7.73 (d, *J* = 8.6 Hz, 2H), 7.38 (s, 1H), 7.36 (d, *J* = 7.9 Hz, 2H), 6.90 (d, *J* = 8.6 Hz, 2H), 2.40 (s, 3H); ^13^C NMR (200 MHz, DMSO-*d*_6_) *δ* 169.1, 165.6, 162.3, 159.3, 141.9, 141.1, 131.8, 129.0 (2 C), 128.1 (2C), 127.8 (2C), 126.1 (2C), 122.0, 120.4 (2C), 119.4, 115.8 (2C), 97.3, 21.0; LR-MS (FAB+) *m/z* 371 (M + H^+^); HR-MS (FAB+) calcd for C_23_H_19_N_2_O_3_ (M + H^+^) 371.1396, found 371.1389.

### Molecular modeling and ligand docking

The sequence of human TM4SF5 (Gene ID: 9032) was aligned to multiple publicly available tetraspanin structures to identify regions with defined secondary structures. A structural model of TM4SF5 was obtained by running the model-based structure calculation module MODELER.^61^ All generated models were clustered by structural conformation to narrow out the closed form of TM4SF5. Additional refinement using explicit solvation and implicit membrane optimization was performed to finalize the structure. The quality of the final protein structure model was evaluated using the Protein Structure Validation Software Suite.^62^ Isoxazole molecules were prepared and docked into a grid-space set at the arginine binding region of TM4SF5 in the long extracellular loop (LEL or EC2).^33^ All residues in the vicinity were designated to be flexible during the docking process. Top-scoring docking results were selected for further TSI-protein interaction analysis. All computational work including model generation and TSI docking was performed using Discovery Studio v.22.1.0.21297 (Biovia, CA).

### Cells

Hepatocyte cell lines (SNU449, SNU761, Huh7, and Hep3B) were purchased from the Korean Cell Bank (Seoul, Korea) and cultured using Roswell Park Memorial Institute-1640 (RPMI-1640) medium or Dulbecco’s modified Eagle medium (DMEM) (Cytiva, Marlborough, MA) containing fetal bovine serum (FBS, 10%) and streptomycin/penicillin (1%, GenDEPOT Inc., Barker, TX). NK92 cells were purchased from the American Type Culture Collection (ATCC, Manassas, VA) and maintained using α-Minimum Essential Medium (MEM) (Invitrogen, Grand Island, NY) supplemented with recombinant human interleukin (IL)-2 (200 U/ml, PeproTech, Rocky Hill, NJ), and fetal bovine or horse serum (12.5%, GenDEPOT Inc.). To generate TM4SF5 knockout cell lines, Huh7 and Hep3B cells at 60–70% confluency were transfected with pSpCas9(BB)-2A-Puro (PX459) V2.0 (62988, Addgene) using polyethylenimine (PEI, 408727, Sigma-Aldrich). The control gRNA targeting sequences were 5′-GGGCCACTAGGGACAGGAT-3′ for adeno-associated virus integration site 1 (*AAVS1*) and 5′-TCCGGGGATTGCAGCCGTT-3′ (#1), 5′-ATTGCAGCCGTTCGGGCAG-3′ (#2), and 5′- GATTGCAGCCGTTCGGGCA-3′ (#3) for *TM4SF5* (exon 2). After 24 h, transfected cells were cultured in a complete medium with 2 μg/ml puromycin, leading to Huh7_KO_ or Hep3B_KO_ cells. To generate NK92 stable cell line, cells were infected with retrovirus encoding pBabe-HAII-control or pBabe-HAII-TM4SF5 and selected by 2 μg/ml puromycin containing complete medium. SNU449 stable cell lines were established as described before.^21^ The cDNA plasmids were transfected with PEI for 48 h. Cells were monitored regularly to prevent mycoplasma contamination.

### Site-directed mutagenesis

Human TM4SF5 WT (Gene ID: 9032) and mutant expression plasmids or viral vectors were explained previously.^23,25,33,34^ Human SLAMF7 was purchased from GenScript (Clone ID: OHu11760, Piscataway NJ) and cloned for various point(s) mutants at the *N*-glycosylation residues of N98, N148, N172, N176, and/or N204 to be Q residues. The mutant names were done with subscripts of the order numbers of the residue; for example, N98/172/176/204Q mutant was presented as NQ_#1456_.

### Mice and chemically induced animal models

C57BL/6 male age-matched WT, hepatocyte-specific TM4SF5 transgenic (TG; *Alb*-TG^Tm4sf5-Flag^), and *Tm4sf5*-knockout (*Tm4sf5*^−/−^, KO) mice were used for in vivo experiments. The animals were as described previously.^23,34^ Mice in a pathogen-free room were kept with humidity (40-60%) and controlled temperature (24 °C ± 2) with light-dark (each 12-h) cycles. Age-matched WT, TG, and/or KO mice (*n* = 3, 6, or *n* ≥ 5, 2-week-old) were injected with 40% olive oil vehicle or DEN [1.0 or 25.0 mg/kg body weight (BW), Sigma-Aldrich, St. Louis, MO] intraperitoneally (IP). In one case, starting 6 weeks after DEN (1 mpk) injection, the mice were IP-injected twice a week with or without CCl_4_ (0.2 mg/kg BW) until they reached 34 weeks of age and sacrificed at 38 weeks (*n* = 3 per group). In other case, the DEN-treated mice were sacrificed at 49 weeks of age (*n* ≥ 5 per group) or at 48 weeks of age after receiving IP-injection of ST-2-001 (5 mg/kg BW) twice a week for the last 6 weeks (*n* = 6). Mice were subsequently euthanized with ether and their livers were collected for analysis. For flow cytometry analyses of splenic or intrahepatic immune cells, liver tissues were homogenized and filtered through 100-μm nylon mesh filters and subjected to Percoll (GE Healthcare) density gradient centrifugation to separate lymphocytes. For immunohistochemistry, resected spleen or liver tissues were incubated in 3.7% formaldehyde (in PBS) at 4 °C. Collections of plasma samples were done for analysis.

### Xenograft

Age-matched-6 weeks-old male BALB/c-nude mice (*n* = 9 per group, Orient Bio Inc., Seongnam, Korea) or NOD.CB17-*Prkdc*^*scid*^/J (NOD-SCID, *n* = 8 or 10 per group; both from Orient Bio Inc.) were liver-orthotopically or subcutaneously injected with SNU449T_7_ cells (5 × 10^5^ cells/mouse) or subcutaneously (at the right flank) xenografted with a human HCC tissue cube (1 mm^3^) for indicated periods and treated with vehicle (DMSO), ST-5-001, or ST-5-002 (2.5 or 5 mg/kg, IP injection, twice/week) as indicated. Age-matched-6-weeks old C57BL/6 female mice (*n* = 4) were liver-orthotopically injected with control SNU449 Cp or TM4SF5-overexpressing SNU449 T_7_ cells (4 × 10^6^ cells/mouse), before sacrifice and analysis of the livers. In cases, the size of tumor xenografts was measured twice a week with a caliper and quantified by following equation: tumor volume = (length × width^2^)/2. Mice were subsequently euthanized with ether and their livers or tumor tissues were collected for analysis.

### Adoptive transfer model

For adoptive transfer experiments with NK92 cells, male NOD-SCID mice (NOD.CB17-*Prkdc*^*scid*^/J, *n* = 10) were subcutaneously injected with 5 × 10^5^ SNU449T_7_ cells at 6 weeks of age. One week later, the mice were intravenously injected with NK92 cells (5 × 10^6^ cells per mouse) expressing empty vector (NK92-EV) or pBabe-HAII-TM4SF5 (NK92-TM4SF5). The NK cell injections were then repeated once a week for 4 more weeks. The tumor size was measured every 3 days. The mice were sacrificed 1 day after the fifth NK cell injection, and liver tissues were analyzed by immunohistochemistry.

### Western blot analysis

Whole-cell or animal liver-tissue extracts were harvested, as previously described.^29^ Primary antibodies against the following molecules were used (generally at 1:1000 dilution): pY^925^FAK (#3284), pT^202^pY^204^ERK (#9101), pY^705^STAT3 (#9145), CD133 (#5860), GLUT4 (#2213), Caspase 3 (#9662), LC3B (#2775), mTOR (#2983), FLAG (#2368), pY^416^c-Src (#2101), GLUT1 (#12939), pT^389^p70S6K1 (#9205), p70S6K1 (#9202), ERK (#9102), HA (#3724; all from Cell Signaling Technology. Danvers, MA), α-tubulin (sc-5286), MICA/B (sc-137242), IL-6Rα (sc-661), pY^861^FAK (sc-16663), β-ACTIN (sc-47778), SLAMF7 (sc-390840), EGFR (sc-03), STAT3 (sc-8019), pY^473^AKT (sc-7985), Cyclin D1 (sc-753), CD44 (sc-7287), pY^577^FAK (sc-16665; all from Santa Cruz Biotechnology, Santa Cruz, CA), pY^397^FAK (611723), FAK (610088), p27^Kip1^ (610242; all from BD Transduction Laboratories, Bedford, MA), IL6Rα (MAB227; from R&D system), SLAMF6 (ab224201), TIGIT (ab233404), pS^10^p27^Kip1^ (ab62364; all from Abcam, Cambridge, UK), NKG2D (PA5-102038; from Thermo Fisher Scientific), CD155 (337601; from BioLegend, San Diego, CA, USA), Granzyme (A2557), Perforin (A0093; both from ABclonal, Woburn, MA), integrin α5 (MAB1956Z; from Millipore, Billerica, MA), StrepMAB-classic (HRP conjugate; from IBA, Cat#2-1509-001), and TM4SF5.^34^

### Cytotoxicity of NK cells

NK cell cytotoxicity was analyzed with the Cytotoxicity Detection Kit Plus kit to measure amounts of lactate dehydrogenase (LDH) (Roche). Huh7_KO_ cells harboring empty vector (EV), TM4SF5, or SLAMF7 plasmids were seeded as target cancer cells (0.5 × 10^4^ cells/well; 50 μl) in triplicate into 96-well plates with assay medium (1% FBS-containing RPMI 1640). Effector NK92-EV or NK92-TM4SF5 cells were prepared by IL-2 pretreatment at a density of 1 × 10^6^ cells/ml using the assay medium. The effector cell suspension was mixed with target cells at ratios of effector (E)-to-target (T) cells (1.25:1, 2.5:1, 5:1, or 10:1). The effector and target cells were incubated for 4 h at 37 °C in 5% CO_2_. Using an ELISA reader, LDH activity from damaged cells was measured at 492-690 nm wavelength. Calculations of cytotoxicity (%) were done as follows^29^: [LDH (E-T cell mix)−LDH (E cell)−LDH (T cell low control)]÷[LDH (T cell high control)−LDH (T cell low control)] × 100.

### Murine intrahepatic NK cell analysis

NK cells from mouse liver or spleen tissues were analyzed as previously described.^29^ Livers from 5-month-old or 1-year-old female C57BL/6 mice were dissected, homogenized by mechanical disruption and filtered serially through 100 and 70-μm nylon mesh filters. Lymphocytes were separated via a Percoll (37.5%) density gradient centrifugation. Isolated immune cells were suspended in 2% FBS-containing RPMI 1640 medium and centrifuged (480 × *g*, 8 min). To eliminate RBC in the pellets, RBC lysis buffer (Thermo Fisher Scientific, Cat. No: A1049201) was added for an incubation (5 min, 37°C). After two washes of pellets using PBS with 2% FBS, cells were incubated with anti-mouse Cd16/Cd32 (mouse BD Fc Block™, BD Bioscience, Cat. No: 553142) according to standard protocol to block FcγII/III receptors on lymphocytes. Lymphocytes were then incubated with phorbol 12-myristate 13-acetate (5 ng/ml), ionomycin (500 ng/ml), and monensin (10 ng/ml, Sigma) for 4 h in a CO_2_ incubator, to measure the expression of Ifn-γ, perforin 1 (Prf1), or granzyme B (Gzmb). Blocking FcγII/III receptors with mouse BD Fc Block™ was followed by staining of surface antigens with fluorophore-conjugated monoclonal antibodies (mAbs). For intracellular staining, intrahepatic or splenic immune cells were fixed using BD Cytofix/Cytoperm™ Fixation/Permeabilization Kit (BD Bioscience, Cat. No: 554714) and then stained using fluorophore-conjugated mAbs at 4 °C for 30 min. Antibodies used included allophycocyanin (APC) anti-mouse Gzmb, PE anti-mouse Prf1, fluorescein isothiocyanate anti-mouse Cd45, APC/Cy7 anti-mouse Cd3 (BioLegends), PE/Cy7 anti-mouse Cd319 (Slamf7, Cell Signaling), and BV421 anti-mouse Nk1.1 (BD Bioscience). Flow cytometry was done on a MACSQuant® Analyzer 10 (Miltenyi Biotec, Bergisch Gladbach, Germany) and analyzed using FlowJo (version 10.6.1). Data values were graphed as the mean ± the standard error of the mean (SEM). FACS Gating strategies were explained in Supplementary materials.

### Immunohistochemistry

Liver tissues from mouse HCC models or patients with HCC were processed for immunohistochemistry. For immunostaining antibodies used including anti-TM4SF5,^21^ human/mouse SLAMF7 (CS1, sc-390840, Santa Cruz Biotech), human TIGIT (99567s, Cell Signaling Technology), human/mouse NKG2D (PA5-102038, Thermo Fisher), and mouse Tigit (ab233404, Abcam, UK). Antigens were developed with the Vectastain ACB kit (Vector), before staining with 3,3′-diaminobenzidine. Hematoxylin and eosin stains of liver tissues were performed, as previously described.^29^ Ten random images from a slide were captured by a digital slide scanner (MoticEasyScan, Motic, British Columbia, Canada).

### Immunofluorescence

Cells were seeded on 10 μg/ml fibronectin (35600, BD Biosciences) pre-coated cover glasses and incubated for 16 h. The cells were transfected for 24 h with the indicated expression plasmids using PEI with or without TSI treatment. The cells were then fixed with ice-cold 99% methanol for 15 min and incubated with blocking solution (1% BSA in PBS) for 1 h at room temperature. For immunostaining, primary antibodies were used including anti-FLAG (NB600-344, Novus Biologicals), anti-LAMP1 (#9091, Cell Signaling Tech,), anti-ZO-1 (402200, Invitrogen), anti-HA (901515, BioLegend), and anti-SLAMF7 (sc-390840, Santa Cruz Biotech), diluted in blocking solution (1:500). After 3 washes with PBS, cells were incubated with fluorescence-conjugated secondary antibodies [Alexa Fluor 488 (goat: A11055 and mouse: A21202) and 555 (goat: A21432 and mouse: A31570), both from Invitrogen, 1:500 dilution] for 1 h at room temperature. After 3 washes with PBS, the cover glasses were mounted on slide glasses using ProLong^TM^ Gold Antifade (P36930, Invitrogen). Images were captured randomly using a Nikon Eclipse Ti microscope with a C2 confocal system and analyzed by NIS-Elements software (Nikon, Melville, NY, USA). To validate colocalization of SLAMF7 with LAMP1 or ZO-1, Pearson’s correlation coefficients were calculated using NIS-Elements software. All images presented in the same figure panel were taken under the same software settings and identically processed in Adobe Photoshop. Each dot as a datum in the graphs indicates a measurement from one cell unless otherwise indicated.

### Immunoprecipitation and in vitro pulldown

Cells were lysed in Brij58 lysis buffer (20 mM HEPES, pH 7.4, 150 mM NaCl, 2 mM MgCl_2_, 2 mM CaCl_2_, and 1% Brij58). Whole-cell lysates were incubated with streptavidin-agarose (20353, Thermo Fisher Scientific) for 4 h at 4 °C. Beads were washed three times with ice-cold lysis buffer and then twice with ice-cold PBS. Washed beads were eluted in 2× SDS-PAGE sample buffer and boiled for 5 min before immunoblot analysis.

### ^14^C-ST-5-002 binding assay

^14^C-labeling of ST-5-002 was done commercially and confirmed via NMR analysis (99.7% radio-chemical purity and 1.97 GBq/mmol activity; Curachem, Inc., Cheongju-si, Republic of Korea). TM4SF5-lacking HEK293FT cells were transiently transfected with Strep-EV, Strep-TM4SF1, or Strep-TM4SF5 WT or mutant cDNAs for 48 h. In addition, Huh7_KO_ cells were stably transduced with retrovirus encoding pBabe-HA-EV or pBabe-HAII-TM4SF5. Whole-cell lysates were prepared, and lysates containing 0.2 mg or 0.25 mg protein were processed for pulldown or immunoprecipitation using streptavidin-agarose or anti-HA antibody plus protein A/G-agarose beads for 4 h at 4 °C. The processed lysates were treated for 1 h with or without cold ST-5-002 (400.0 μM) and then incubated for 1 h at 4 °C with ^14^C-ST-5-002 (10.0 μM or 25.0 μM) before β-particle counting using a TriCarb scintillation counter (PerkinElmer, Waltham, MA). For EC_50_ measurement, different ^14^C-ST-5-002 concentrations were incubated with pulldown or immunoprecipitation products from lysates containing 1 mg protein.

### Sphere growth analysis

Cells were cultured in ultra-low attachment six-well plates (Corning Inc. Corning, NY) at a density of 1 × 10^3^ cells/well for the indicated periods with 2.5 μM TSI treatment every 2–3 days. Daily images were obtained using phase contrast microscopy (CKX41, Olympus, Tokyo, Japan).

### qRT-PCR

The cDNAs were prepared using qPCR RT Master Mix (ReverTra Ace, TOYOBO) from RNA templates of animal liver tissues or cells. Quantitative RT-PCR was done using CFX Connect RT PCR Detection System (Bio-Rad, Hercules, CA). Normalization of the mRNA levels was done against 18S ribosomal RNA by the ddCq method, and then analyzed with the CFX Maestro Software (Sunnyvale, CA). Primers shown in Table 1 were synthesized by Cosmo Genetech (Seoul, Korea).

**Table 1:** The primer sequences for qRT-PCR in the current study

Gene name	Sequence	
	Forward (5′ → 3′)	Reverse (5′ → 3′)
Mouse		
*Tm4sf5*	GTCTTCTCCTCCGCCTTTG	GGTAGTCCCACTTGTTGTCTATT
*Slamf7*	CATCGAGTCAGGCTTCCTTAAT	CTTGTTGCTTTGCCGATCTATG
*Nkg2d*	GATGGCTCCTCTCTCTCATACA	GCCTTAAAGCTTGAGCCATAGA
*Crtam*	CAGCTACACAACTCTCCATTAGT	ACTCTCACTTGCTTCGTCTTC
*Dnam-1*	AGACGGAGACAGGTGAGAAT	TAGACTGGATGGGAGAAGTAGG
Human		
*MICA*	CCTTGGCCATGAACGTCAGG	CCTCTGAGGCCTCGCTGCG-
*MICB*	ACCTTGGCTATGAACGTCACA	CCCTCTGAGACCTCGCTGCA
*ULBP1*	TTTCCTTAAAGGGCAACTGCT	AGGAACTGCCAAGATCCTCT
*CADM1*	GCTTCTGCTGTTGCTCTTCT	CTCGATCACTGTCACGTCTTTC
*VCAM1*	GGAGCTCTACTCATTCCCTAGA	CTAGGAACCTTGCAGCTTACA
*PD-L1*	CAGCAACCAGACGGACAA	TGACTTCCACATGAGCGTG
*TIGIT*	TCTATCACACCTACCCTGATGG	AGATTCCATTGCTTGGAGCC

### Differential gene expression (DEG) analysis

Statistical significance of DEG data using RNA-Seq datasets from WT or *Tm4sf5*^-/-^ C57BL/6 male mice at 3-month old (Bioproject ID: PRJNA1144602) was determined by nbinomWaldTest using DESeq2. The false discovery rate (FDR) was controlled by adjusting *p*-values using the Benjamini-Hochberg algorithm. Gene-enrichment, functional annotation, and pathway analyses of the significant gene list were performed using gProfiler (https://biit.cs.ut.ee/gprofiler/orth) and KEGG pathways (http://www.genome.jp/kegg/pathway.html). Image visualization was done with ggplot2.

### Analysis of public RNA expression data

The relationships between overall patient survival and expression levels of TM4SF5, SLAMF7, and other genes were determined by Kaplan-Meier analysis and log-rank test using OncoLnc (http://www.oncolnc.org/). Data from 360 patients with liver hepatocellular carcinoma (LIHC) were available in The Cancer Genome Atlas (TCGA) and were used for OncoLnc analysis. For the analysis of each gene, patients were divided into the top 50% expression group (*n* = 180) and the bottom 50% expression group (*n* = 180) based on the expression levels of *TM4SF5*, *SLAMF7*, or other genes. For analysis of the functional relationships between *TM4SF5* and *SLAMF7* or other ligands, the top 25% TM4SF5 expression group (*TM4SF5*^high^, *n* = 90) were selected from the LIHC data and then divided into the top 25% or bottom 25% expression groups of each gene (e.g., *SLAMF7*^high^, *n* = 45 or *SLAMF7*^low^, *n* = 45) for the analysis of probability of survival. The hazard ratios and *p*-values were estimated by Mantel–Haenszel and Mantel–Cox tests. Data visualization was conducted in Prism 9.0 and the R (version 4.3.3) programming environment using the pheatmap package.

### Ethics approval

All animal procedures were approved by the IRB of the Institutional Animal Care and Use Committee of the Seoul National University (SNU-190122-6-3, SNU-221117-3-1, SNU-221229-2, and SNU-230308-3-1). All animal experiments were performed under the reporting of In Vivo Experiments (ARRIVE) guidelines. HCC tissue samples and PDX models using cancer tissues from patients with HCC at Seoul National University Hospital were established and used following IRB approval (H-2102-059-1195).

### Statistics

Prism Software (GraphPad version 7.0, La Jolla, CA) was used for statistical analysis. To determine the normality of data, D’Agostino-Pearson omnibus test was performed. For statistical analysis of normally distributed data, ordinary one-way analysis of variance (ANOVA); Two-way ANOVA; Tukey’s multiple comparisons test; unpaired Student’s *t*-test was performed. Unpaired, one-tailed Mann-Whitney U test was performed for non-normal data. Data with *p*-value < 0.05 were considered statistically significant.

## Supplementary information

Supplementary Materials

TM4SF5_homology model

Supplementary Uncut immunoblot gel images

## Data Availability

The data and materials can be available upon written request to the corresponding author. RNA-Seq datasets have been deposited in the NCBI Sequence Read Archive (SRA) under the project number PRJNA1144602, in accordance with the journal’s publication policy.

## Supplementary information

The online version contains supplementary material available at 10.1038/s41392-024-02106-6. Supplementary information accompanies the manuscript on the *Signal Transduction and Targeted Therapy* website http://www.nature.com/sigtrans.
