# Supplementary material for: Isoxazole-based molecules restore NK cell immune surveillance in hepatocarcinogenesis by targeting TM4SF5 and SLAMF7 linkage
Source: Signal Transduct Target Ther. 2025 Jan 20;10:15. doi: 10.1038/s41392-024-02106-6 (PMC11743776; doi:10.1038/s41392-024-02106-6)

Supplementary Materials for

Targeting TM4SF5-mediated SLAMF7 downregulation by isoxazole-based small molecules restores NK cell immune surveillance during hepatocarcinogenesis

Ji Eon Kim[1,2,†], Hyun Su Kim[3,†], Wonsik Kim[1,†], Eun Hae Lee[1], Soyeon Kim[1], Taewoo Kim[3], Eun-Ae Shin[1], Kyung-hee Pyo[1], Haesong Lee[1], Seo Hee Jin[1], Jae-Ho Lee[1], Soo-Min Byeon[1], Dong Joo Kim[1], Jinwook Jeong[1], Jeongwon Lee[1,2], Minjae Ohn[1,2], Hyojung Lee[1,2], Su Jong Yu[4], Dongyun Shin[5], Semi Kim[6], Jun Yeob Yoo[7], Seung-Chul Lee[7], Young-Ger Suh[3,*], and Jung Weon Lee[1,2,*].

Correspondence to: ygsuh@cha.ac.kr and jwl@snu.ac.kr

This PDF file includes:

Figures, S1 to S8
Chemical Information, CI I to V
Supplementary Gating Strategy GS1 to GS4

Other Supplementary Materials for this manuscript include the following:

Supplementary uncut immunoblot gel images, Fig.S1a to S8c.

Figure. S1.

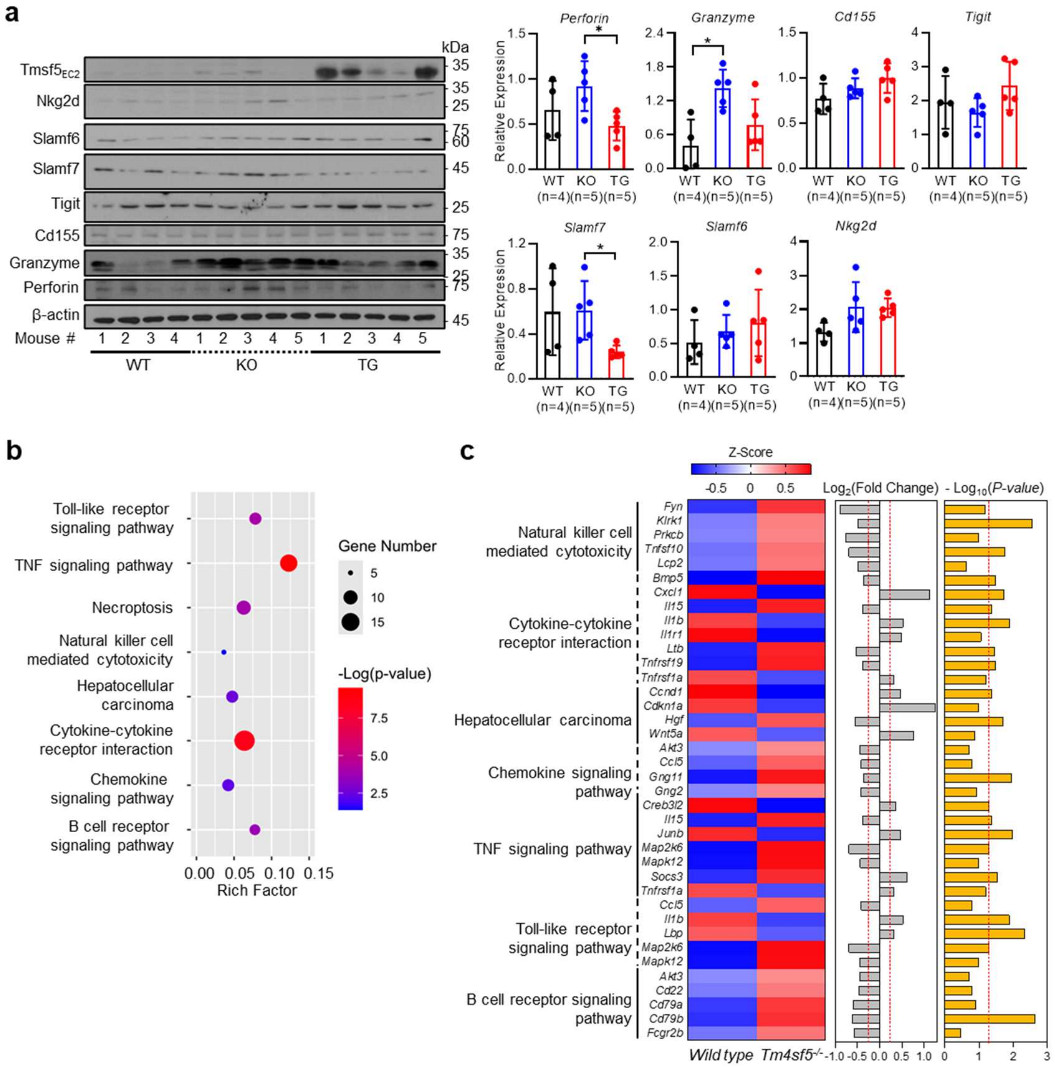

Figure S1. Differential gene expression (DEG) and functional pathway between 3-month-old WT or KO (*Tm4sf5*$^{-/-}$) C57BL/6 mice. (a) Liver tissues extracted from male 3-month-old WT, *Alb*-TG$^{Tm4sf5\text{-}Flag}$ (TG), or *Tm4sf5*$^{-/-}$ KO C57BL/6 mice were subjected to immunoblots for the indicated molecules. (Right) Band intensities of the immunoblot were determined and normalized against loading control or total counterpart protein for graphic presentations. *P* values were calculated by one-way ANOVA, Kruskal-Wallis multiple comparison test. * $p < 0.05$. **(b)** Differentially expressed genes in RNA-Seq datasets from 3-month-old male WT or *Tm4sf5*$^{-/-}$ KO C57BL/6 mice (n=4) were analyzed with regards to KEGG pathways. For each pathway, the number of genes with significant differential expression (*p*-values) and the Rich factor (significantly differential gene numbers over total gene numbers) are presented. Data represent three independent experiments. **(c)** RNA-Seq datasets from WT or KO mice (n=4, PRJNA1144602) were included for the DEG. Red dotted lines mean | Log_2(Fold Change) | = 0.301 and – Log_{10}(*P-value*) = 1.30. See also Figure 1.

Figure. S2.

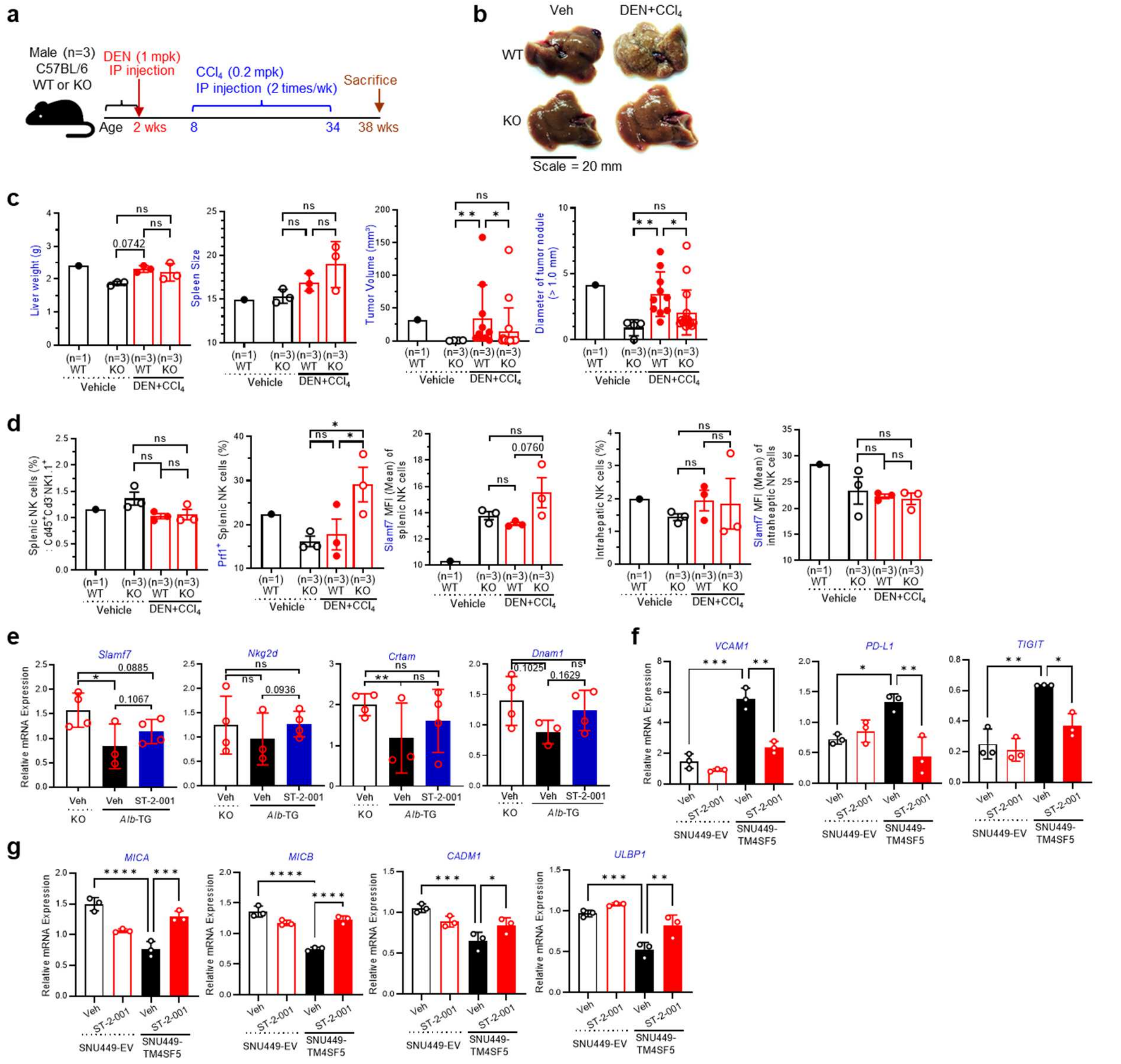

Figure S2. TM4SF5-mediated NK cell inactivation and NK cell ligands regulations were reversed by anti-TM4SF5 isoxazoles. (a–d) Scheme for liver cancer models with DEN+CCl_4 administration in male WT or *Tm4sf5*$^{-/-}$ KO C57BL/6 mice (n=3). Only one WT mouse with DEN administration alone was used (n=1) because of injection-mediated death, such that the datum is shown to indicate the level but not for any comparisons (a). Representative liver images. Scale bar = 20 mm (b). Liver and spleen parameters and tumor volume and diameter (a longer diameter in mm) were analyzed (c) before processing for analyses of intrahepatic NK cell populations and Prf1 and Slamf7 immunostaining of NK cells. Scale bars, 100 μm (d). **(e)** Liver tissues from liver cancer models of DEN administration in the presence of vehicle or ST-2-001 treatment in WT, *Alb*-TG$^{\text{Tm4sf5-FLAG}}$ (TG), or *Tm4sf5*$^{-/-}$ KO C57BL/6 mice (n≥5) were processed for qRT-PCR. **(f–g)** SNU449-EV or SNU449-TM4SF5 cells were treated with vehicle or ST-2-001 (2.5 μM) for 24 h, before qRT-PCR analysis against the indicated molecules including inhibitory (f) or stimulatory

(g) NK cell ligands. One-way analysis of variance (ANOVA) test was performed to determine statistical significance. A value of $p < 0.05$ was considered statistically significant. *, **, ***, or **** depict statistical significance with $p<0.05$, 0.01, 0.001, or 0.0001, respectively. ns indicates non-significance, unpaired Student's *t* test or two-way ANOVA. Data are represented as the mean ± SEM. Data represent three independent experiments. See also Figure 1.

Figure. S3.

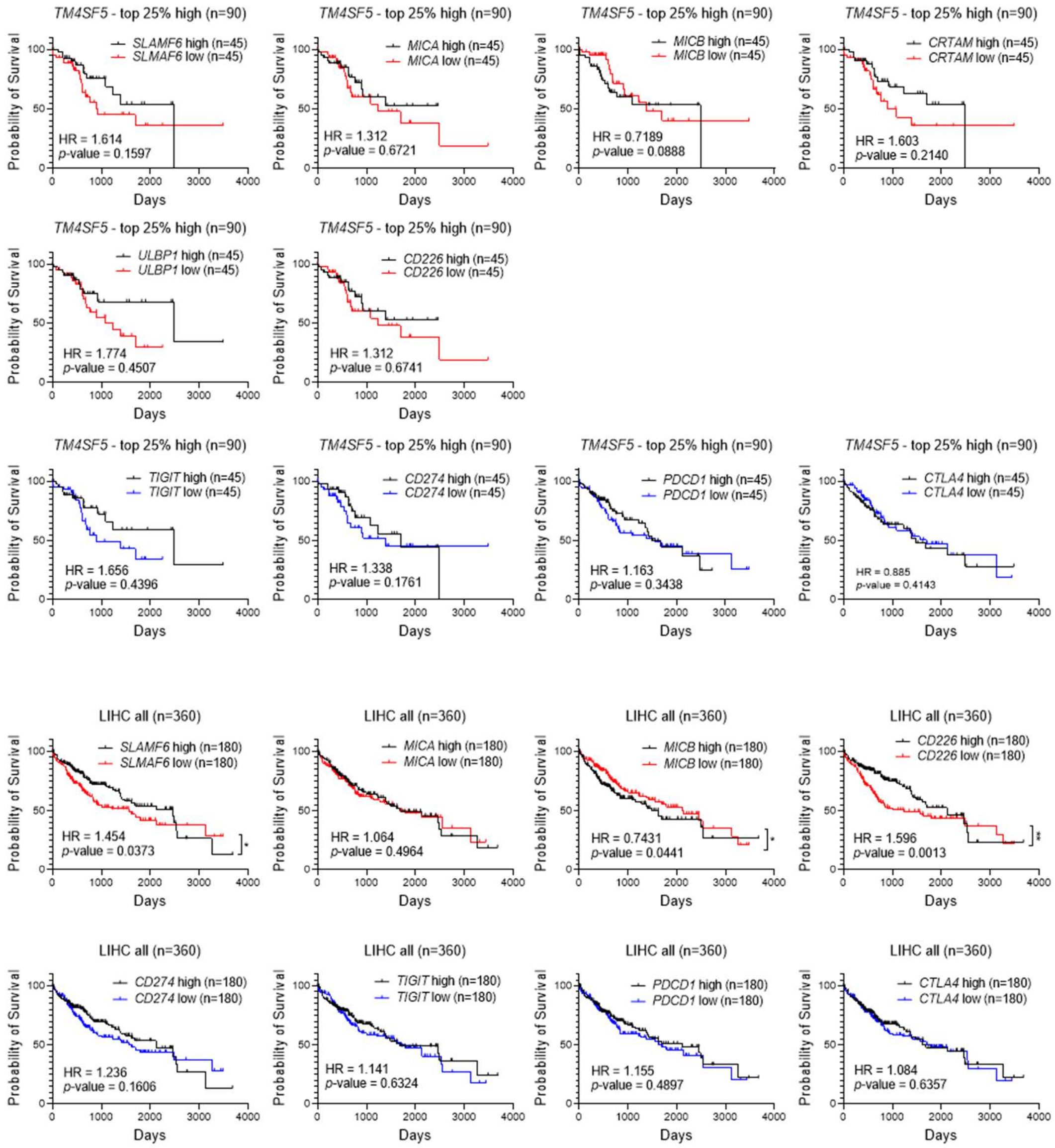

Figure S3. Relationship of expression of each NK cell ligand with TM4SF5 with regards to overall survival rate in liver cancer patients of TCGA dataset. Data from 360 hepatocellular carcinoma (LIHC) patients of TCGA dataset were analyzed. For analysis of the functional relationships between *TM4SF5* and *SLAMF7* or other ligands, the top 25% TM4SF5 expression group (*TM4SF5*^{high}, n=90) out of the LIHC data were selected and then divided into top 25% or bottom 25% expression groups of each gene (e.g., *SLAMF7*^{high}, n=45 or *SLAMF7*^{low}, n=45) for analysis of probability of survival. Meanwhile, for the analysis of each gene, patients were divided into the top 50% expression group (n=180) and the bottom 50% expression group (n=180) based on the expression levels of each gene. All the cases shown in this figure did not show any significant difference in overall survival of LIHC patients. However, differential overall survival rate was significantly observed as for *TM4SF5*^{high} and *SLAMF7* expression (See Figure 1l).

Figure. S4.

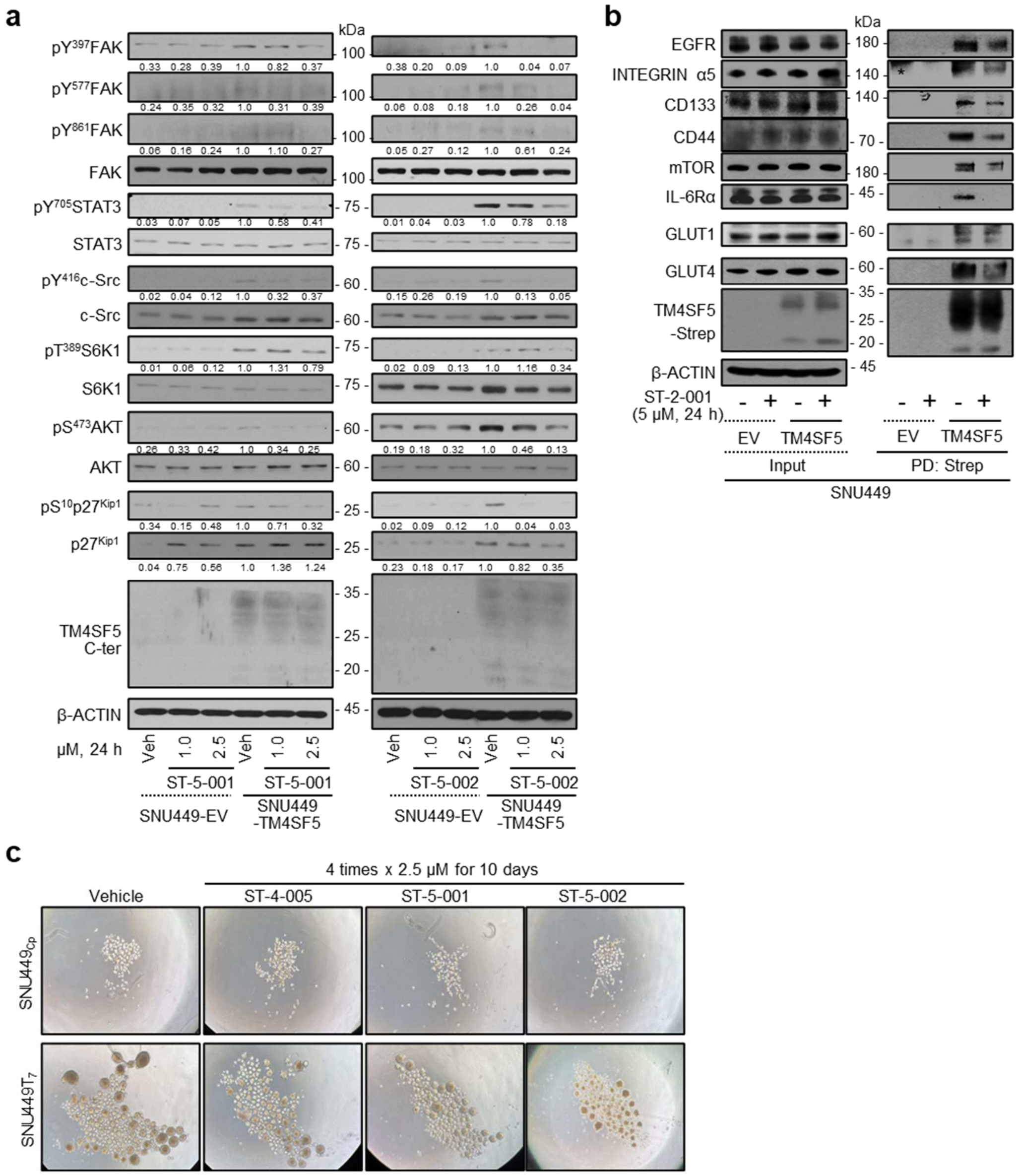

Figure S4. Blockade of anti-TM4SF5 isoxazoles in TM4SF5 binding to diverse membrane receptors and TM4SF5-dependent 3-dimensional sphere growth. (a-b) stable SNU449-EV or SNU449-TM4SF5 cells were treated with vehicle (-) or ST-5-001, ST-5-002 (1.0 or 2.5 µM), or ST-2-001 (2.5 µM) for 24 h, before harvests of whole cell lysates. Cell lysates were processed to Western blots for the indicated molecules (a). Meanwhile, pulldown (PD) using streptavidin-agarose beads were done and immunoblotted against the indicated molecules in parallel with the cell lysates as input. * indicates non-specific band (b). **(c)** SNU449$_{Cp}$ or SNU449T$_7$ cells on ultra-low attachment plates were cultured for 10 days while every 2-3 days vehicle DMSO or anti-TM4SF5 isoxazoles (2.5 µM) were treated finally 4 times. Representative images were presented. Data represent three independent experiments. See also Figure 2.

Figure. S5.

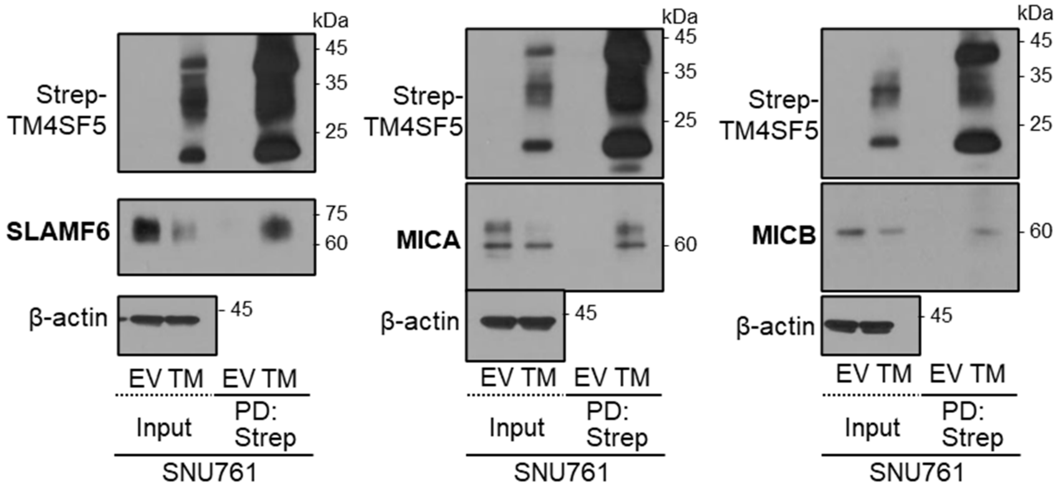

Figure S5. Binding of TM4SF5 in hepatocytes to stimulatory NK cell ligands. SNU761 cells expressing empty vector (Strep-EV) or Strep-TM4SF5 in a sub-confluent condition were processed for whole cell lysate preparation. The lysates were processed for pulldown (PD) using streptavidin-agarose beads before standard immunoblotting in parallel with input blotting against the indicated molecules. Data represent three independent experiments. See also Figure 4.

Figure. S6.

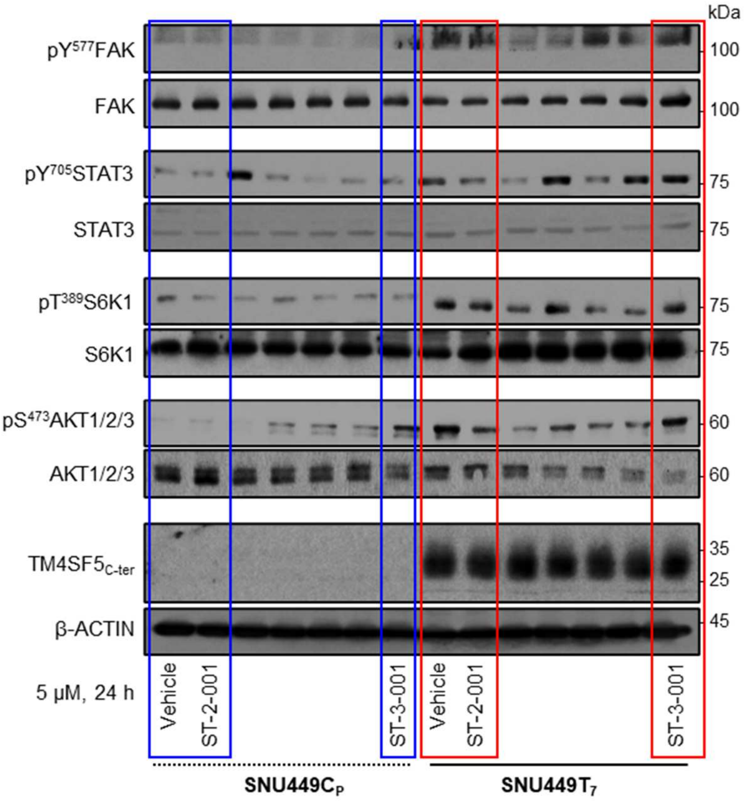

Figure S6. Negative control or anti-TM4SF5 isoxazoles with regards to TM4SF5-mediated signaling. SNU449$_{Cp}$ or SNU449T$_7$ cells in a sub-confluent condition were treated with DMSO or isoxazoles (2.5 μM) for 24 h, prior to whole cell lysate preparation. The lysates were processed for standard immunoblots against the indicated molecules. Data represent three independent experiments. See also Figure 5.

Figure. S7.

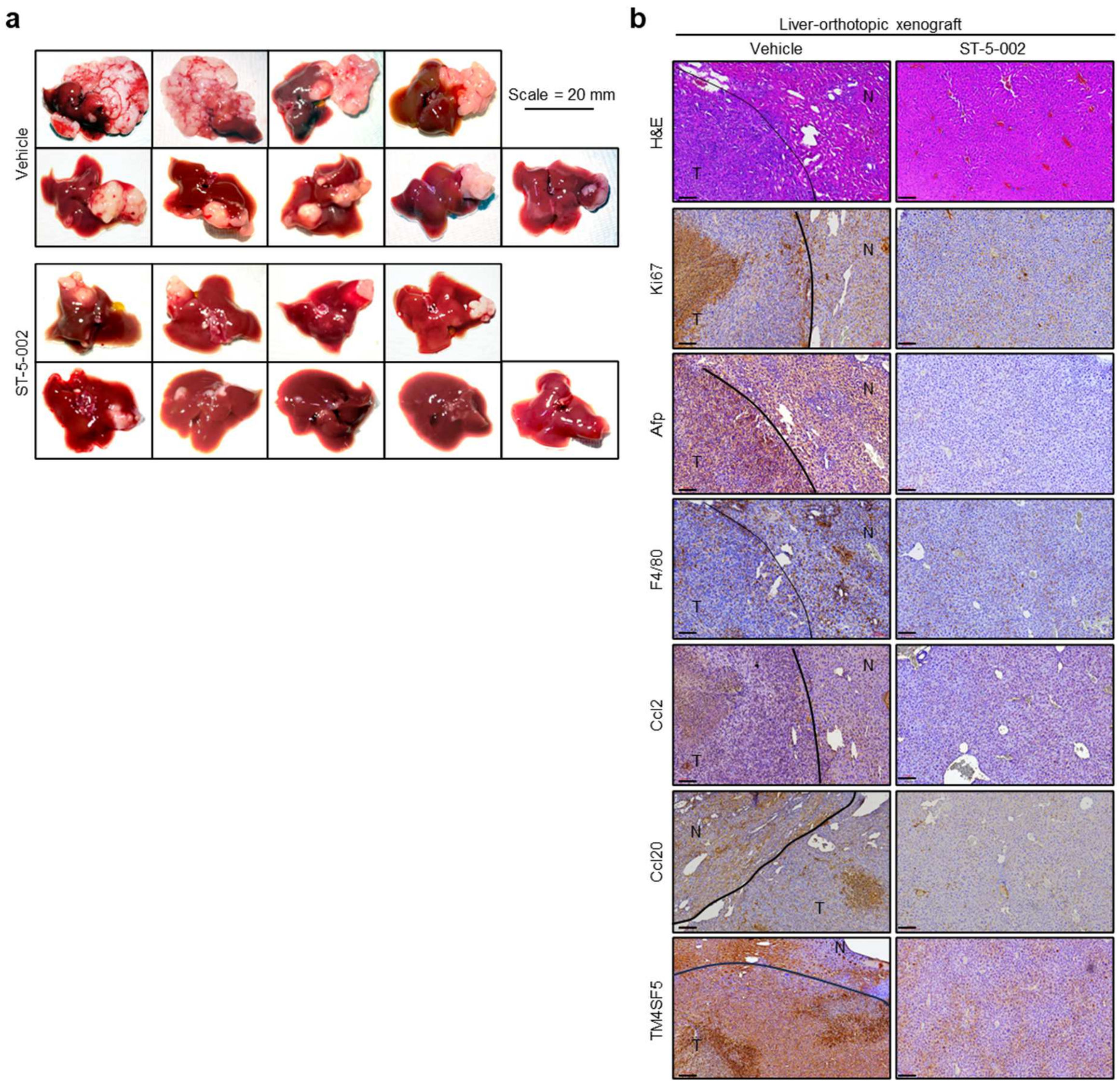

Figure S7. Blockade of anti-TM4SF5 isoxazole ST-5-002 in tumor development by liver-orthotopic SNU449T$_7$-xenografts to BALB/c-Nude mice. (a) Experimental protocols using male BALB/c-Nude mice (n=9) with SNU449T$_7$ cells implanted liver orthotopically, as depicted in Fig. 6h. Representative images of livers were from mice with vehicle or ST-5-002 treatment. **(b)** Liver tissues were analyzed for the immunohistochemistry using the antibodies against the indicated molecules. Representative images from multiple images of multiple mice from the experimental conditions. Scal bar: 100 μm. See also Figure 6.

Figure. S8.

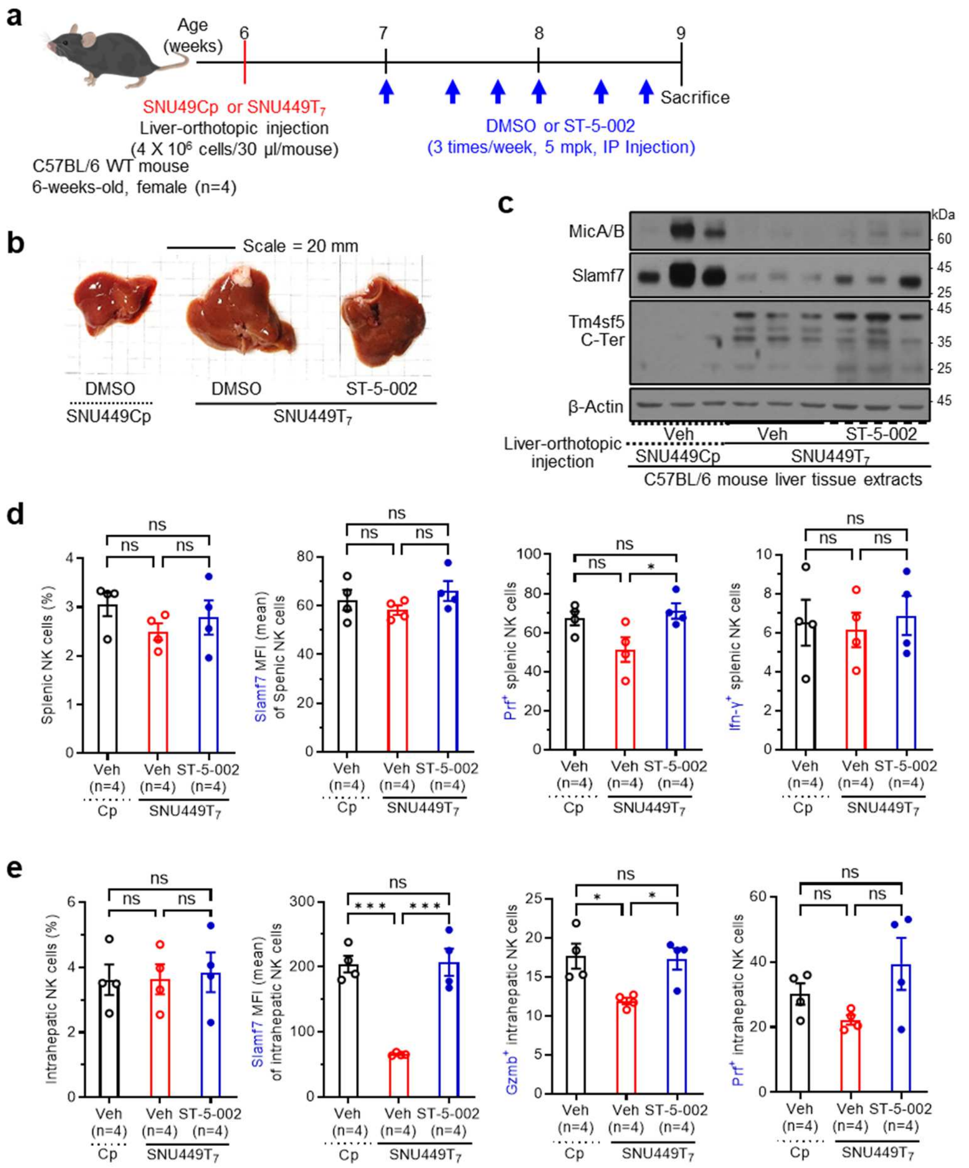

Figure S8. Blockade of anti-TM4SF5 isoxazole ST-5-002 in tumor development by liver-orthotopic SNU449T$_7$-xenografts to C57BL/6 mice. (a) Experimental protocols using WT C57BL/6 female mice (n=4) with SNU449T$_7$ cells implanted liver orthotopically. **(b)** Representative images of livers were from mice with vehicle or ST-5-002 treatment to SNU449Cp or SNU449T$_7$-xenografts. The tissues were on a graduation paper, whose graduation was replaced with a scale (10 mm) and blocked whitely. **(c)** Immmoblots of the indicated molecules from whole liver extracts. Multiple bands in TM4SF5 immunoblot depict *N*-glycosylated TM4SF5 proteins. **(d-e)** Liver tissues were analyzed to measure splenic (d) or intrahepatic (e) NK cell populations and intracellular staining for Prf$^+$, Gzmb$^+$, or Ifn-γ$^+$ NK cells. *, p<0.05. ns indicates non-significance, One-way ANOVA. Data are represented as the mean ± SEM. See also Figure 6.

Supplementary Chemical Information

List of Contents

I. Experimental procedure for TM4SF5-specific isoxazole (TSI)-based small molecules … p12 – p16

II. ^{1}H and ^{13}C NMR spectra of the synthesized intermediates and products …………… p17 – p28

III. HRMS data of final compounds …………………………………………………… p29 – p31

IV. HPLC purities of final compounds ………………………………………………… p32 – p34

V. Preparation and analysis of [^{14}C]-labeled ST-5-002 radio-active compound ……… p35 – p39

I. Experimental procedure for TM4SF5-specific isoxazole (TSI)-based small molecules

*1. Synthesis of **ST-2-001***

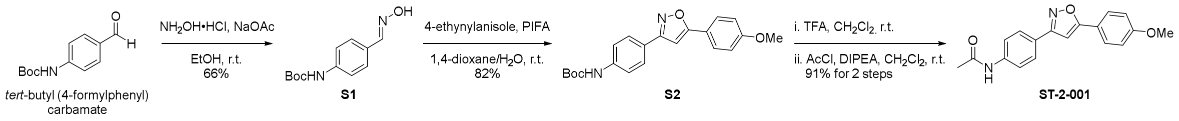

*1.1. tert-Butyl (E)-(4-((hydroxyimino)methyl)phenyl)carbamate (**S1**)*

To a solution of *tert*-butyl (4-formylphenyl)carbamate (3.00 g, 13.56 mmol) in ethanol (50 mL) were added sodium acetate (3.34 g, 40.67 mmol) and hydroxylamine hydrochloride (2.83 g, 40.67 mmol) at ambient temperature. After stirring for 3 h, the reaction mixture was quenched with H_2O, extracted with ethyl acetate, and washed with brine. The combined organic layers were dried over $MgSO_4$ and concentrated *in vacuo*. Further purification was accomplished by flash column chromatography on silica gel (EtOAc/*n*-Hexane = 1:4) to afford the carbamate **S1** (2.10 g, 66%) as ivory solid: ^{1}H NMR (800 MHz, DMSO-d_6) δ 10.98 (s, 1H), 9.50 (brs, 1H), 8.02 (s, 1H), 7.47 (s, 4H), 1.47 (s, 9H); ^{13}C NMR (200 MHz, DMSO-d_6) δ 152.6, 147.7, 140.5, 126.9 (2C), 126.8, 118.0 (2C), 79.3, 28.1 (3C).

*1.2. tert-Butyl (4-(5-(4-methoxyphenyl)isoxazol-3-yl)phenyl)carbamate (**S2**)*

To a mixture of carbamate **S1** (750 mg, 3.17 mmol) and 4-ethynylanisole (286 mg, 2.12 mmol) in 1,4-dioxane/H_2O (5:1, 36 mL) was added [bis(trifluoroacetoxy)iodo] benzene (1.82 g, 4.34 mmol) at ambient temperature. After stirring for 3 h, the reaction mixture was quenched with H_2O, extracted with ethyl acetate, and washed with brine. The combined organic layers were dried over $MgSO_4$ and concentrated *in vacuo*. Further purification was accomplished by flash column chromatography on silica gel (EtOAc/*n*-Hexane = 1:3) to afford the isoxazole **S2** (650 mg, 82%) as ivory solid: ^{1}H NMR (800 MHz, DMSO-d_6) δ 9.62 (brs, 1H), 7.84 (d, J = 8.8 Hz, 2H), 7.79 (d, J = 8.7 Hz, 2H), 7.61 (d, J = 8.5 Hz, 2H), 7.37 (s, 1H), 7.12 (d, J = 8.9 Hz, 2H), 3.84 (s, 3H), 1.49 (s, 9H); ^{13}C NMR (200 MHz, DMSO-d_6) δ 169.4, 162.1, 160.8, 152.6, 141.3, 127.2 (2C), 127.1 (2C), 122.2, 119.6, 118.1 (2C), 114.7 (2C), 96.8, 79.4, 55.4, 28.1 (3C).

*1.3. N-(4-(5-(4-Methoxyphenyl)isoxazol-3-yl)phenyl)acetamide (**ST-2-001**)*

To a solution of isoxazole **S2** (310 mg, 0.85 mmol) in dichloromethane (15 mL) was added trifluoroacetic acid (0.65 mL, 8.46 mmol) dropwise at ambient temperature. After stirring for 3 h, the reaction mixture was quenched with saturated $NaHCO_3$ solution, extracted with dichloromethane, and washed with brine. The combined organic layer was dried over $MgSO_4$ and concentrated *in vacuo* to afford a crude aniline, which was used for the next step without further purification.

To a solution of the above aniline in dichloromethane (15 mL) were added *N*,*N*-diisopropylethylamine (0.44 mL, 2.55 mmol) and acetyl chloride (0.09 mL, 1.28 mmol) at 0 °C. After stirring for 3 h at ambient temperature, the reaction mixture was quenched with H_2O,

extracted with dichloromethane, and washed with brine. The combined organic layers were dried over $MgSO_4$ and concentrated *in vacuo*. Further purification was accomplished by flash column chromatography on silica gel (EtOAc/*n*-Hexane = 1:1) to afford the final compound **ST-2-001** (239 mg, 91%) as white solid: ^{1}H NMR (800 MHz, DMSO-d_6) δ 10.16 (s, 1H), 7.84 (d, J = 8.9 Hz, 2H), 7.83 (d, J = 8.9 Hz, 2H), 7.74 (d, J = 8.6 Hz, 2H), 7.38 (s, 1H), 7.12 (d, J = 8.8 Hz, 2H), 3.84 (s, 3H), 2.08 (s, 3H); ^{13}C NMR (200 MHz, DMSO-d_6) δ 169.5, 168.6, 162.1, 160.8, 141.0, 127.2 (2C), 127.1 (2C), 123.1, 119.6, 119.1 (2C), 114.7 (2C), 96.9, 55.4, 24.1; LR-MS (FAB+) *m/z* 309 (M+H$^+$); HR-MS (FAB+) calcd for $C_{18}H_{17}N_2O_3$ (M+H$^+$) 309.1239, found 309.1251.

*2. Synthesis of **ST-3-001***

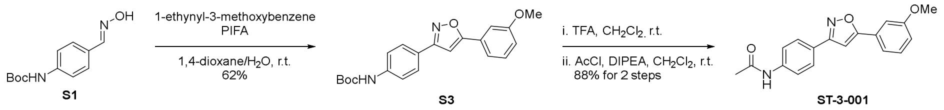

*2.1. tert-Butyl (4-(5-(3-methoxyphenyl)isoxazol-3-yl)phenyl)carbamate (**S3**)*

To a mixture of carbamate **S1** (253 mg, 1.07 mmol) and 1-ethynyl-3-methoxybenzene (93 mg, 0.71 mmol) in 1,4-dioxane/H_2O (5:1, 12 mL) was added [bis(trifluoroacetoxy)iodo] benzene (629 mg, 1.46 mmol) at ambient temperature. After stirring for 3 h, the reaction mixture was quenched with H_2O, extracted with ethyl acetate, and washed with brine. The combined organic layers were dried over $MgSO_4$ and concentrated *in vacuo*. Further purification was accomplished by flash column chromatography on silica gel (EtOAc/*n*-Hexane = 1:3) to afford the isoxazole **S3** (160 mg, 62%) as ivory solid: ^{1}H NMR (800 MHz, DMSO-d_6) δ 9.63 (s, 1H), 7.80 (d, J = 8.7 Hz, 2H), 7.62 (d, J = 8.5 Hz, 2H), 7.56 (s, 1H), 7.49-7.47 (m, 2H), 7.44-7.43 (m, 1H), 7.11-7.08 (m, 1H), 3.86 (s, 3H), 1.50 (s, 9H); ^{13}C NMR (200 MHz, DMSO-d_6) δ 169.3, 162.2, 159.7, 152.6, 141.4, 130.5, 128.1, 127.1 (2C), 122.0, 118.1 (2C), 117.8, 116.3, 110.6, 98.7, 79.4, 55.4, 28.1 (3C).

*2.2. N-(4-(5-(3-Methoxyphenyl)isoxazol-3-yl)phenyl)acetamide (**ST-3-001**)*

To a solution of isoxazole **S3** (100 mg, 0.27 mmol) in dichloromethane (5 mL) was added trifluoroacetic acid (0.21 mL, 2.73 mmol) dropwise at ambient temperature. After stirring for 3 h, the reaction mixture was quenched with saturated $NaHCO_3$ solution, extracted with dichloromethane, and washed with brine. The combined organic layer was dried over $MgSO_4$ and concentrated *in vacuo* to afford a crude aniline, which was used for the next step without further purification.

To a solution of the above aniline in dichloromethane (5 mL) were added *N*,*N*-diisopropylethylamine (0.14 mL, 0.82 mmol) and acetyl chloride (29 μL, 0.41 mmol) at 0 °C. After stirring for 3 h at ambient temperature, the reaction mixture was quenched with H_2O, extracted with dichloromethane, and washed with brine. The combined organic layers were dried over $MgSO_4$ and concentrated *in vacuo*. Further purification was accomplished by flash column chromatography on silica gel (EtOAc/*n*-Hexane = 1:1) to afford amide **ST-3-001** (74 mg, 88%) as white solid: ^{1}H NMR (800 MHz, DMSO-d_6) δ 10.18 (s, 1H), 7.84 (d, J = 8.6 Hz, 2H), 7.75 (d, J = 8.6 Hz, 2H), 7.58 (s, 1H), 7.50-7.47 (m, 2H), 7.45-7.44 (m, 1H), 7.11-7.09 (m, 1H), 3.86 (s, 3H),

2.08 (s, 3H); ^{13}C NMR (200 MHz, DMSO-d_6) δ 169.3, 168.6, 162.2, 159.7, 141.1, 130.5, 128.1, 127.2 (2C), 122.9, 119.1 (2C), 117.8, 116.4, 110.6, 98.7, 55.4, 24.1; LR-MS (FAB+) *m/z* 309 (M+H$^+$); HR-MS (FAB+) calcd for $C_{18}H_{17}N_2O_3$ (M+H$^+$) 309.1239, found 309.1231.

3. Synthesis of ***ST-5-001***

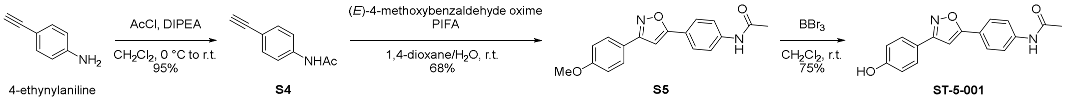

*3.1. N-(4-Ethynylphenyl)acetamide (**S4**)*

To a solution of 4-ethynylaniline (1.00 g, 8.54 mmol) in dichloromethane (85 mL) were *N*,*N*-diisopropylethyl-amine (4.46 mL, 25.6 mmol) and acetyl chloride (0.91 mL, 12.8 mmol) at 0 °C. After stirring for 3 h at ambient temperature, the reaction mixture was quenched with H_2O, extracted with dichloromethane, and washed with brine. The combined organic layers were dried over $MgSO_4$ and concentrated *in vacuo*. Further purification was accomplished by flash column chromatography on silica gel (EtOAc/*n*-Hexane = 1:2) to afford the acetamide **S4** (1.29 g, 95%) as white solid: ^{1}H NMR (800 MHz, $CDCl_3$) δ 7.46 (d, J = 8.6 Hz, 2H), 7.42 (d, J = 8.6 Hz, 2H), 7.27 (brs, 1H), 3.02 (s, 1H), 2.16 (s, 3H); ^{13}C NMR (200 MHz, $CDCl_3$) δ 168.2, 138.3, 132.9 (2C), 119.3 (2C), 117.7, 83.3, 76.8, 24.7.

*3.2. N-(4-(3-(4-Methoxyphenyl)isoxazol-5-yl)phenyl)acetamide (**S5**)*

To a mixture of (*E*)-4-methoxybenzaldehyde oxime (610 mg, 4.04 mmol) and acetamide **S4** (428 mg, 2.69 mmol) in 1,4-dioxane/H_2O (5:1, 12 mL) was added [bis(trifluoroacetoxy)iodo] benzene (2.37 g, 5.51 mmol) at ambient temperature. After stirring for 3 h, the reaction mixture was quenched with H_2O, extracted with ethyl acetate, and washed with brine. The combined organic layers were dried over $MgSO_4$, and concentrated *in vacuo*. Further purification was accomplished by flash column chromatography on silica gel (EtOAc/*n*-Hexane = 1:3) to afford the isoxazole **S5** (564 mg, 68%) as an ivory solid: ^{1}H NMR (800 MHz, DMSO-d_6) δ 10.21 (s, 1H), 7.84 (d, J = 9.0 Hz, 2H), 7.83 (d, J = 9.5 Hz, 2H), 7.76 (d, J = 8.6 Hz, 2H), 7.40 (s, 1H), 7.09 (d, J = 8.8 Hz, 2H), 3.83 (s, 3H), 2.09 (s, 3H); ^{13}C NMR (200 MHz, DMSO-d_6) δ 169.3, 168.7, 162.1, 160.7, 141.2, 128.0 (2C), 126.3 (2C), 121.5, 121.0, 119.1 (2C), 114.5 (2C), 97.2, 55.3, 24.1.

*3.3. N-(4-(3-(4-hydroxyphenyl)isoxazol-5-yl)phenyl)acetamide (**ST-5-001**)*

To a solution of isoxazole **S5** (55 mg, 0.18 mmol) in dichloromethane (2 mL) was added boron tribromide (34 µL, 0.36 mmol) at ambient temperature. After stirring for 12 h, the reaction mixture was quenched with H_2O, extracted with dichloromethane, and washed with brine. The combined organic layers were dried over $MgSO_4$ and concentrated *in vacuo*. Further purification was accomplished by flash column chromatography on silica gel (EtOAc/*n*-Hexane = 1:1) to afford amide **ST-5-001** (39 mg, 75%) as white solid: ^{1}H NMR (800 MHz, DMSO-d_6) δ 10.21 (s, 1H), 9.95 (brs, 1H), 7.82 (d, J = 8.7 Hz, 2H), 7.75 (d, J = 8.7 Hz, 2H), 7.72 (d, J = 8.6 Hz, 2H), 7.33 (s, 1H), 6.89 (d, J = 8.6 Hz, 2H), 2.08 (s, 3H); ^{13}C NMR (200 MHz, DMSO-d_6) δ 169.1, 168.7, 162.3,

159.2, 141.1, 128.1 (2C), 126.3 (2C), 121.6, 119.4, 119.1 (2C), 115.8 (2C), 97.1, 24.1; LR-MS (FAB+) *m/z* 295 ($M+H^+$); HR-MS (FAB+) calcd for $C_{17}H_{15}N_2O_3$ ($M+H^+$) 295.1083, found 295.1089.

4. Synthesis of **ST-4-005**

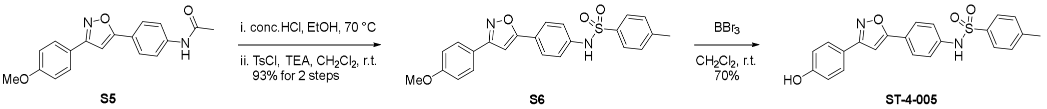

4.1. N-(4-(3-(4-Methoxyphenyl)isoxazol-5-yl)phenyl)-4-methylbenzenesulfonamide (***S6***)

To a solution of isoxazole **S5** (35 mg, 0.11 mmol) in ethanol (2 mL) was added *conc.* hydrogen chloride (0.2 mL) dropwise at ambient temperature. After stirring for 3 h at 70 °C, the reaction mixture was cooled to room temperature, quenched with saturated $NaHCO_3$ solution, and extracted with chloroform/isopropyl alcohol (4:1) solution. The combined organic layer was dried over $MgSO_4$ and concentrated *in vacuo* to afford a crude aniline, which was used for the next step without further purification.

To a solution of the above aniline in dichloromethane (2 mL) were added triethylamine (47 µL, 0.34 mmol) and *p*-toluenesulfonyl chloride (32 mg, 0.17 mmol) at ambient temperature. After stirring for 3 h, the reaction mixture was quenched with H_2O, extracted with dichloromethane, and washed with brine. The combined organic layers were dried over $MgSO_4$, and concentrated *in vacuo*. Further purification was accomplished by flash column chromatography on silica gel (EtOAc/*n*-Hexane = 1:1) to afford the sulfonamide **S6** (44 mg, 93%) as white solid: ^{1}H NMR (800 MHz, DMSO-d_6) δ 10.64 (s, 1H), 7.81 (d, J = 8.8 Hz, 2H), 7.74 (d, J = 8.2 Hz, 2H), 7.70 (d, J = 8.3 Hz, 2H), 7.36 (d, J = 7.8 Hz, 1H), 7.35 (s, 1H), 7.23 (d, J = 7.9 Hz, 2H), 7.08 (d, J = 8.9 Hz, 2H), 3.82 (s, 3H), 2.33 (s, 3H); ^{13}C NMR (200 MHz, DMSO-d_6) δ 162.1, 160.7 (2C), 129.7 (2C), 128.0 (3C), 126.7 (3C), 126.7 (2C), 120.9 (2C), 119.4 (3C), 114.5 (3C), 55.3, 20.9.

4.2. N-(4-(3-(4-Hydroxyphenyl)isoxazol-5-yl)phenyl)-4-methylbenzenesulfonamide (***ST-4-005***)

To a solution of sulfonamide **S6** (40 mg, 0.10 mmol) in dichloromethane (1 mL) was added boron tribromide (18 µL, 0.19 mmol) at ambient temperature. After stirring for 12 h, the reaction mixture was quenched with H_2O, extracted with dichloromethane, and washed with brine. The combined organic layers were dried over $MgSO_4$ and concentrated *in vacuo*. Further purification was accomplished by flash column chromatography on silica gel (EtOAc/*n*-Hexane = 1:1) to afford amide **ST-4-005** (27 mg, 70%) as white solid: ^{1}H NMR (800 MHz, DMSO-d_6) δ 10.62 (s, 1H), 9.92 (s, 1H), 7.74 (d, J = 8.7 Hz, 2H), 7.70 (d, J = 7.8 Hz, 2H), 7.69 (d, J = 8.2 Hz, 2H), 7.37 (d, J = 8.2 Hz, 2H), 7.30 (s, 1H), 7.25 (d, J = 8.7 Hz, 2H), 6.88 (d, J = 8.7 Hz, 2H), 2.33 (s, 3H); ^{13}C NMR (200 MHz, DMSO-d_6) δ 168.7, 162.3, 159.2, 143.6, 139.7, 136.4, 129.8 (2C), 128.1 (2C), 126.7 (2C), 126.7 (2C), 122.3, 119.4 (2C), 119.3, 115.8 (2C), 97.4, 20.9; LR-MS (FAB+) *m/z* 357 ($M+H^+$); HR-MS (FAB+) calcd for $C_{22}H_{19}N_2O_4S$ ($M+H^+$) 407.1066, found 407.1065.

*5. Synthesis of **ST-5-002***

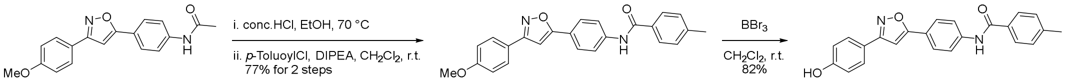

*5.1. N-(4-(3-(4-Methoxyphenyl)isoxazol-5-yl)phenyl)-4-methylbenzamide (**S7**)*

To a solution of isoxazole **S5** (60 mg, 0.19 mmol) in ethanol (2 mL) was added *conc.* hydrogen chloride (0.2 mL) dropwise at ambient temperature. After stirring for 3 h at 70 °C, the reaction mixture was cooled to room temperature, quenched with saturated $NaHCO_3$ solution, and extracted with chloroform/isopropyl alcohol (4:1) solution. The combined organic layer was dried over $MgSO_4$ and concentrated *in vacuo* to afford a crude aniline, which was used for the next step without further purification.

To a solution of the above aniline in dichloromethane (2 mL) were added *N*,*N*-diisopropylethylamine (0.10 mL, 0.58 mmol) and *p*-toluoyl chloride (39 µL, 0.29 mmol) at ambient temperature. After stirring for 3 h, the reaction mixture was quenched with H_2O, extracted with dichloromethane, and washed with brine. The combined organic layers were dried over $MgSO_4$ and concentrated *in vacuo*. Further purification was accomplished by flash column chromatography on silica gel (EtOAc/*n*-Hexane = 1:1) to afford the *p*-toluamide **S7** (97 mg, 77%) as white solid: ^{1}H NMR (800 MHz, DMSO-d_6) δ 10.42 (s, 1H), 7.99 (d, *J* = 8.7 Hz, 2H), 7.90 (d, *J* = 8.1 Hz, 2H), 7.89 (d, *J* = 8.7 Hz, 2H), 7.85 (d, *J* = 8.7 Hz, 2H), 7.45 (s, 1H), 7.36 (d, *J* = 7.9 Hz, 2H), 7.10 (d, *J* = 8.8 Hz, 2H), 3.84 (s, 3H), 2.40 (s, 3H); ^{13}C NMR (200 MHz, DMSO-d_6) δ 169.3, 165.6, 162.1, 160.7, 141.9, 141.2, 131.8, 129.0 (2C), 128.0 (2C), 127.8 (2C), 126.1 (2C), 122.0, 121.0, 120.4 (2C), 114.5 (2C), 97.4, 55.3, 21.0.

*5.2. N-(4-(3-(4-Hydroxyphenyl)isoxazol-5-yl)phenyl)-4-methylbenzamide (**ST-5-002**)*

To a solution of *p*-toluamide **S7** (70 mg, 0.18 mmol) in dichloromethane (2 mL) was added boron tribromide (35 µL, 0.36 mmol) at ambient temperature. After stirring for 12 h, the reaction mixture was quenched with H_2O, extracted with dichloromethane, and washed with brine. The combined organic layers were dried over $MgSO_4$ and concentrated *in vacuo*. Further purification was accomplished by flash column chromatography on silica gel (EtOAc/*n*-Hexane = 1:1) to afford amide **ST-5-002** (55 mg, 82%) as white solid: ^{1}H NMR (800 MHz, DMSO-d_6) δ 10.42 (s, 1H), 9.96 (brs, 1H), 7.99 (d, *J* = 8.7 Hz, 2H), 7.90 (d, *J* = 8.1 Hz, 2H), 7.88 (d, *J* = 8.7 Hz, 2H), 7.73 (d, *J* = 8.6 Hz, 2H), 7.38 (s, 1H), 7.36 (d, *J* = 7.9 Hz, 2H), 6.90 (d, *J* = 8.6 Hz, 2H), 2.40 (s, 3H); ^{13}C NMR (200 MHz, DMSO-d_6) δ 169.1, 165.6, 162.3, 159.3, 141.9, 141.1, 131.8, 129.0 (2C), 128.1 (2C), 127.8 (2C), 126.1 (2C), 122.0, 120.4 (2C), 119.4, 115.8 (2C), 97.3, 21.0; LR-MS (FAB+) *m/z* 371 (M+H$^+$); HR-MS (FAB+) calcd for $C_{23}H_{19}N_2O_3$ (M+H$^+$) 371.1396, found 371.1389.

II. ¹H and ¹³C NMR spectra of the synthesized intermediates and products

Chemical Information, CI 1a. ¹H and ¹³C NMR spectra of **S1**

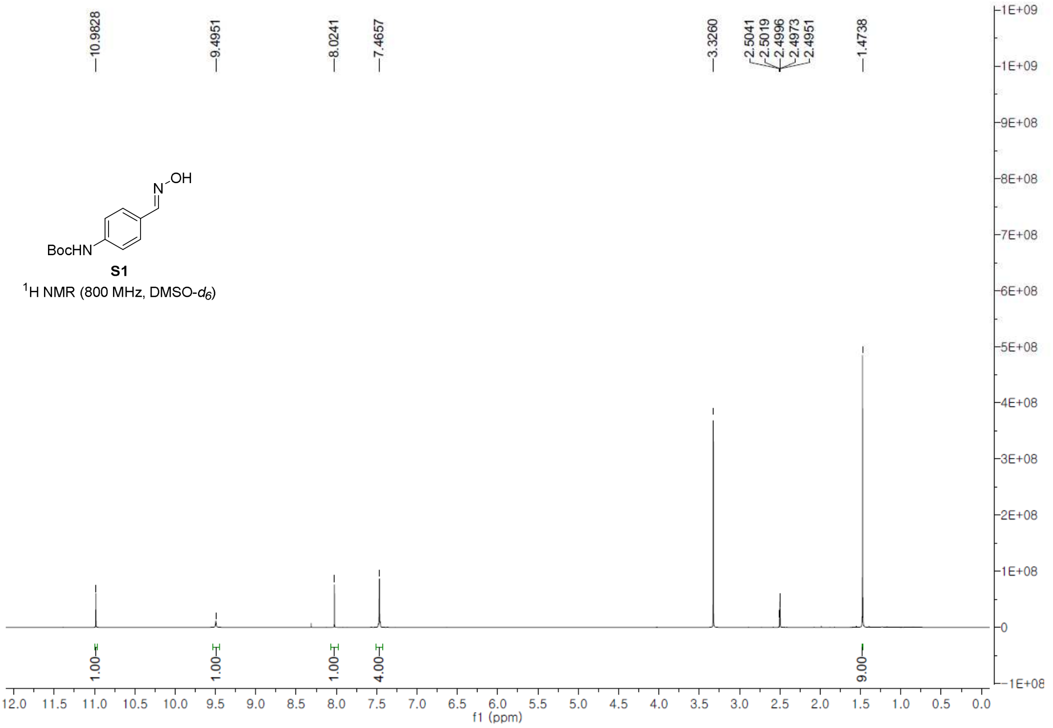

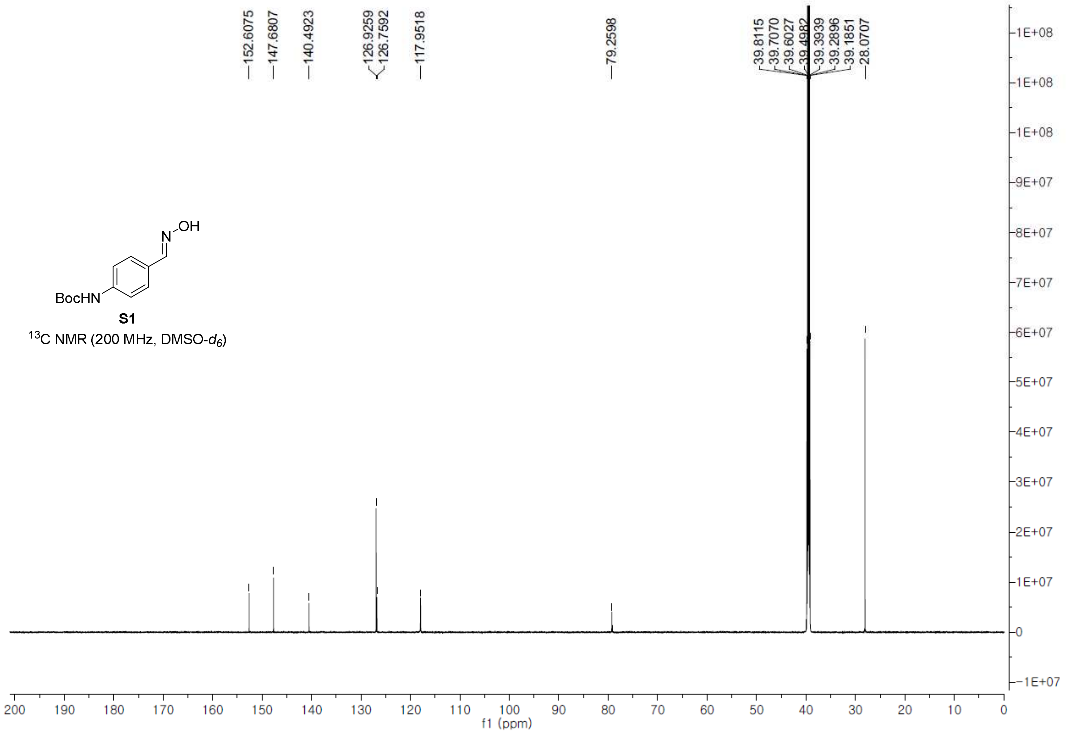

CI 1b. ^{1}H and ^{13}C NMR spectra of **S2**

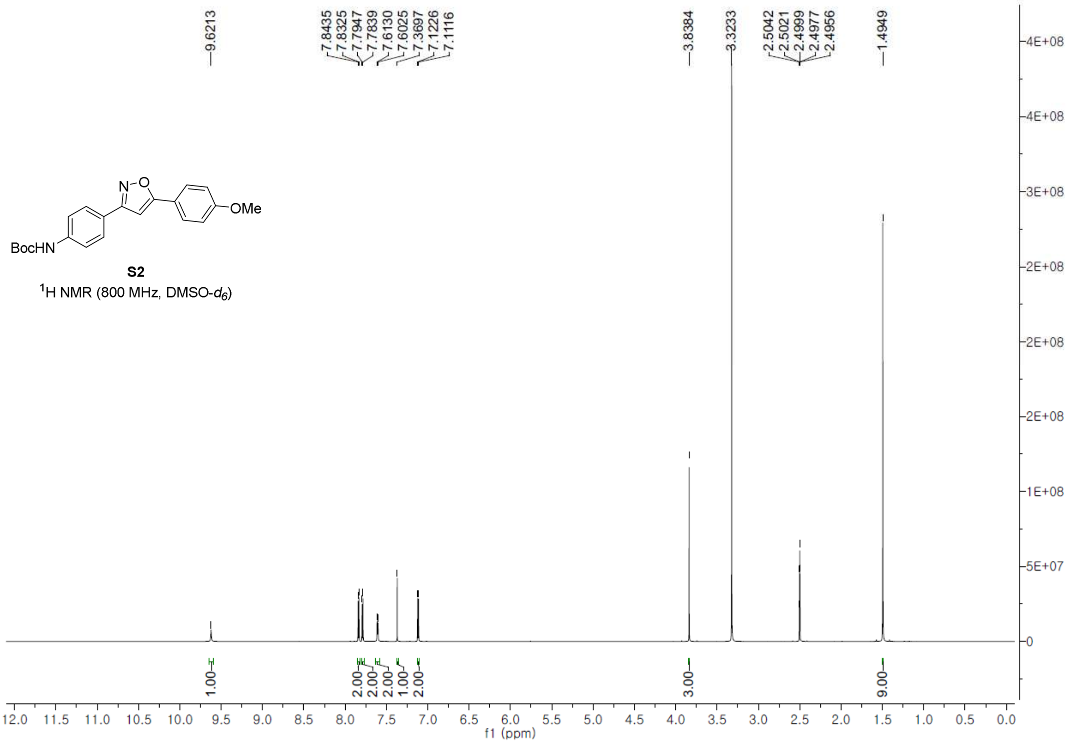

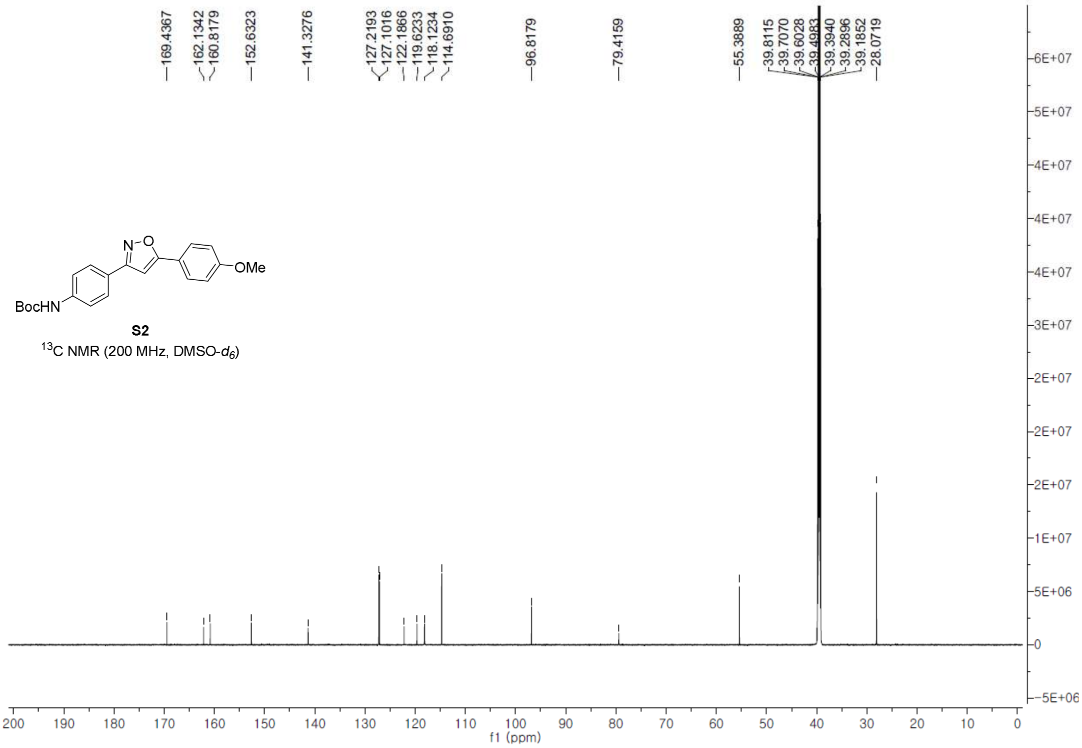

CI 1c. ^{1}H and ^{13}C NMR spectra of **ST-2-001**

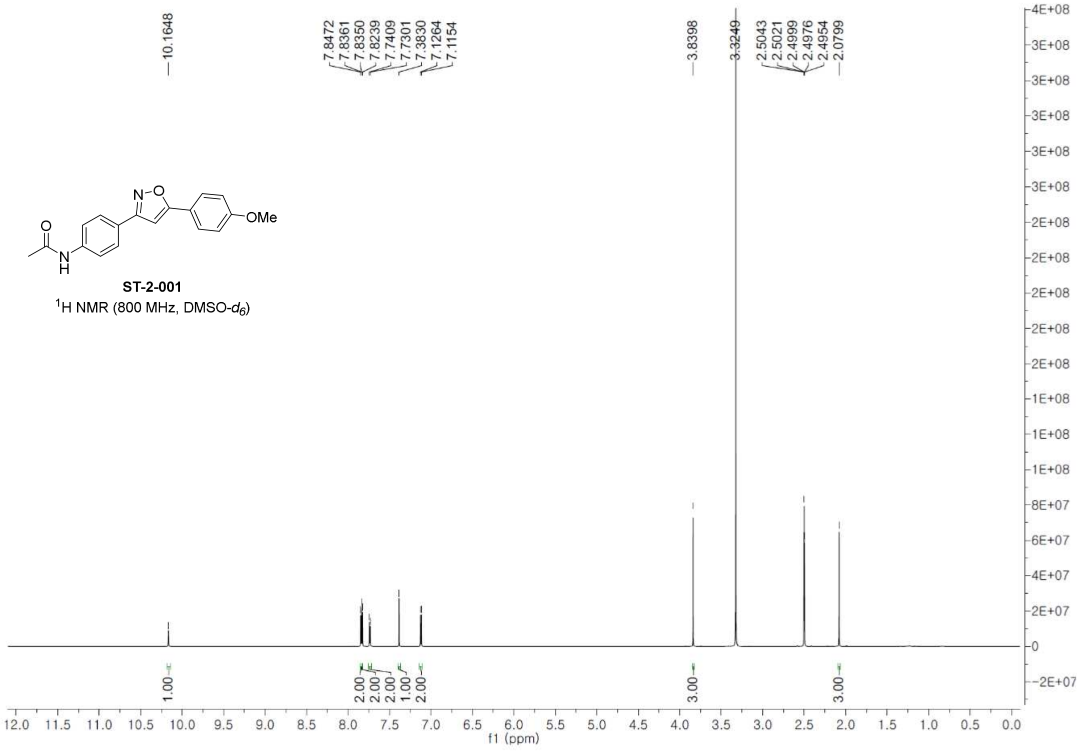

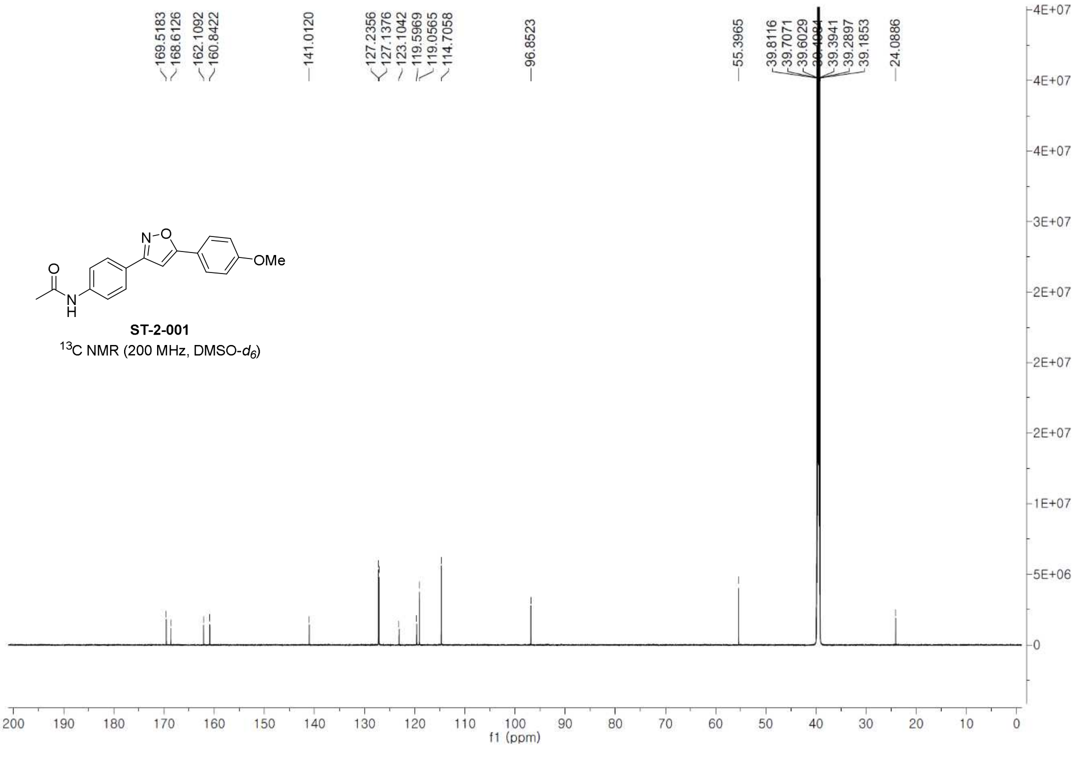

CI 1d. ^{1}H and ^{13}C NMR spectra of **S3**

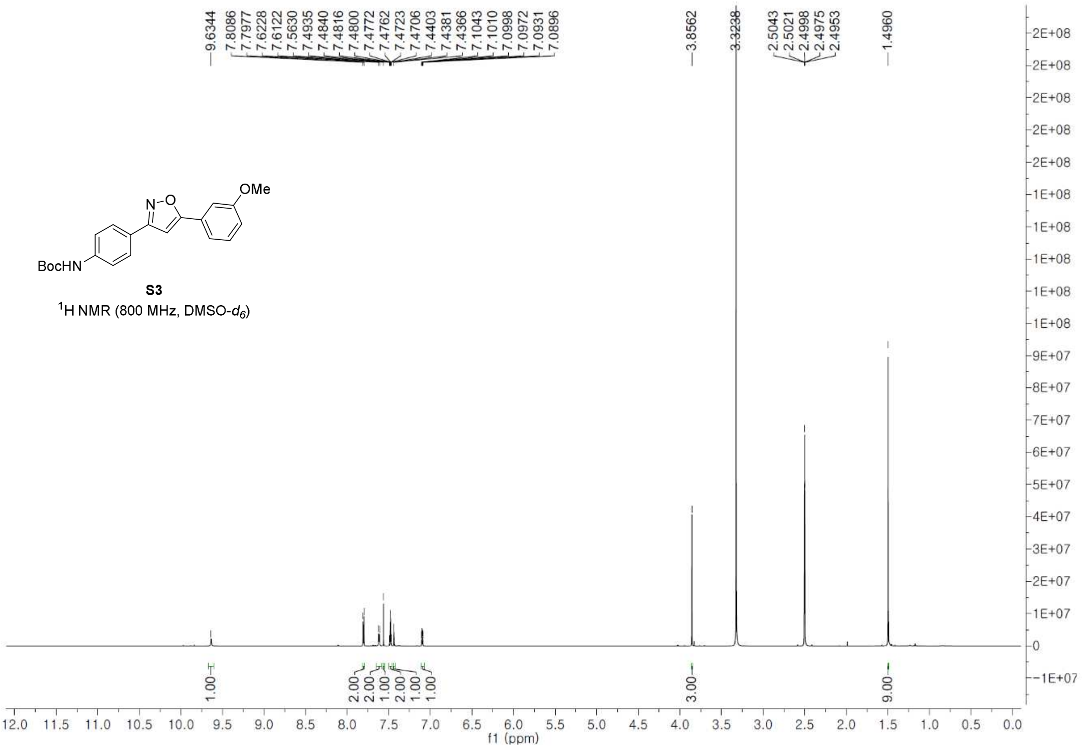

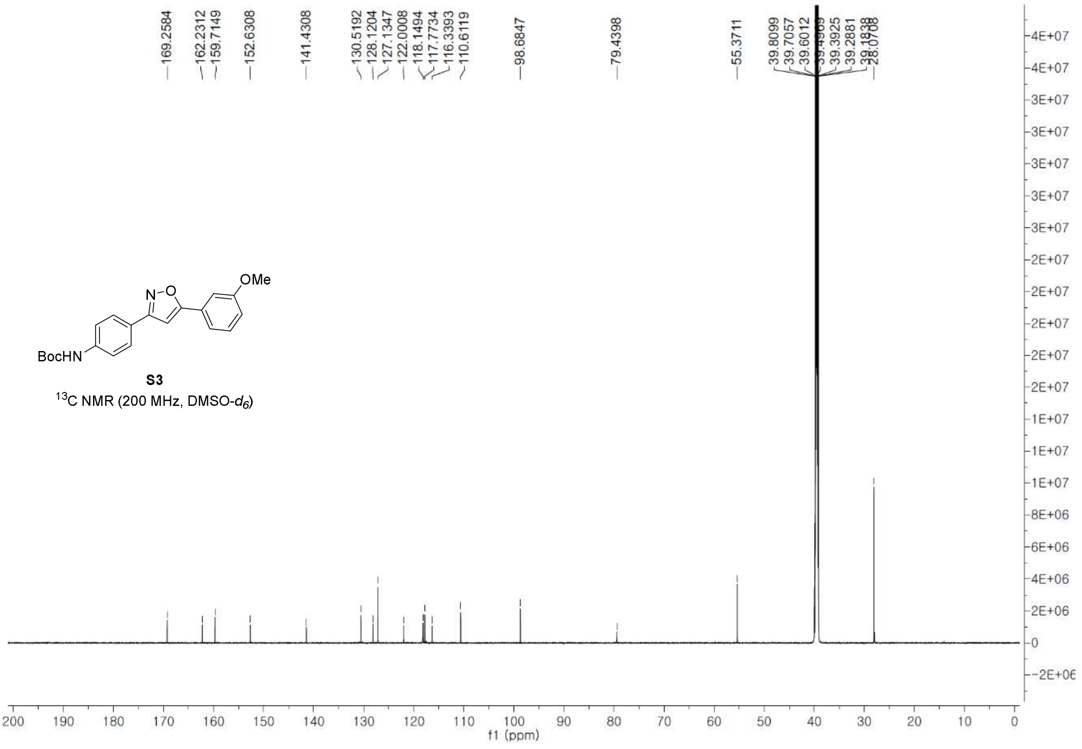

CI 1e. ^{1}H and ^{13}C NMR spectra of **ST-3-001**

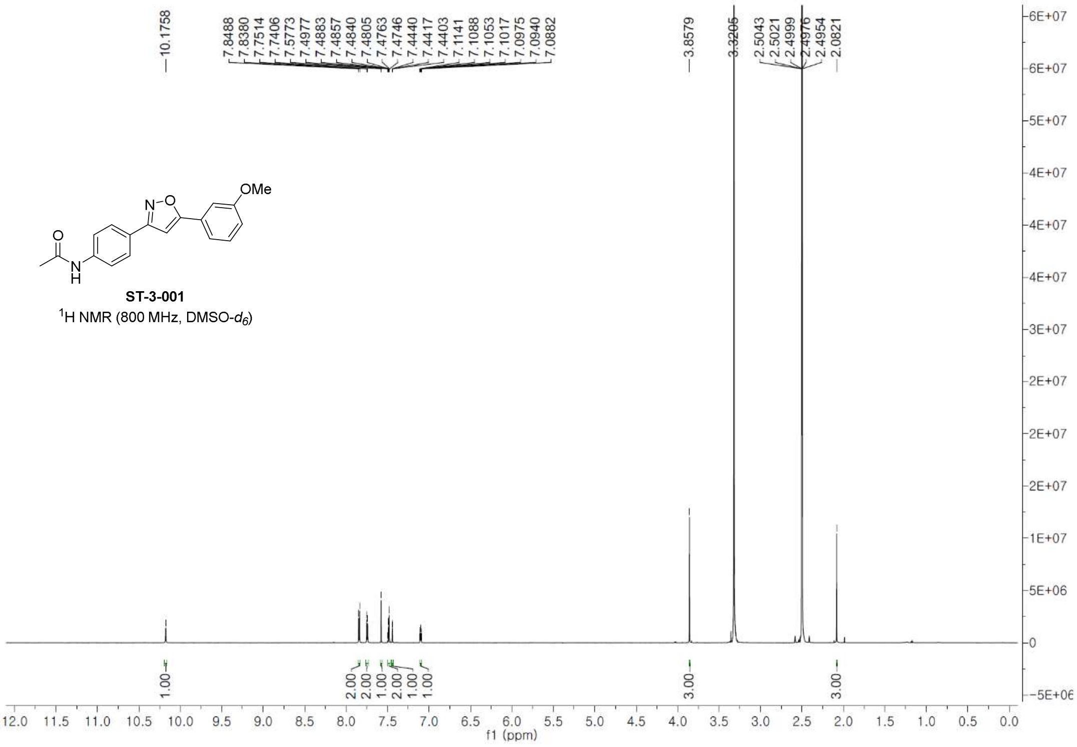

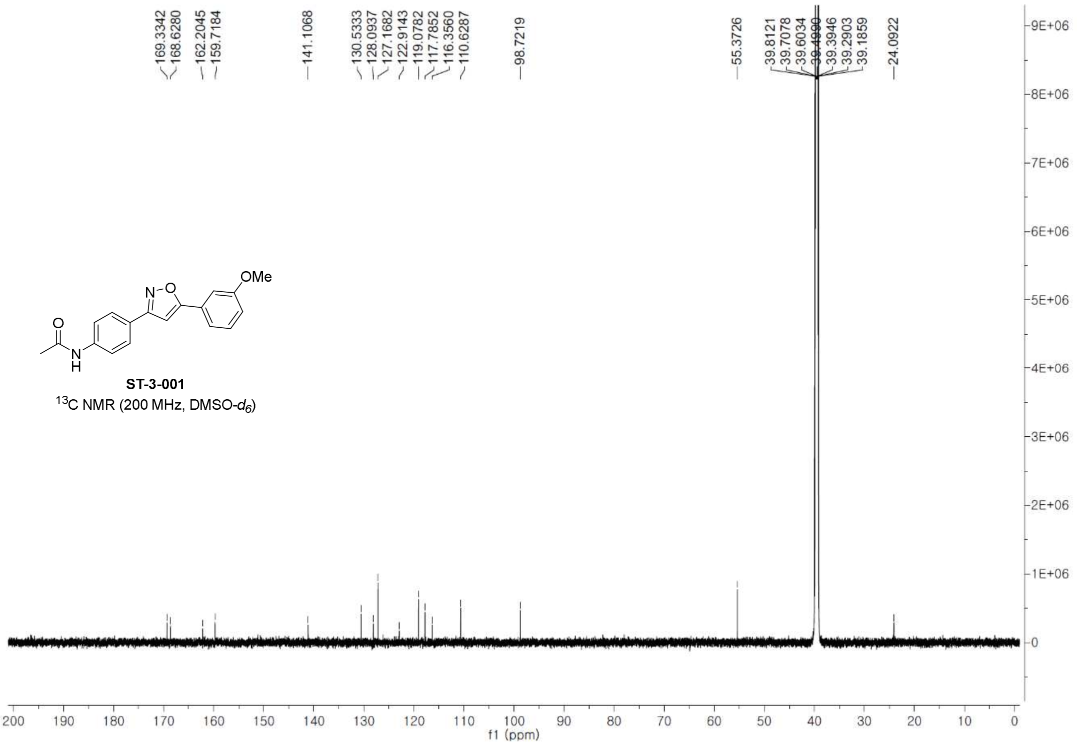

CI 1f. ^{1}H and ^{13}C NMR spectra of **S4**

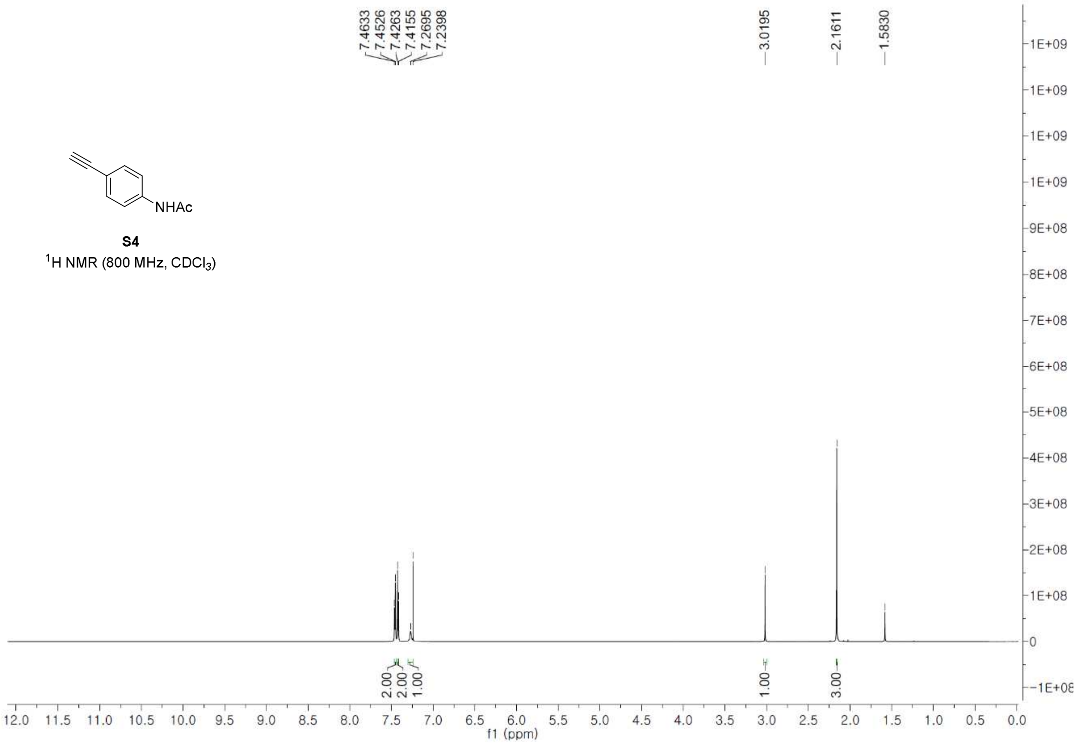

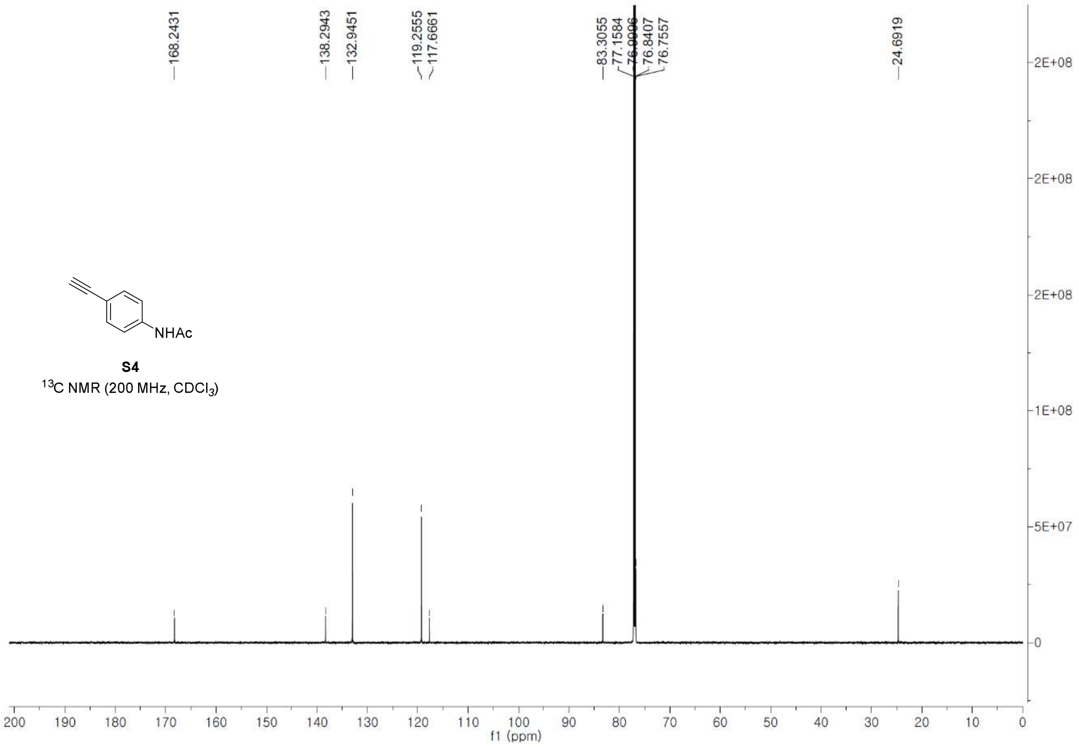

CI 1g. ^{1}H and ^{13}C NMR spectra of **S5**

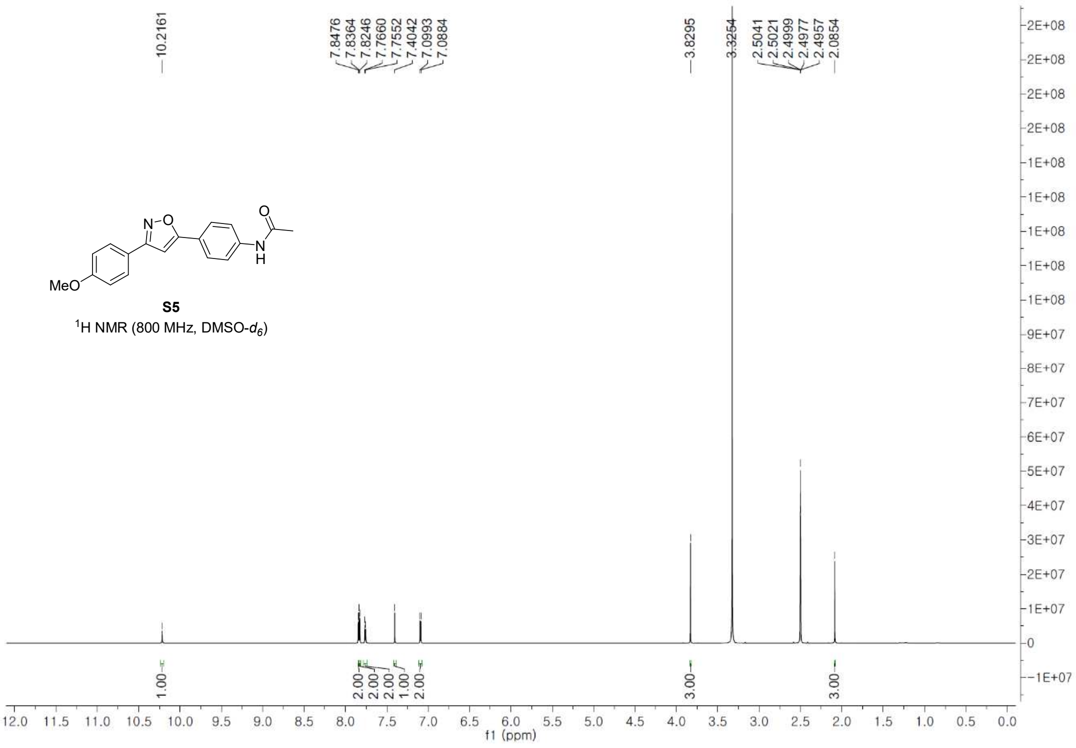

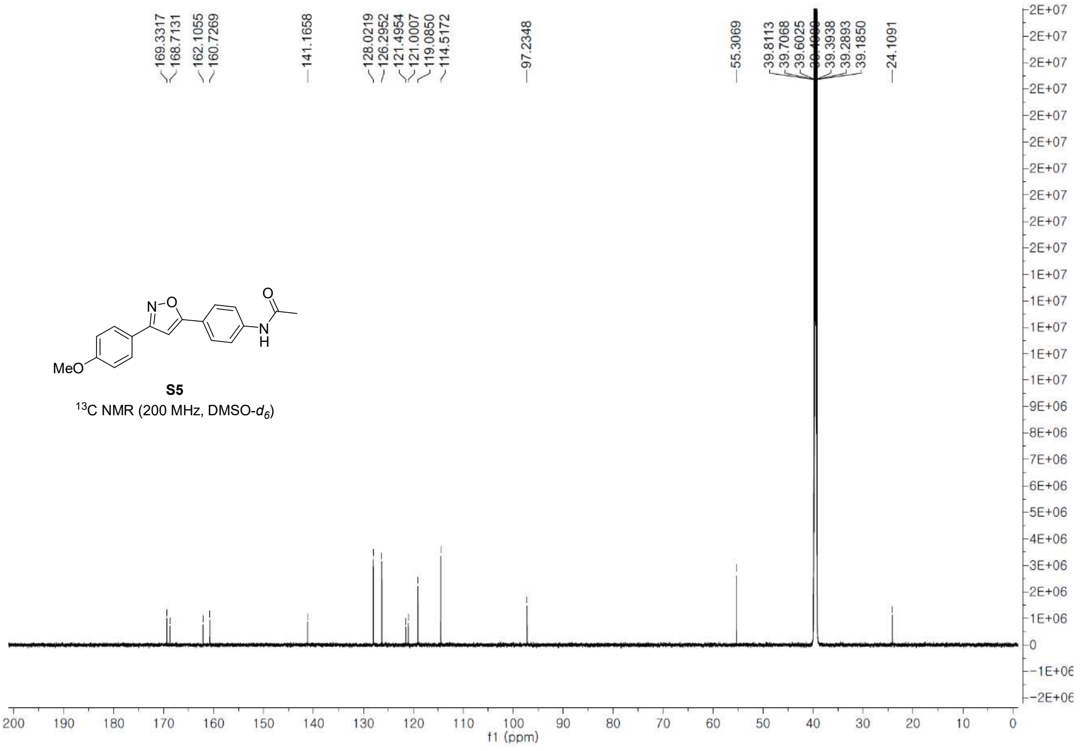

CI 1h. ^{1}H and ^{13}C NMR spectra of **ST-5-001**

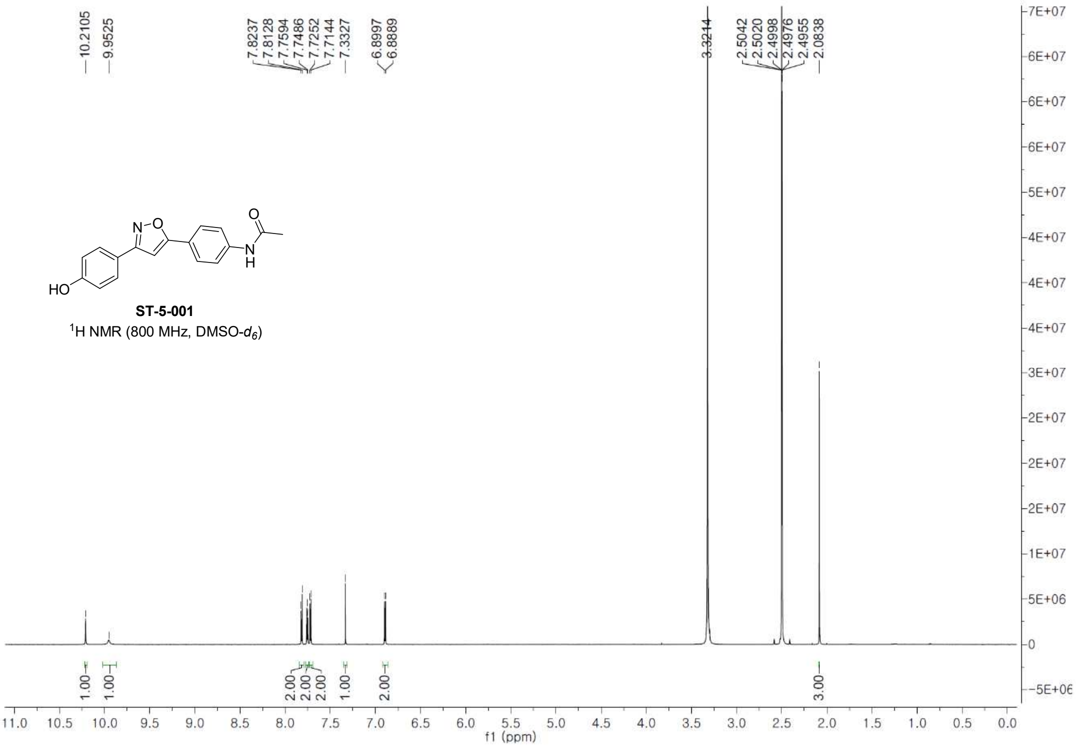

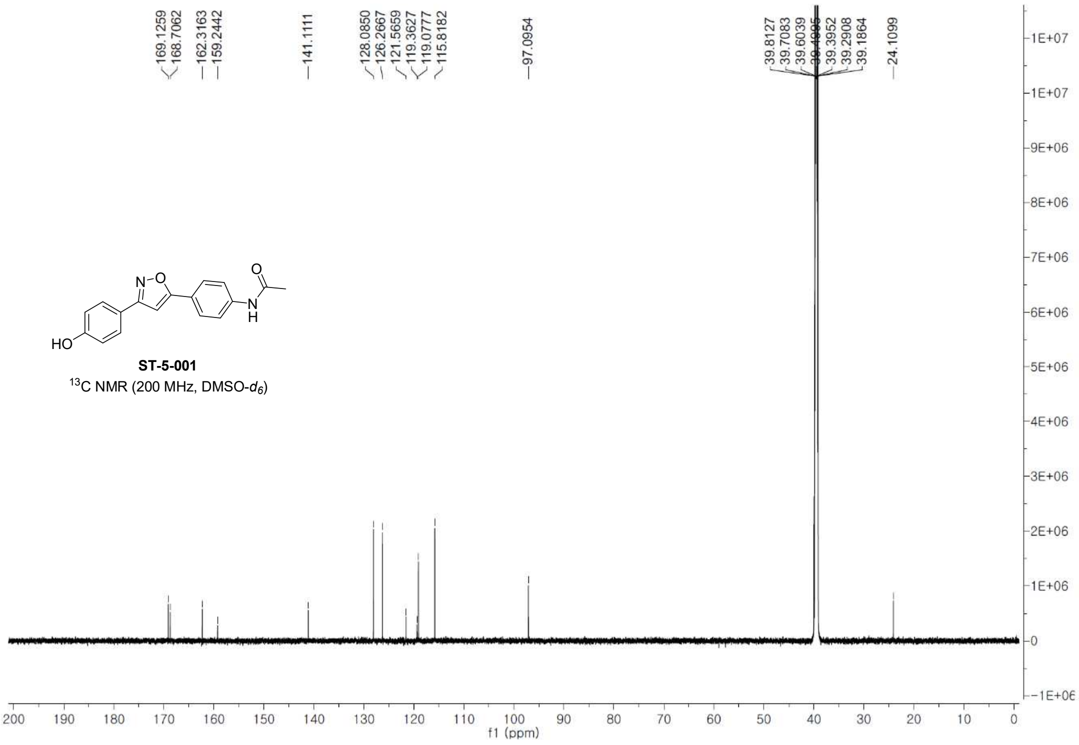

CI 1i. ^{1}H and ^{13}C NMR spectra of **S6**

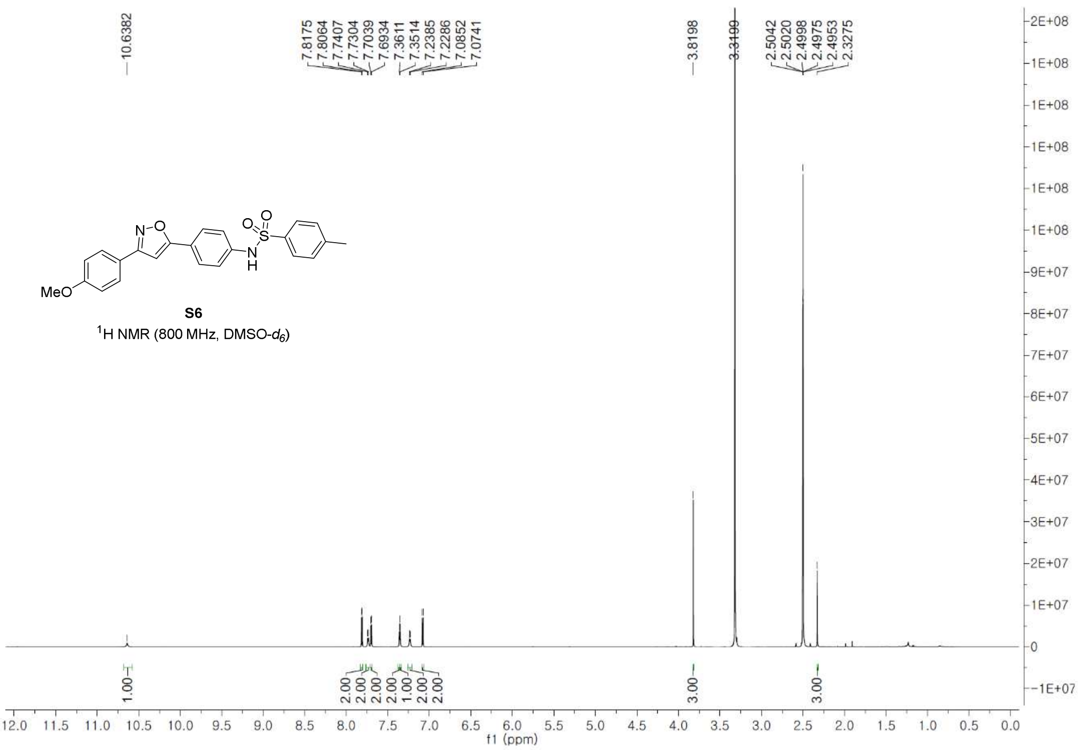

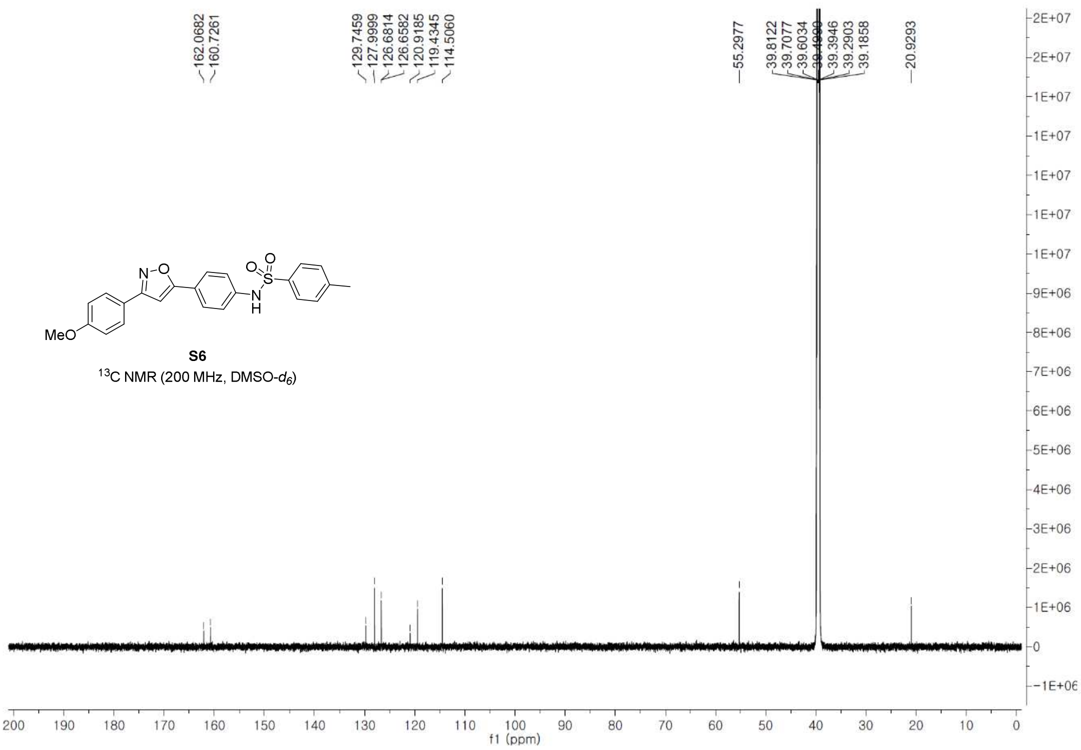

CI 1j. ^{1}H and ^{13}C NMR spectra of **ST-4-005**

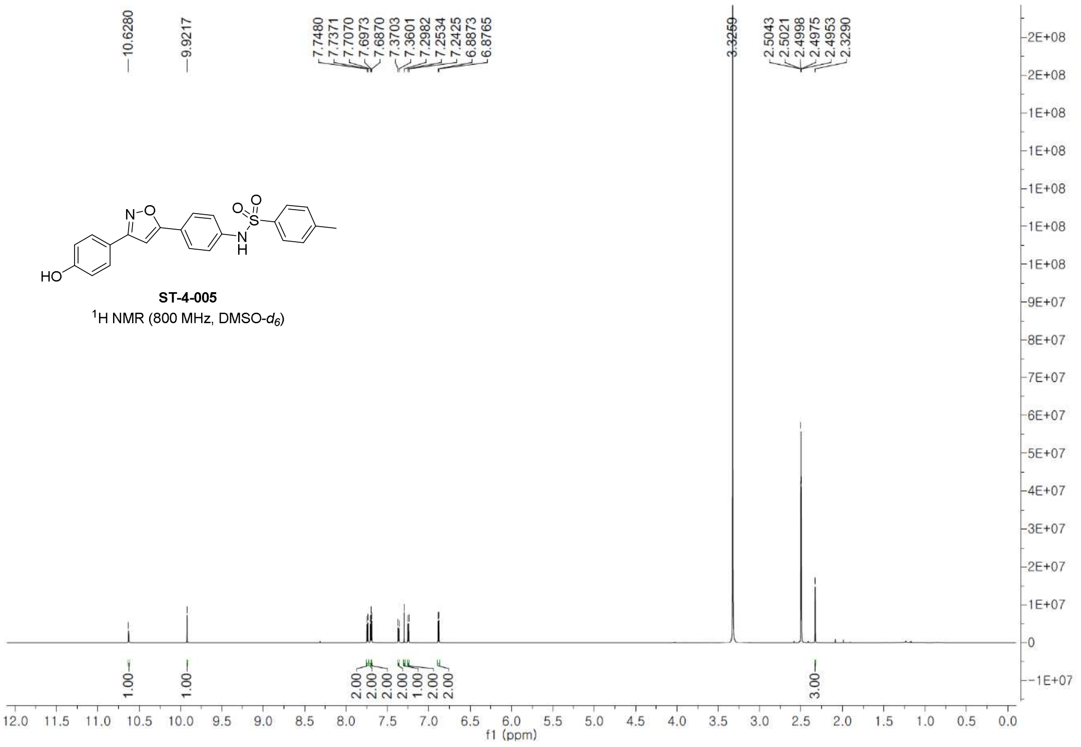

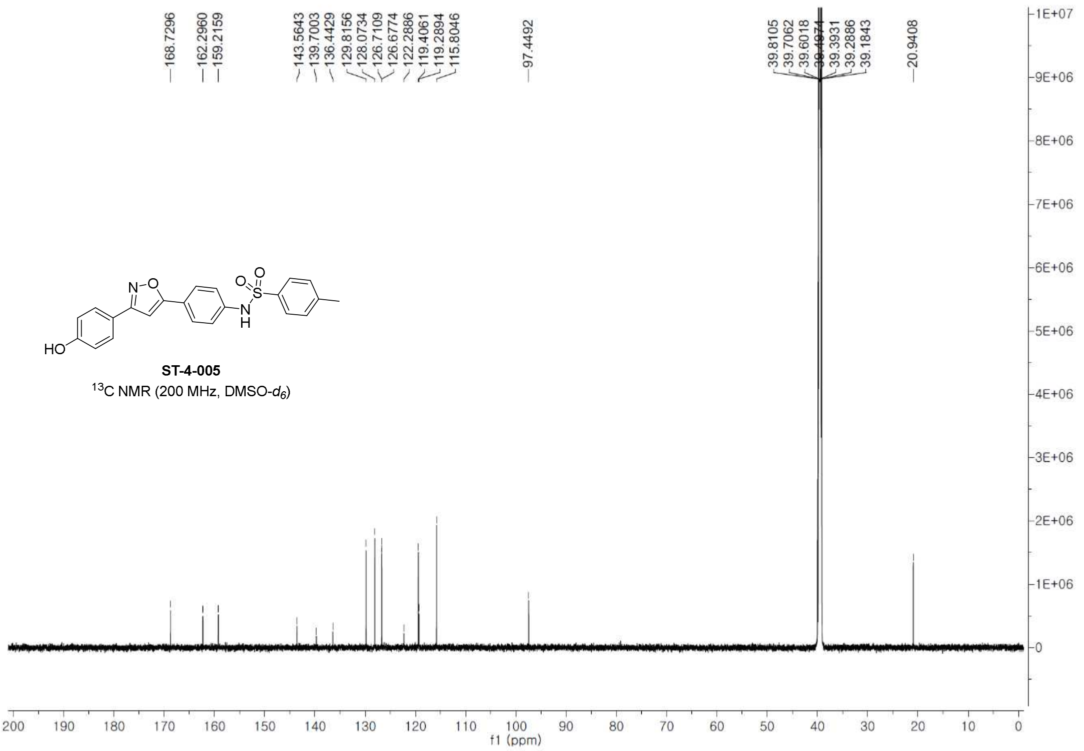

CI 1k. ^{1}H and ^{13}C NMR spectra of **S7**

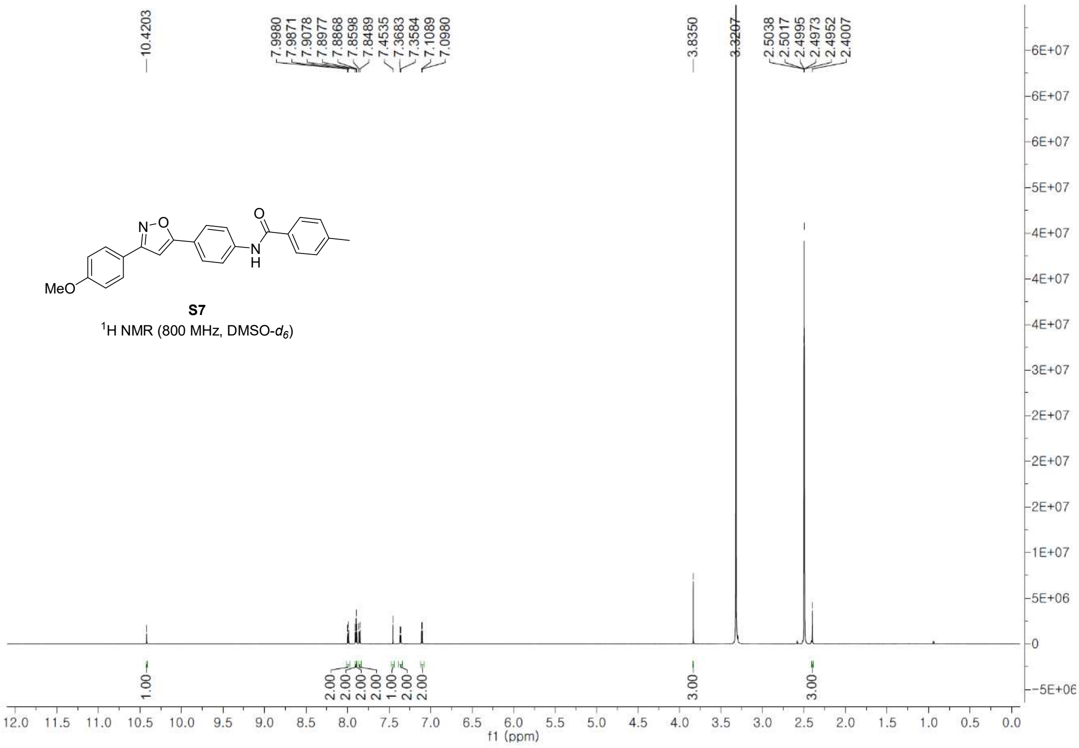

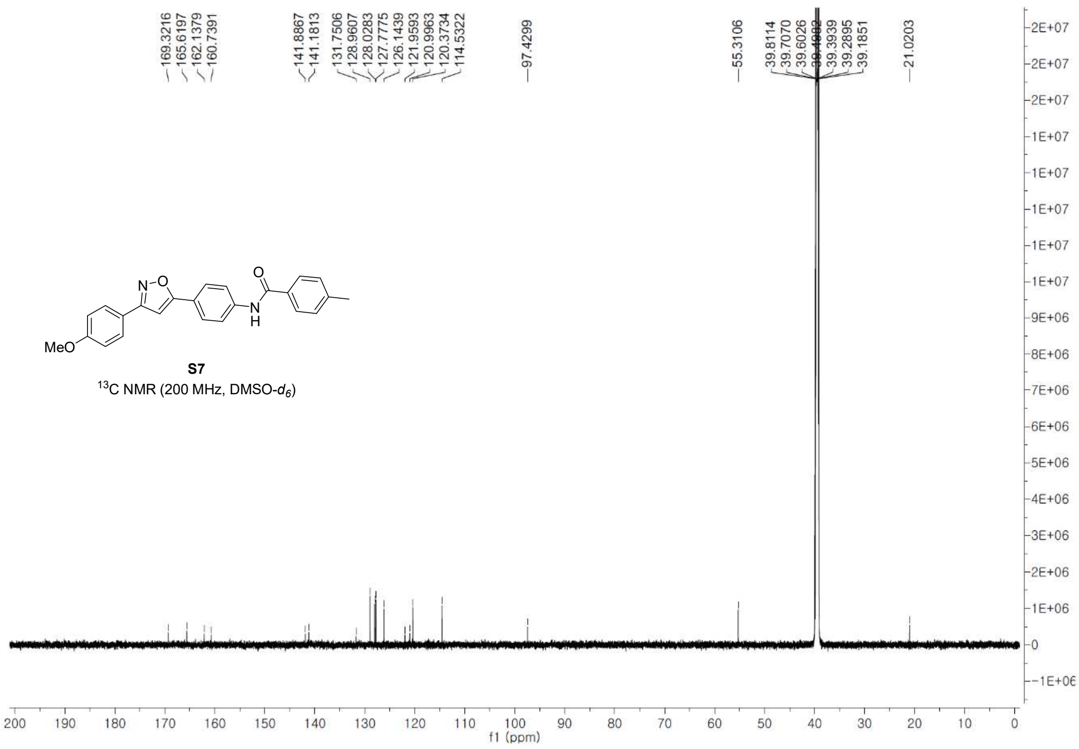

CI 1l. ^{1}H and ^{13}C NMR spectra of **ST-5-002**

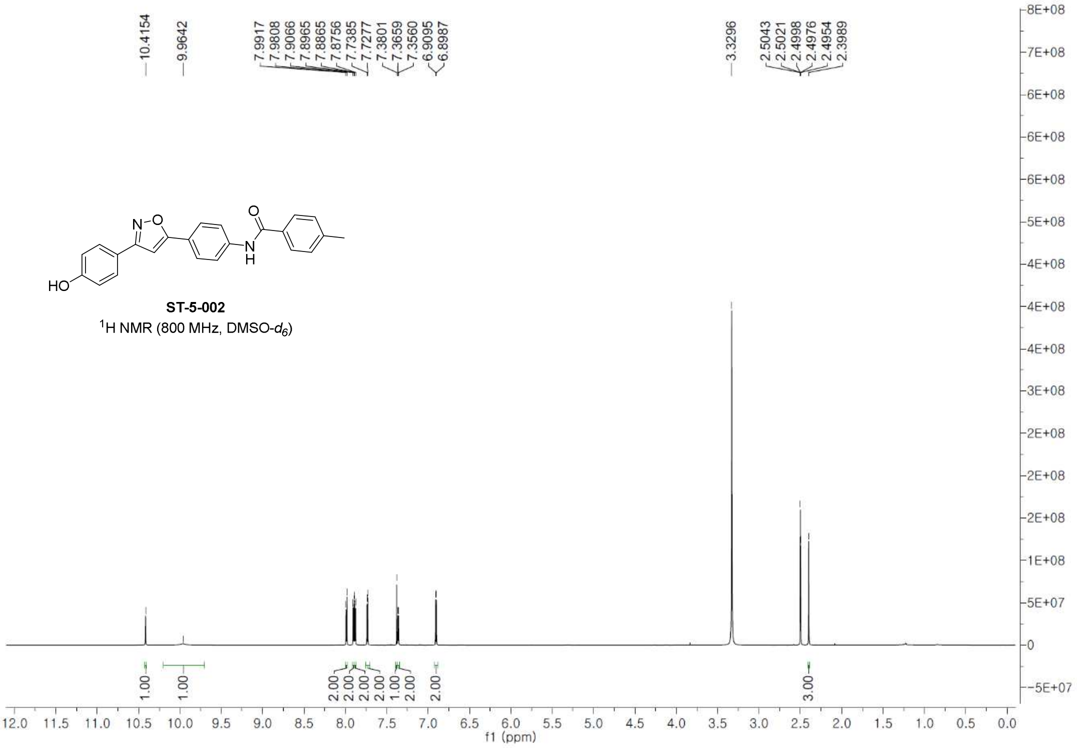

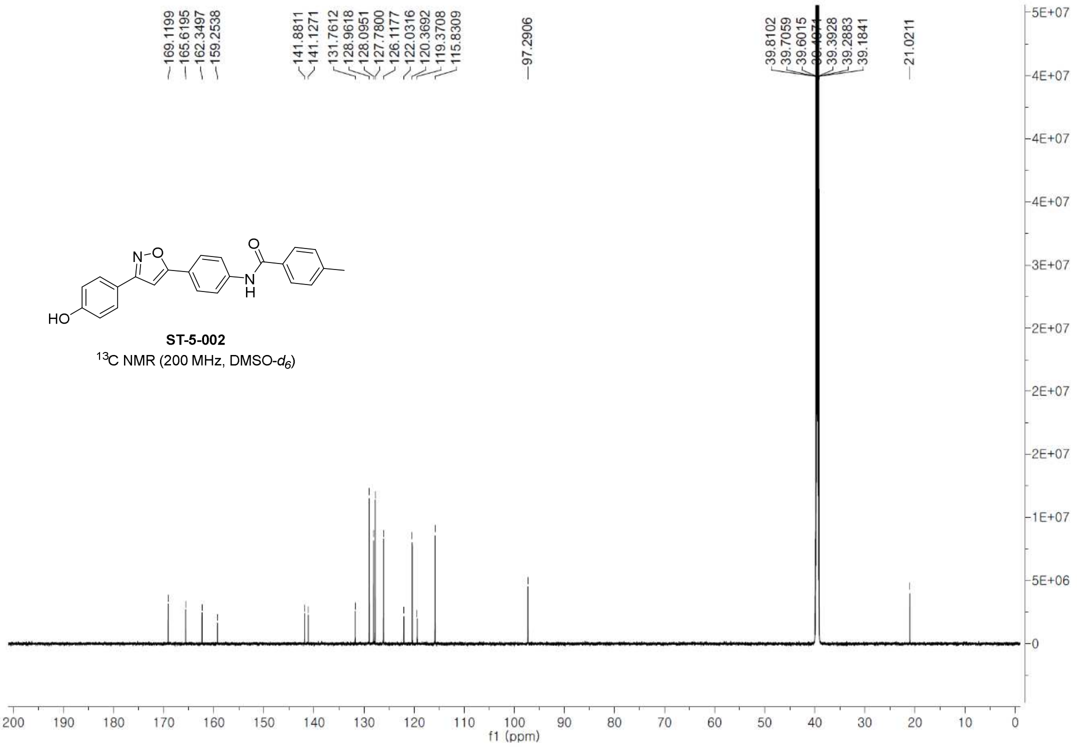

III. HRMS data of final compounds

CI 2a. HRMS data of **ST-2-001**

Data : F44656 Date : 27-Mar-2024 17:13
Instrument : MStation
Sample : 4NH2Ac
Note : m-NBA
Inlet : Direct Ion Mode : FAB+
RT : 0.00 min Scan# : (1,141)
Elements : C 50/0, H 100/0, N 10/0, O 10/0
Mass Tolerance : 10ppm, 5mmu if m/z < 500, 10mmu if m/z > 1000
Unsaturation (U.S.) : -0.5 - 20.0

	Observed m/z	Int%	Err[ppm / mmu]	U.S.	Composition
1	309.1251	100.00	-4.8 / -1.5	16.0	C21 H15 N3
2			-9.2 / -2.8	15.5	C23 H17 O
3			+12.5 / +3.9	12.5	C14 H13 N8 O
4			+8.2 / +2.5	12.0	C16 H15 N5 O2
5			+3.8 / +1.2	11.5	C18 H17 N2 O3
6			-15.2 / -4.7	8.0	C10 H15 N9 O3
7			+12.5 / +3.9	7.0	C15 H19 N O6
8			-6.5 / -2.0	3.5	C7 H17 N8 O6
9			-10.8 / -3.3	3.0	C9 H19 N5 O7
10			-15.2 / -4.7	2.5	C11 H21 N2 O8
11			+6.5 / +2.0	-0.5	C2 H17 N10 O8

CI 2b. HRMS data of **ST-3-001**

Data : F44654 Date : 27-Mar-2024 15:39
Instrument : MStation
Sample : #138
Note : m-NBA
Inlet : Direct Ion Mode : FAB+
RT : 5.56 min Scan# : (69,89)
Elements : C 50/0, H 100/0, N 10/0, O 10/0
Mass Tolerance : 10ppm, 5mmu if m/z < 500, 10mmu if m/z > 1000
Unsaturation (U.S.) : -0.5 - 15.0

	Observed m/z	Int%	Err[ppm / mmu]	U.S.	Composition
1	309.1231	77.35	+6.0 / +1.9	12.5	C14 H13 N8 O
2			+1.7 / +0.5	12.0	C16 H15 N5 O2
3			-2.6 / -0.8	11.5	C18 H17 N2 O3
4			+14.7 / +4.5	8.0	C11 H15 N7 O4
5			+10.4 / +3.2	7.5	C13 H17 N4 O5
6			+6.0 / +1.9	7.0	C15 H19 N O6
7			-13.0 / -4.0	3.5	C7 H17 N8 O6
8			+0.1 / +0.0	-0.5	C2 H17 N10 O8
9			+14.7 / +4.5	2.5	C12 H21 O9

CI 2c. HRMS data of **ST-5-001**

Data : F43892 Date : 17-Nov-2023 15:00
Instrument : MStation
Sample : 154-P
Note : m-NBA
Inlet : Direct Ion Mode : FAB+
RT : 0.00 min Scan# : (1,177)
Elements : C 50/0, H 100/0, N 10/0, O 10/0
Mass Tolerance : 10ppm, 5mmu if m/z < 500, 10mmu if m/z > 1000
Unsaturation (U.S.) : -0.5 - 20.0

	Observed m/z	Int%	Err[ppm / mmu]	U.S.	Composition
1	295.1089	90.16	-6.9 / -2.0	16.0	C20 H13 N3
2			-11.5 / -3.4	15.5	C22 H15 O
3			+11.2 / +3.3	12.5	C13 H11 N8 O
4			+6.7 / +2.0	12.0	C15 H13 N5 O2
5			+2.1 / +0.6	11.5	C17 H15 N2 O3
6			+15.8 / +4.7	7.5	C12 H15 N4 O5
7			+11.2 / +3.3	7.0	C14 H17 N O6
8			-8.7 / -2.6	3.5	C6 H15 N8 O6
9			-13.2 / -3.9	3.0	C8 H17 N5 O7
10			+5.0 / +1.5	-0.5	C H15 N10 O8

CI 2d. HRMS data of **ST-4-005**

Data : F44652 Date : 27-Mar-2024 14:06
Instrument : MStation
Sample : #152
Note : m-NBA
Inlet : Direct Ion Mode : FAB+
RT : 0.00 min Scan# : (1,78)
Elements : C 50/0, H 100/0, N 5/0, O 10/0, S 1/0
Mass Tolerance : 10ppm, 5mmu if m/z < 500, 10mmu if m/z > 1000
Unsaturation (U.S.) : -0.5 - 20.0

	Observed m/z	Int%	Err[ppm / mmu]	U.S.	Composition
1	407.1065	53.02	+11.4 / +4.7	20.0	C23 H13 N5 O3
2			+8.1 / +3.3	19.5	C25 H15 N2 O4
3			-3.0 / -1.2	11.0	C16 H17 N5 O8
4			-6.3 / -2.6	10.5	C18 H19 N2 O9
5			-6.7 / -2.7	20.0	C25 H17 N3 O S
6			-10.0 / -4.1	19.5	C27 H19 O2 S
7			+3.2 / +1.3	16.0	C20 H17 N5 O3 S
8			-0.1 / -0.1	15.5	C22 H19 N2 O4 S
9			+9.7 / +4.0	11.5	C17 H19 N4 O6 S
10			+6.5 / +2.6	11.0	C19 H21 N O7 S
11			-11.3 / -4.6	7.0	C13 H21 N5 O8 S

CI 2e. HRMS data of **ST-5-002**

Data : F43791 Date : 03-Nov-2023 14:00
Instrument : MStation
Sample : ST-5-002
Note : m-NBA
Inlet : Direct Ion Mode : FAB+
RT : 11.42 min Scan# : (138,178)
Elements : C 50/0, H 100/0, N 10/0, O 10/0
Mass Tolerance : 10ppm, 5mmu if m/z < 500, 10mmu if m/z > 1000
Unsaturation (U.S.) : -0.5 - 20.0

	Observed m/z	Int%	Err[ppm / mmu]	U.S.	Composition
1	371.1389	40.41	-9.0 / -3.3	20.0	C26 H17 N3
2			-12.6 / -4.7	19.5	C28 H19 O
3			+5.4 / +2.0	16.5	C19 H15 N8 O
4			+1.8 / +0.7	16.0	C21 H17 N5 O2
5			-1.8 / -0.7	15.5	C23 H19 N2 O3
6			+12.7 / +4.7	12.0	C16 H17 N7 O4
7			+9.0 / +3.4	11.5	C18 H19 N4 O5
8			+5.4 / +2.0	11.0	C20 H21 N O6
9			-10.4 / -3.9	7.5	C12 H19 N8 O6
10			+0.5 / +0.2	3.5	C7 H19 N10 O8
11			+12.6 / +4.7	6.5	C17 H23 O9
12			-3.2 / -1.2	3.0	C9 H21 N7 O9
13			-6.8 / -2.5	2.5	C11 H23 N4 O10

IV. HPLC purities of final compounds

Purity of final compounds was confirmed by HPLC analysis with SHIMADZU LC-2050C 3D (SHIMADZU, Kyoto, Japan). HPLC chromatograms were obtained under the following conditions; Column, SunFire® C18, 4.6 x 150 mm, 5 µm; detection, UV 254 nm; flow rate 1.0 mL/min; Mobile phase, Acetonitrile (CH_3CN) and Water (H_2O), Isocratic.

Table S1. HPLC purities of final compounds

Compounds	Area% (Major peak, 254 nm)[a]	Mobile phase condition CH_3CN(%)/H_2O(%)
**ST-2-001**	99.4	55/45
**ST-3-001**	99.9	55/45
**ST-5-001**	99.8	40/60
**ST-4-005**	99.3	55/45
**ST-5-002**	99.4	55/45

[a] All final compounds are >95% pure by HPLC analysis.

CI 3a. HPLC analysis report of **ST-2-001**

<Chromatogram>

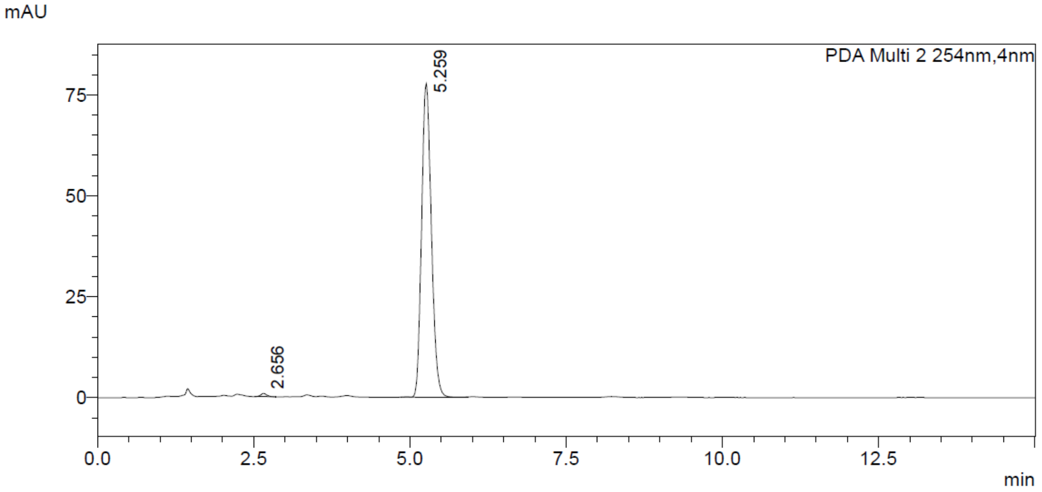

<Peak Table>

PDA Ch2 254nm

Peak#	Ret. Time	Area	Height	Area%	Height%
1	2.656	4777	745	0.562	0.950
2	5.259	845535	77691	99.438	99.050
Total		850312	78437	100.000	100.000

CI 3b. HPLC analysis report of **ST-3-001**

<Chromatogram>

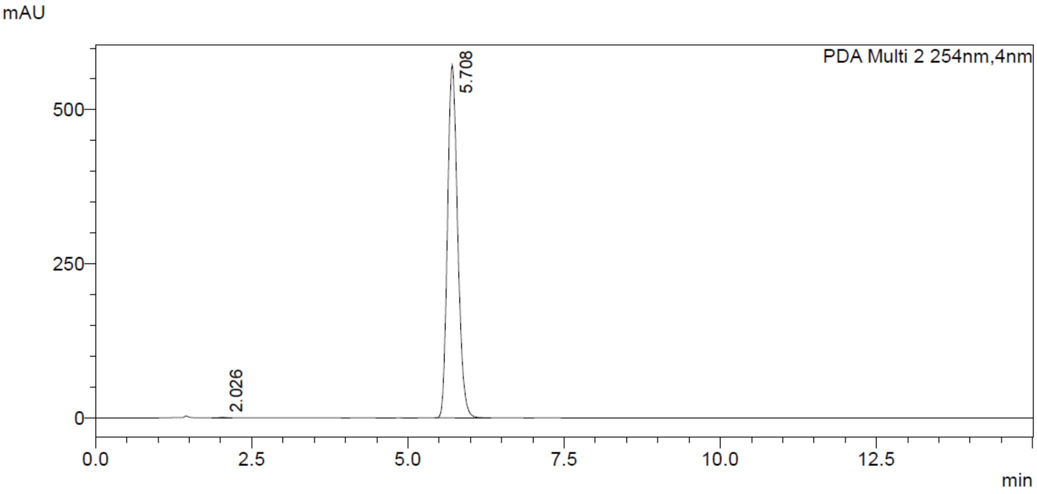

<Peak Table>

PDA Ch2 254nm

Peak#	Ret. Time	Area	Height	Area%	Height%
1	2.026	4293	794	0.066	0.138
2	5.708	6499310	572767	99.934	99.862
Total		6503602	573561	100.000	100.000

CI 3c. HPLC analysis report of **ST-5-001**

<Chromatogram>

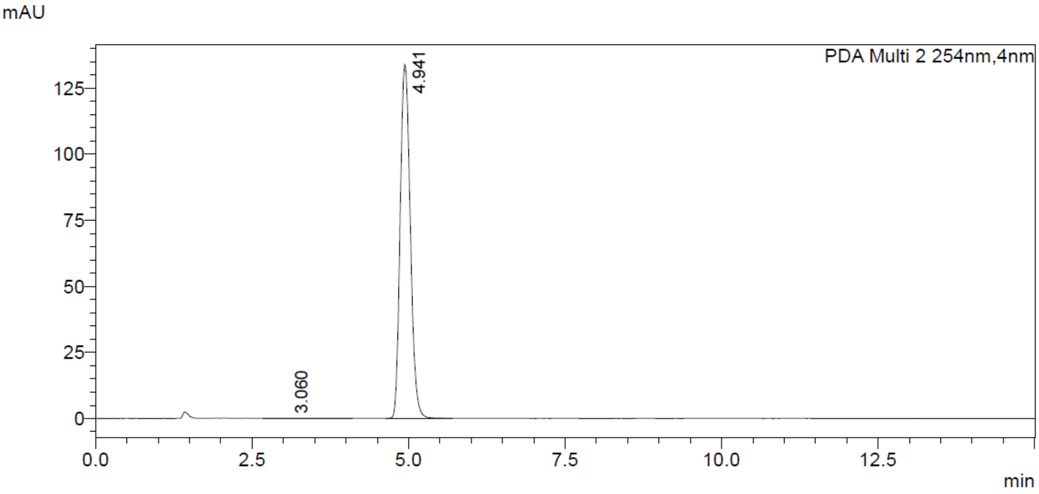

<Peak Table>

PDA Ch2 254nm

Peak#	Ret. Time	Area	Height	Area%	Height%
1	3.060	2761	65	0.183	0.049
2	4.941	1509130	133914	99.817	99.951
Total		1511891	133980	100.000	100.000

CI 3d. HPLC analysis report of **ST-4-005**

<Chromatogram>

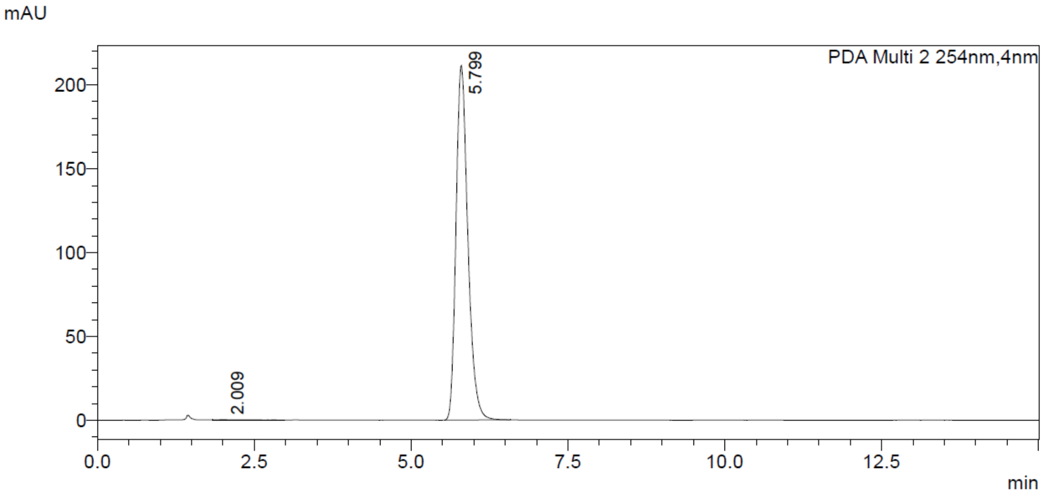

<Peak Table>

PDA Ch2 254nm

Peak#	Ret. Time	Area	Height	Area%	Height%
1	2.009	19551	467	0.699	0.221
2	5.799	2777788	211340	99.301	99.779
Total		2797339	211808	100.000	100.000

CI 3e. HPLC analysis report of **ST-5-002**

<Chromatogram>

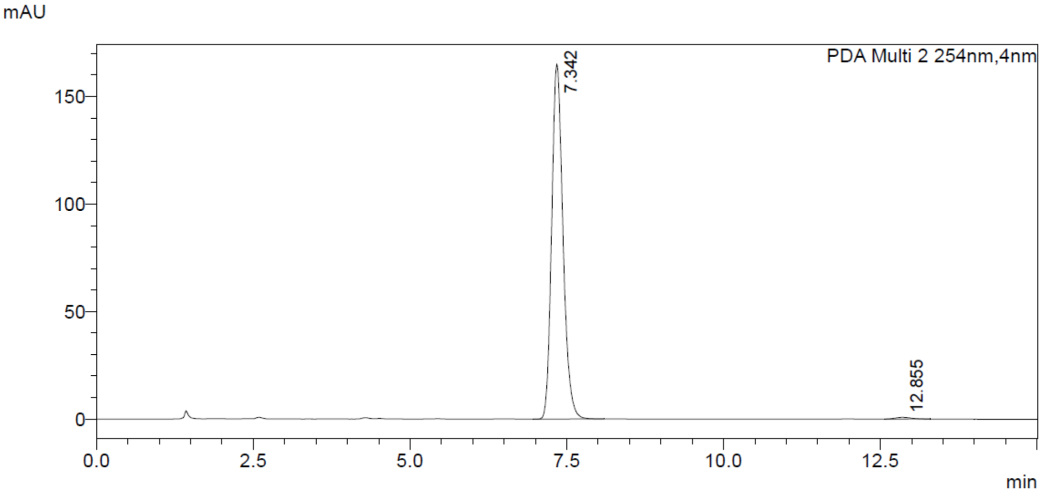

<Peak Table>

PDA Ch2 254nm

Peak#	Ret. Time	Area	Height	Area%	Height%
1	7.342	2077264	164915	99.383	99.550
2	12.855	12905	746	0.617	0.450
Total		2090169	165662	100.000	100.000

V. Preparation and analysis of [^{14}C]-labeled ST-5-002 radio-active compound

[^{14}C]ST-5-002 was synthesized by Curachem, Inc. according to the synthetic procedure provided by Y.-G. Suh research laboratory. (Curachem reference, DOC(NG)-C1000-01P)

*1. Preparation of **[^{14}C]ST-5-002***

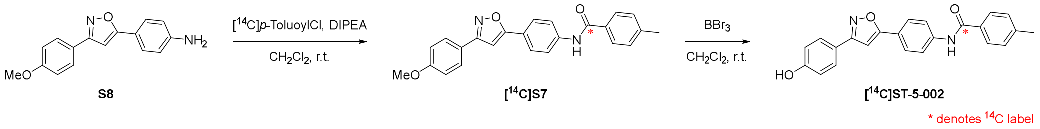

*1.1. N-(4-(3-(4-Methoxyphenyl)isoxazol-5-yl)phenyl)-4-methylbenzamide (**[^{14}C]S7**)*

To a suspension of **S8** (186 mg, 698 μmol) in dichloromethane (7.0 mL) was added a solution of [^{14}C]*p*-toluoyl chloride (1.76 GBq) in dichloromethane (8.0 mL) at ambient temperature over 5 min. To the mixture was added *N*,*N*-diisopropylethylamine (430 μL, 2.53 mmol) at ambient temperature. After stirring for 2 h, the reaction mixture quenched with H_2O, extracted with dichloromethane, and washed with brine. The combined organic layers was concentrated to give crude **[^{14}C]S7**. To the crude **[^{14}C]S7** was added dichloromethane (4.0 mL) and stirred at ambient temperature for 1 h. The mixture was filtered and washed with dichloromethane (1.0 mL). The obtained solid was dried *in vacuo* for 1 h to give **[^{14}C]S7** (250 mg, 650 μmol).

*1.2. N-(4-(3-(4-Hydroxyphenyl)isoxazol-5-yl)phenyl)-4-methylbenzamide (**[^{14}C]ST-5-002**)*

To a suspension of **[^{14}C]S7** (250 mg, 650 μmol) in dichloromethane (4.5 mL) was added a solution of boron tribromide (1.0 M in dichloromethane) (2.00 mL, 2.00 mmol) at ambient temperature over 5 min. After stirring for 2 h, the reaction mixture was quenched with H_2O, and extracted with dichloromethane and saturated NH_4Cl solution. The combined organic layer was concentrated and the residue was purified by column chromatography on silica-gel (CH_2Cl_2/MeOH = 9:1). The desired fractions were combined and concentrated to give a crude **[^{14}C]ST-5-002** (873 MBq). The crude **[^{14}C]ST-5-002** (873 MBq) was purified by column chromatography on octadecyl silica gel (MeOH/H_2O = 2:1 to 10:1) to afford crude **[^{14}C]ST-5-002** (122 mg). To the crude **[^{14}C]ST-5-002** (122 mg) was added acetone (2.0 mL). After stirring for 1 h, the mixture was filtered and washed with acetone (1.0 mL). The obtained solid was dried *in vacuo* to give **[^{14}C]ST-5-002** (601 MBq, 114 mg, 308 μmol, Batch No. K1362): ^{1}H NMR (400 MHz, DMSO-d_6) δ 10.42 (s, 1H), 9.94 (s, 1H), 7.99 (d, J = 8.4 Hz, 2H), 7.90 (m, 4H), 7.74 (d, J = 8.0 Hz, 2H), 7.55 (m, 3H), 6.91 (d, J = 8.4 Hz, 2H), 2.40 (s, 3H); LR-MS (APIC+) *m/z* 373.13 (M+H$^+$).

*2. Analysis of **[^{14}C]ST-5-002***

2.1. Specific radioactivity

The specific radioactivity was 5.28 MBq/mg (1.97 GBq/mmol) as determined by gravimetric assay.

2.2. Chemical and radio-chemical purity

The chemical purity was 99.2% as determined by HPLC analysis with Waters Alliance e2695. HPLC chromatograms were obtained under the following conditions; Column, SunFire® C18, 4.6 x 150 mm, 5 µm; detection, UV 254 nm; flow rate 1.0 mL/min; Mobile phase, CH_3CN(%)/H_2O(%) = 55/45, Isocratic.

CI 4a. HPLC analysis report of **ST-5-002**

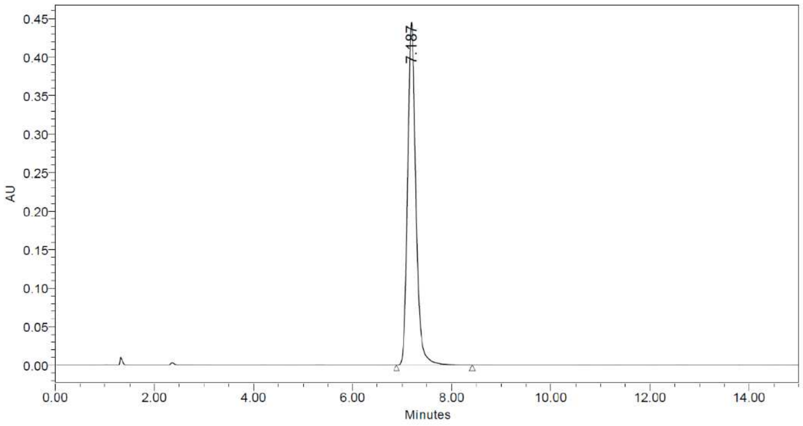

	Name	RT	Height	Area	% Area
1		7.187	445086	5161659	100.00

CI 4b. HPLC analysis report of **[^{14}C]ST-5-002**

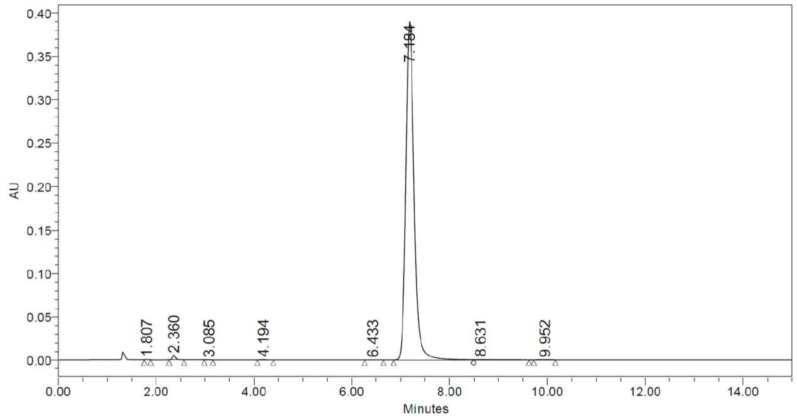

	Name	RT	Height	Area	% Area
1		1.807	574	1803	0.04
2		2.360	5452	26354	0.57
3		3.085	52	285	0.01
4		4.194	165	1201	0.03
5		6.433	57	642	0.01
6		7.184	389786	4552975	99.18
7		8.631	215	6370	0.14
8		9.952	93	1199	0.03

The radio-chemical purity was 99.7% as determined by radioisotope detection HPLC (radio detector) under the following conditions.

Table S2. Radio detector information and conditions

**Radio detector**	Berthold FlowStar^2 LB514
**Integrator**	RadioStar
**Measuring cell volume**	500 μL
**Static mixer volume**	150 μL
**Liquid scintillator pump flow**	3.0 mL/min
**Radio channel**	^{14}C
**Dwell time**	6 Sec
**Cocktail**	FLO-SCINT^{TM} II, PerkinElmer

CI 4c. HPLC radio analysis report of **[^{14}C]ST-5-002**

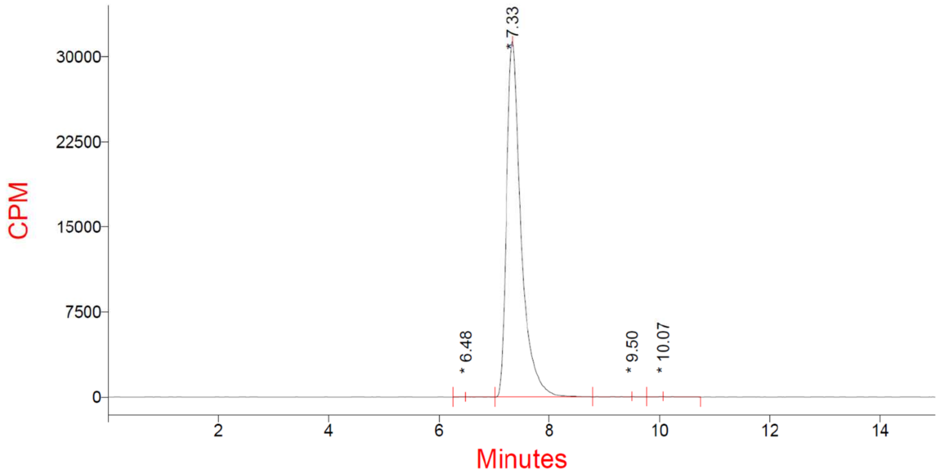

#	Peak name	Peak start	Rt.	Peak end	Net CPM	% ROI
1		6.25	6.48	7.02	209.87	0.04
2		7.02	7.33	8.78	569249.87	99.67
3		8.78	9.50	9.77	1008.27	0.18
4		9.77	10.07	10.75	692.00	0.12
**SUM**					**571160.00**	**100.00**

2.3. ^{1}H NMR spectra

[^{14}C]ST-5-002 was confirmed by comparing its ^{1}H NMR spectra with that of the unlabeled compound **ST-5-002**. ^{1}H NMR was recorded by VNMRS-400 (400 MHz, Varian, Rancho Cordova, CA, USA).

CI 4d. ^{1}H NMR spectra of standard **ST-5-002**

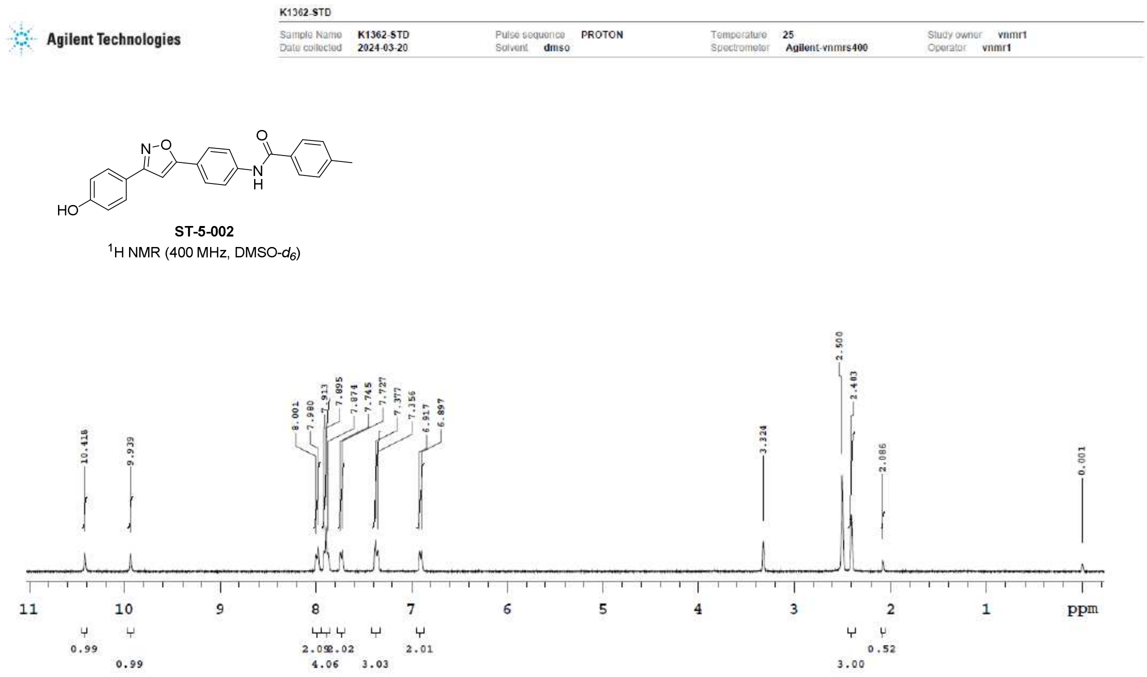

CI 4e. ^{1}H NMR spectra of **[^{14}C]ST-5-002**

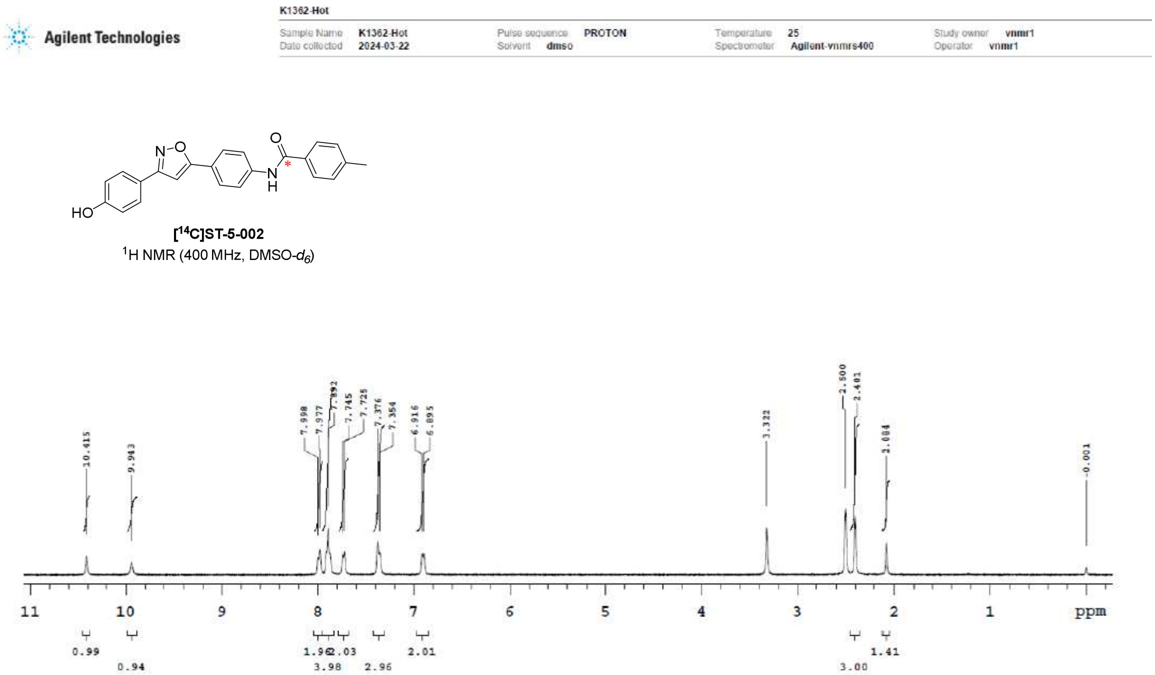

2.4. Mass analysis

[^{14}C]ST-5-002 was confirmed by comparing its mass spectrum with that of the unlabeled **ST-5-002**. Mass spectra was analyzed by ion-trap mass spectrometer under the following conditions.

Table S3. Mass spectrometer information and conditions

Thermo LCQ-Fleet			
**Ionization source**	APCI	**Sheath gas flow rate**	50 psi
**Mass range (m/z)**	170 to 570	**Ion spray voltage**	5.0 kW
**Polarity**	Positive	**Capillary temp.**	275 ºC
**Vaporizer temp.**	450 ºC	**Capillary voltage**	10 V
**Injection volume**	50 μL	**Tube lens offset**	70 V

CI 4f. Mass spectra of standard **ST-5-002**

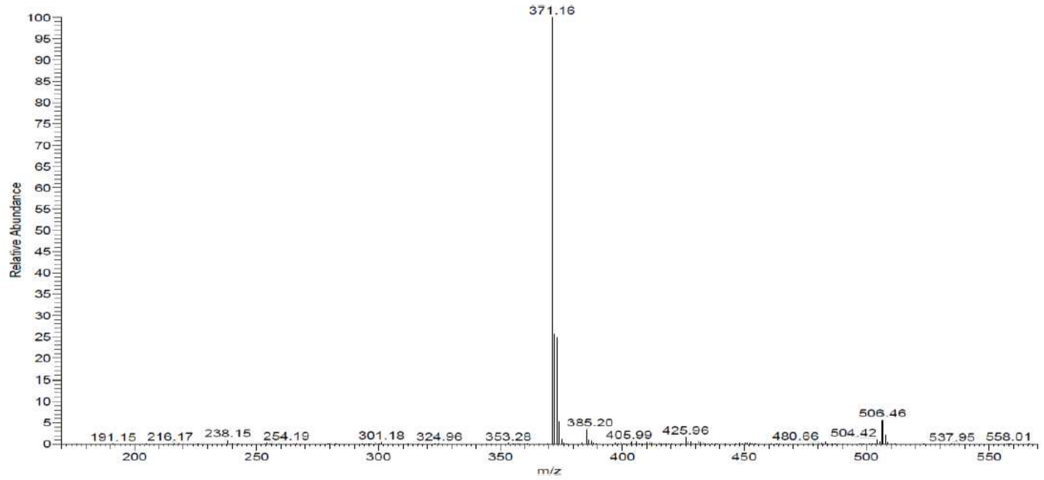

CI 4g. Mass spectra of **[^{14}C]ST-5-002**

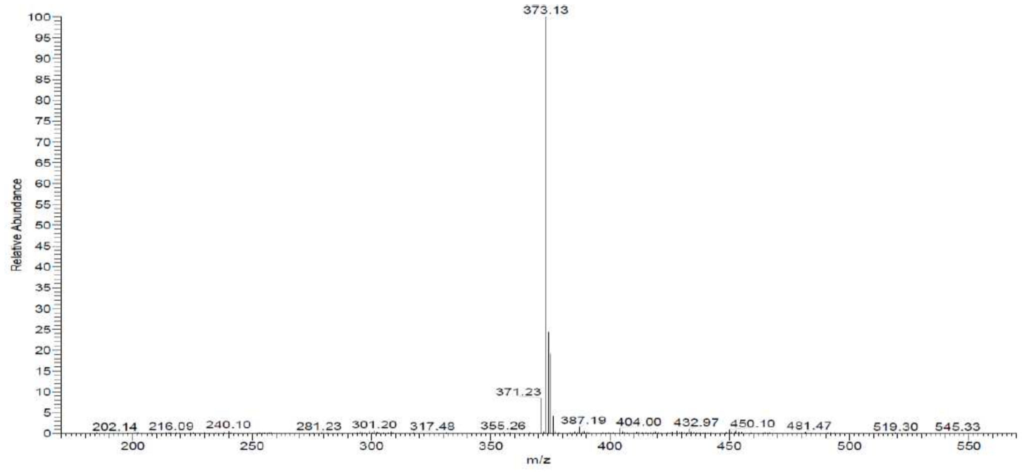

Supplementary Gating Strategy (GS)

List of Contents

GS1. Gating strategies for mouse intrahepatic or splenic NK cell population …………………………p41

GS2. Gating strategies for mouse liver or spleen surface staining (for intrahepatic or splenic Slamf7+ NK cell population)…………………………………… …………………p41

GS3. Gating Strategies for mouse liver or spleen intracellular staining (for intrahepatic Gzmb+ or splenic Ifn-γ+ NK cells)…...……………………………………………………p42

GS4. Gating Strategies for mouse liver or spleen intracellular staining (for intrahepatic or splenic Prf+ NK cells)……………………………………………………………………p42

GS1

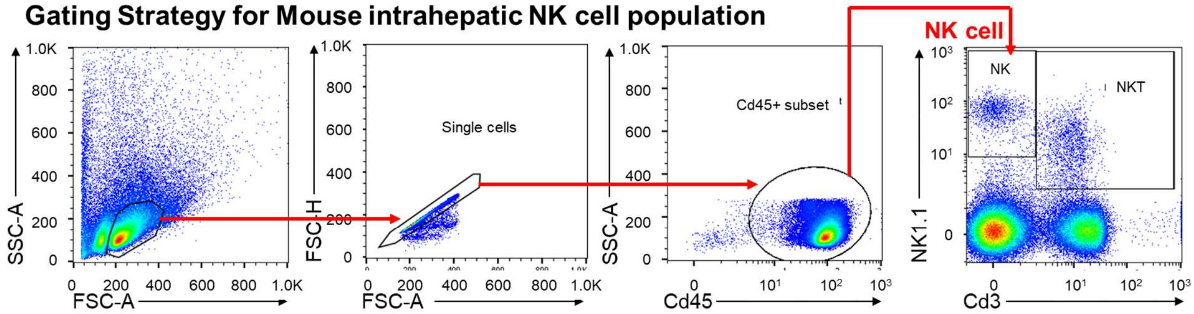
Gating Strategy for Mouse intrahepatic NK cell population
NK cell
Single cells
Cd45+ subset
NK
NKT
SSC-A
FSC-A
FSC-H
Cd45
NK1.1
Cd3

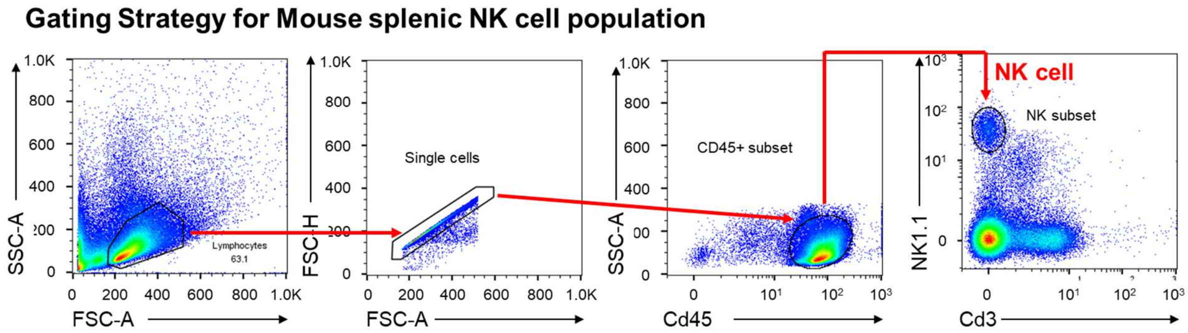
Gating Strategy for Mouse splenic NK cell population
NK cell
Lymphocytes
63.1
Single cells
CD45+ subset
NK subset
SSC-A
FSC-A
FSC-H
Cd45
NK1.1
Cd3

GS2

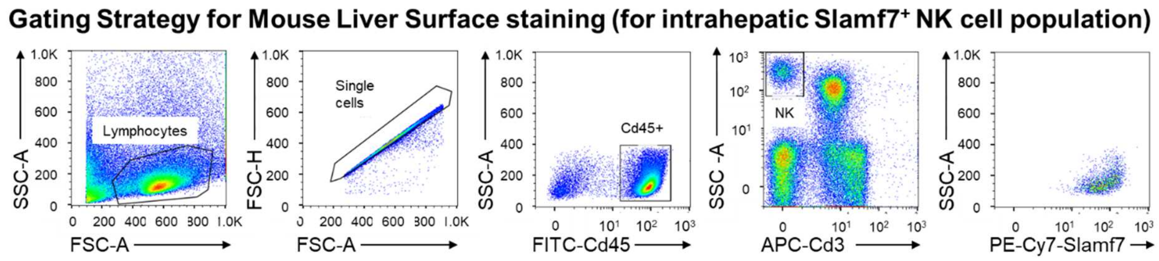
Gating Strategy for Mouse Liver Surface staining (for intrahepatic Slamf7+ NK cell population)
Lymphocytes
Single cells
Cd45+
NK
SSC-A
FSC-A
FSC-H
FITC-Cd45
SSC -A
APC-Cd3
PE-Cy7-Slamf7

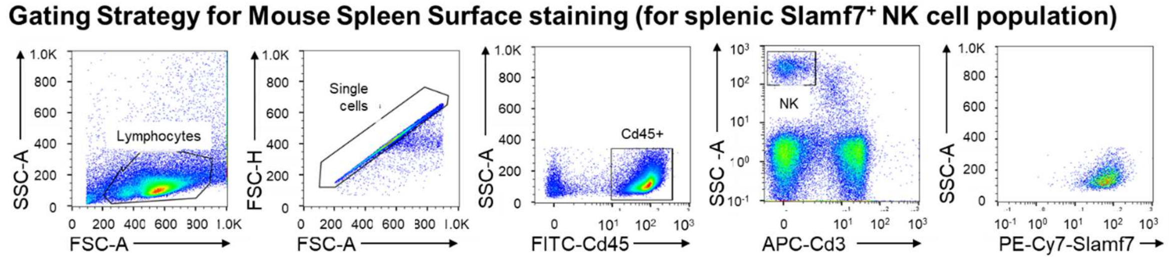
Gating Strategy for Mouse Spleen Surface staining (for splenic Slamf7+ NK cell population)
Lymphocytes
Single cells
Cd45+
NK
SSC-A
FSC-A
FSC-H
FITC-Cd45
SSC -A
APC-Cd3
PE-Cy7-Slamf7

GS3

Gating Strategy for Mouse Liver Intracellular staining (for intrahepatic Gzmb+ NK cells)

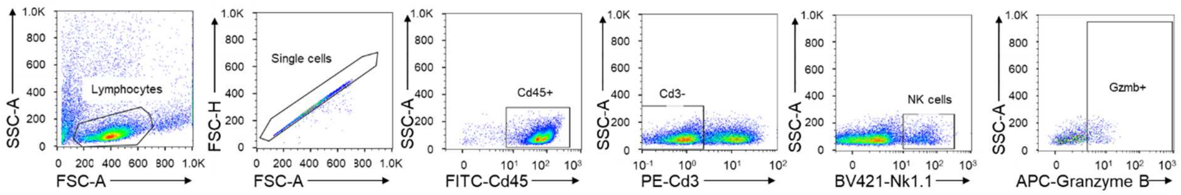

Gating Strategy for Mouse Spleen Intracellular staining (for splenic Ifn-γ⁺ NK cells)

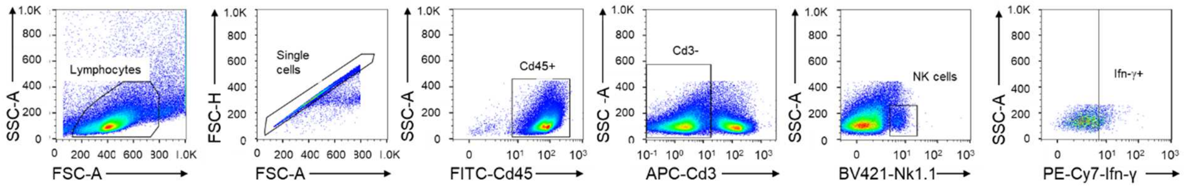

GS4

Gating Strategy for Mouse Liver Intracellular staining (for intrahepatic Prf⁺ NK cells)

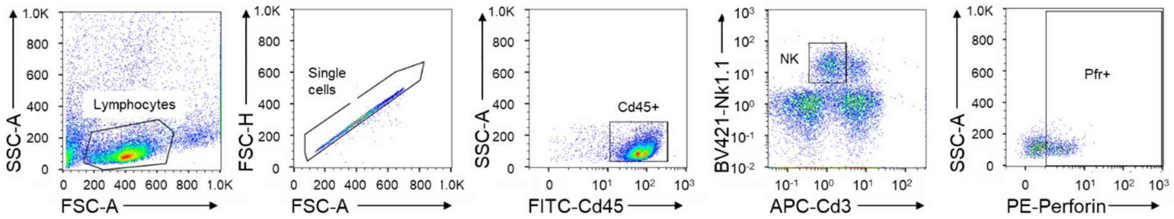

Gating Strategy for Mouse Spleen Intracellular staining (for splenic Prf+ NK cells)

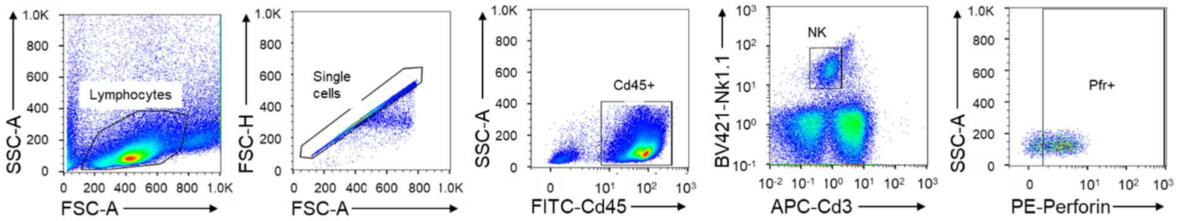

Supplement: Supplementary file 1 — Supplementary Materials [file 41392_2024_2106_MOESM1_ESM.pdf]
