# Supplementary material for: Isoxazole-based molecules restore NK cell immune surveillance in hepatocarcinogenesis by targeting TM4SF5 and SLAMF7 linkage
Source: Signal Transduct Target Ther. 2025 Jan 20;10:15. doi: 10.1038/s41392-024-02106-6 (PMC11743776; doi:10.1038/s41392-024-02106-6)

Figure 1a

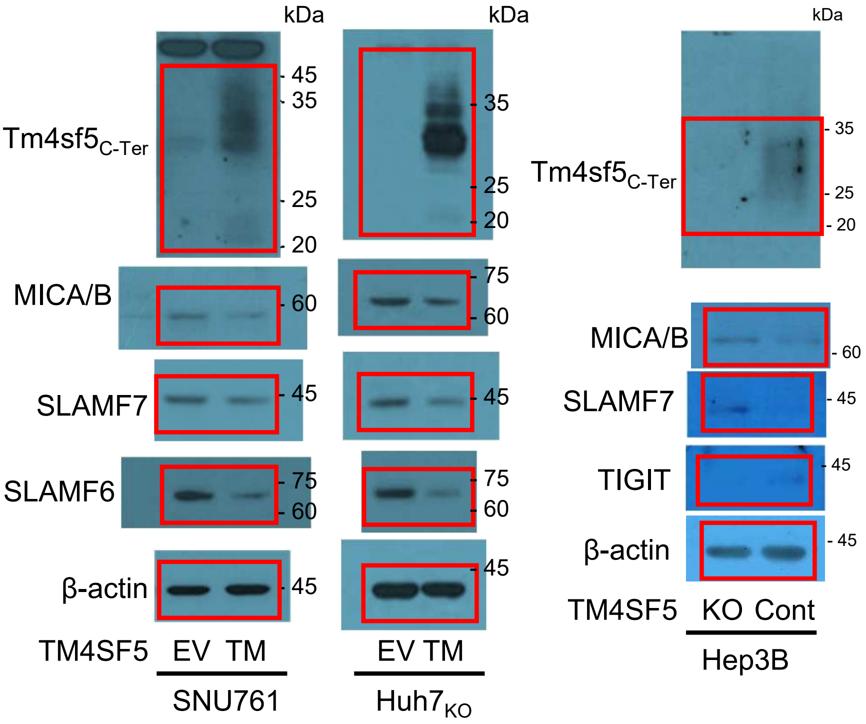

kDa
Tm4sf5C-Ter
45
35
25
20
MICA/B
60
75
SLAMF7
SLAMF6
β-actin
TM4SF5
EV TM
SNU761
Huh7KO
TIGIT
KO Cont
Hep3B

Figure 2d

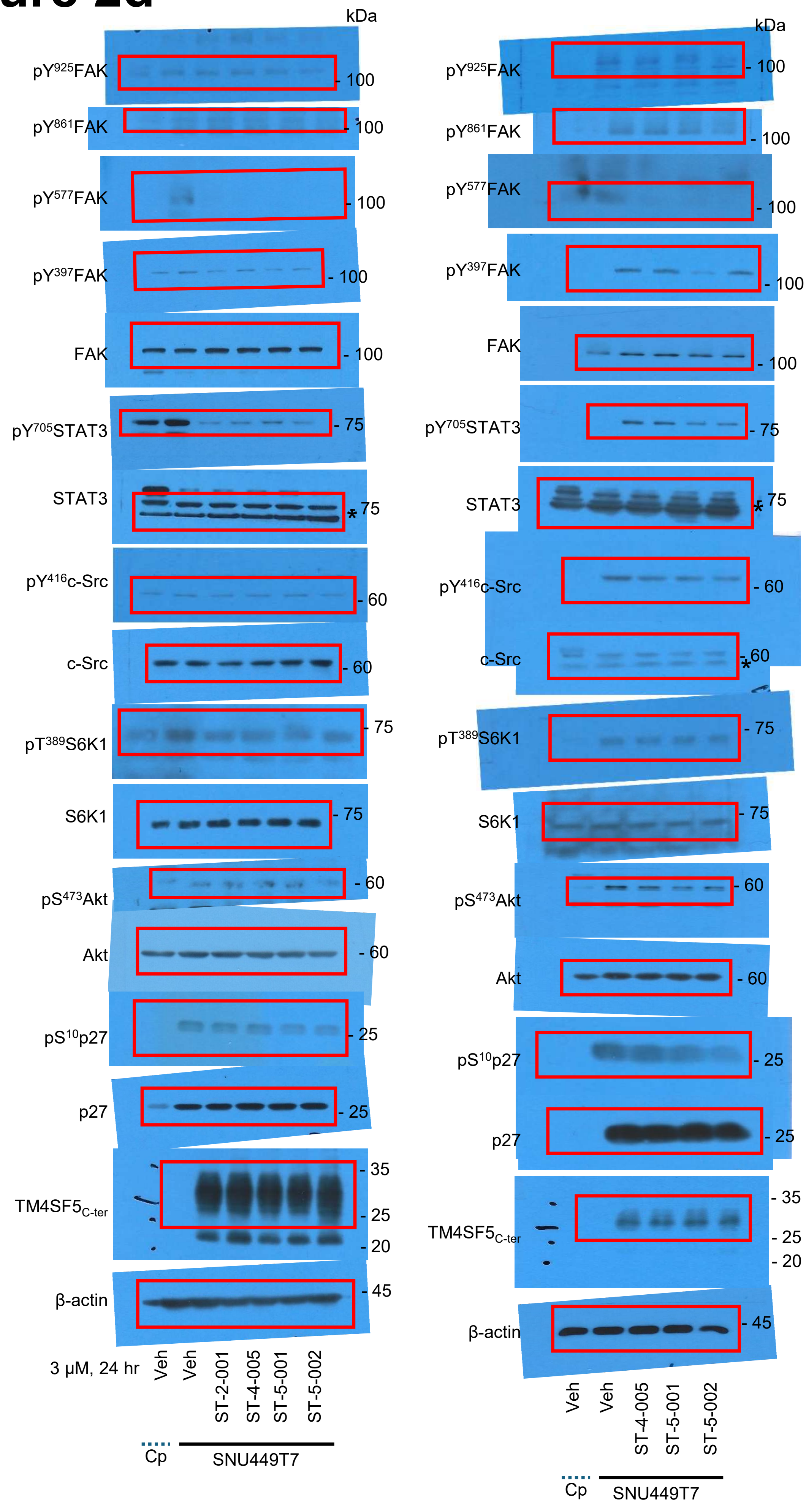
kDa
pY925FAK
pY861FAK
pY577FAK
pY397FAK
FAK
pY705STAT3
STAT3
pY416c-Src
c-Src
pT389S6K1
S6K1
pS473Akt
Akt
pS10p27
p27
TM4SF5C-ter
β-actin
100
75
60
45
35
25
20
*
3 μM, 24 hr
Veh
Veh
ST-2-001
ST-4-005
ST-5-001
ST-5-002
Cp
SNU449T7

Figure 3e

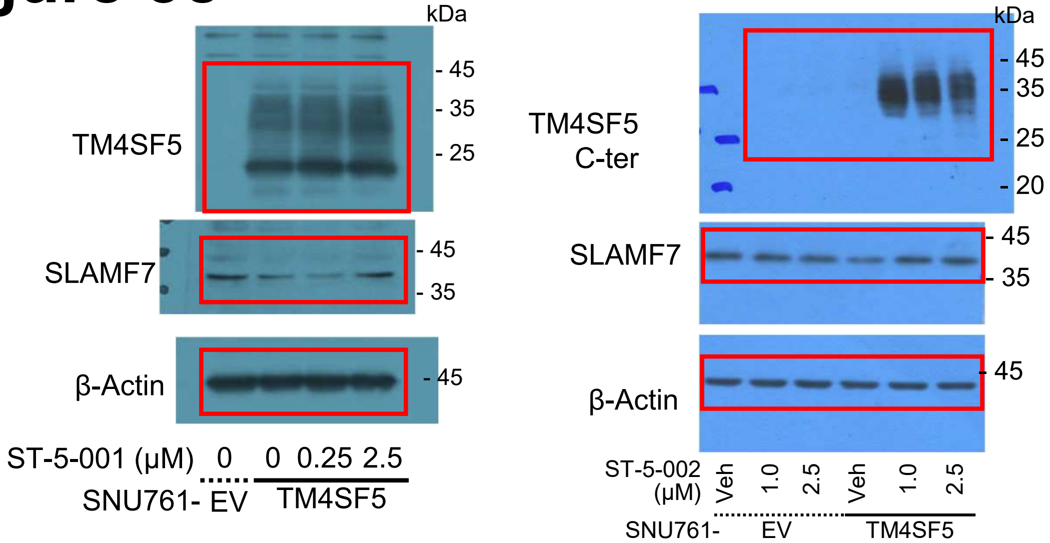

Figure 4a

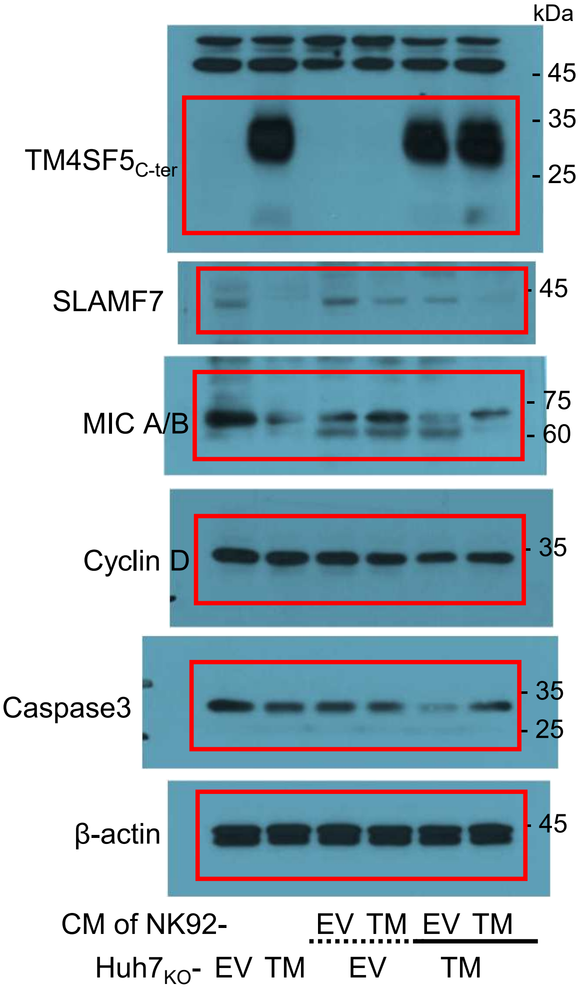

Figure 4b

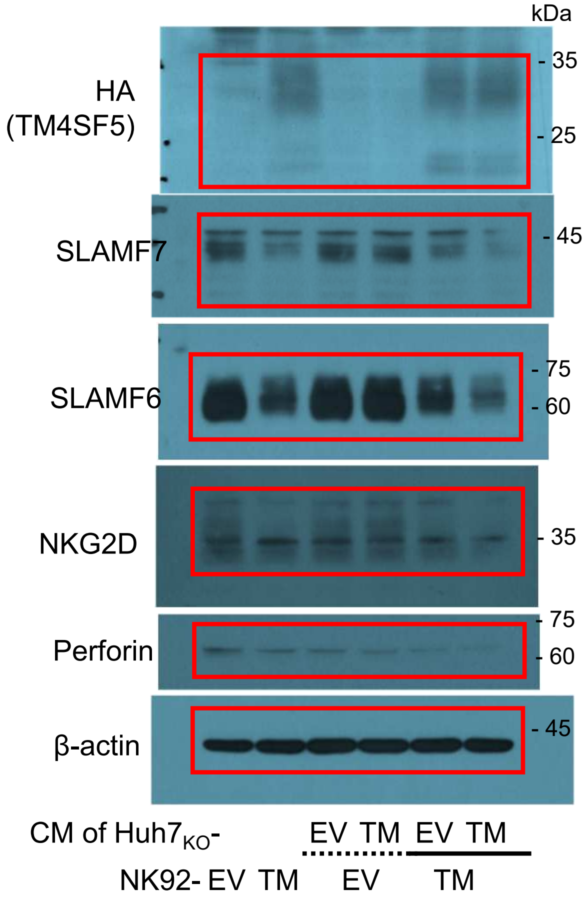

Figure 4c

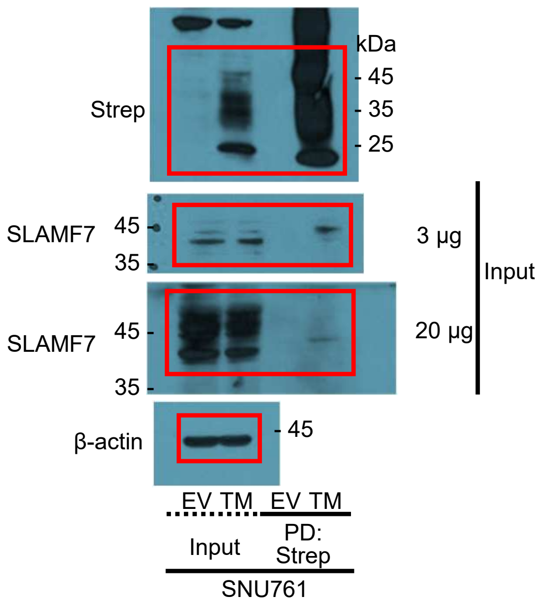

Figure 4d

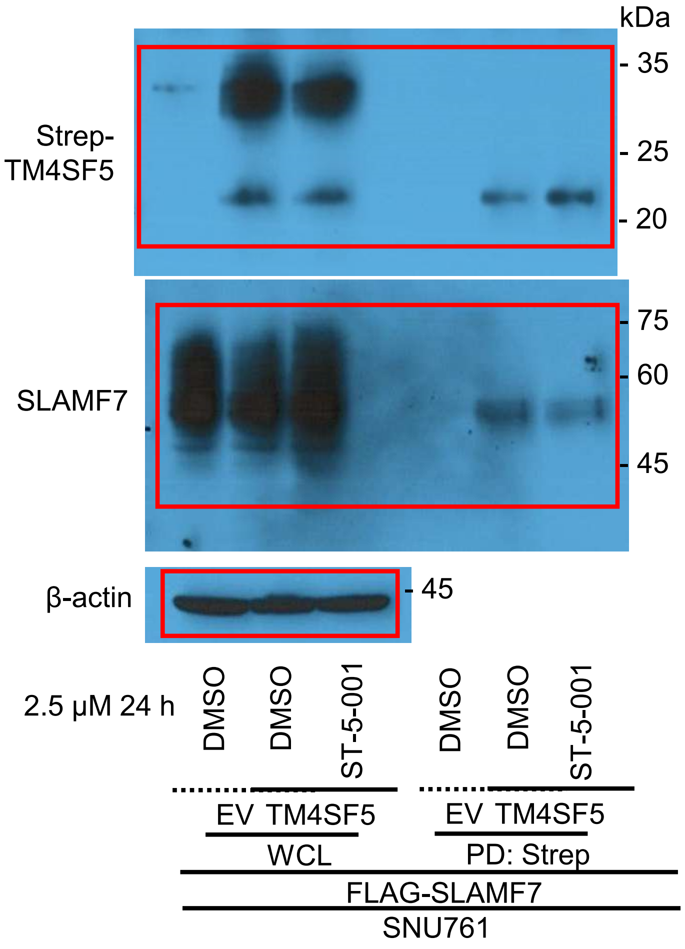

Figure 4e

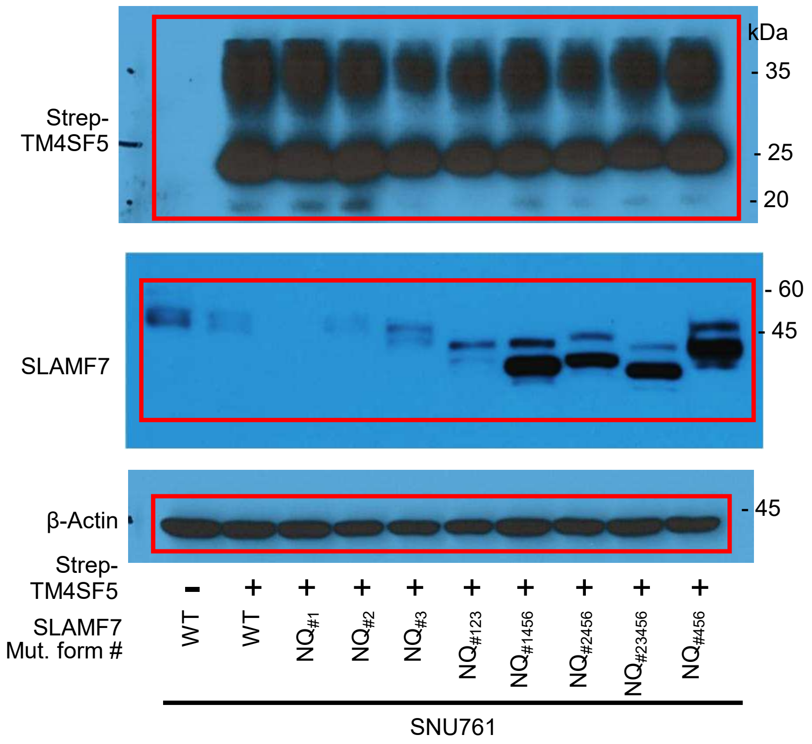

Figure 4f

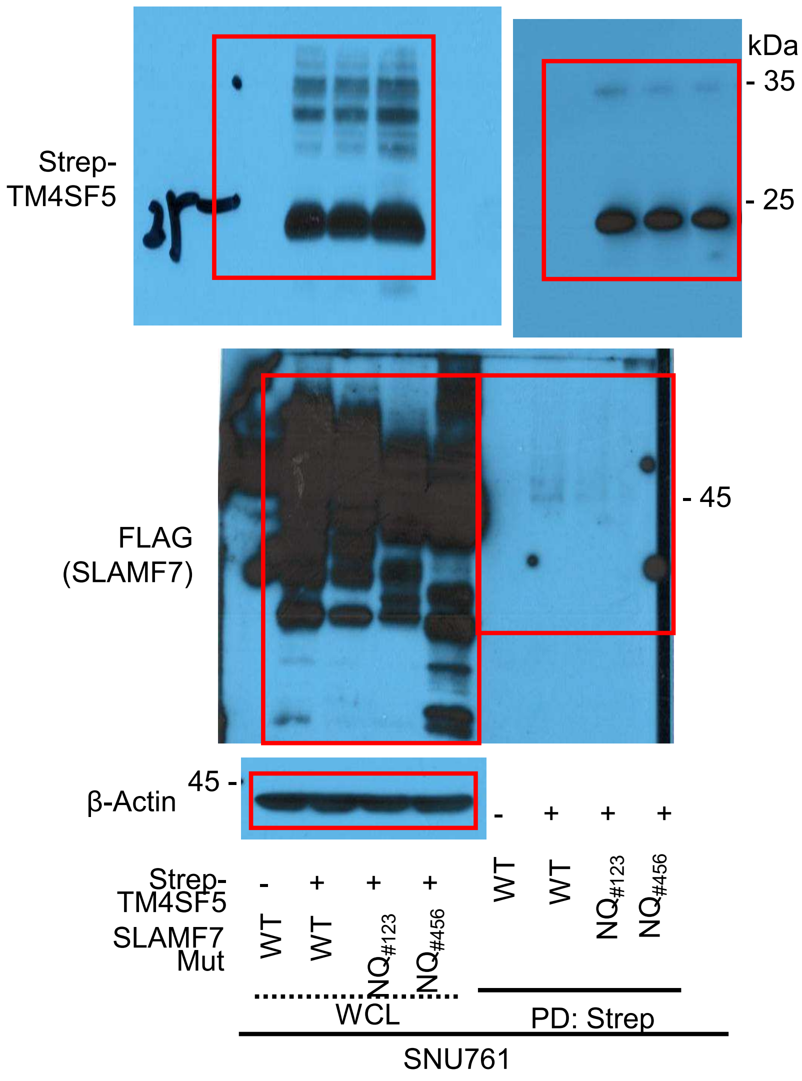

Figure 4g

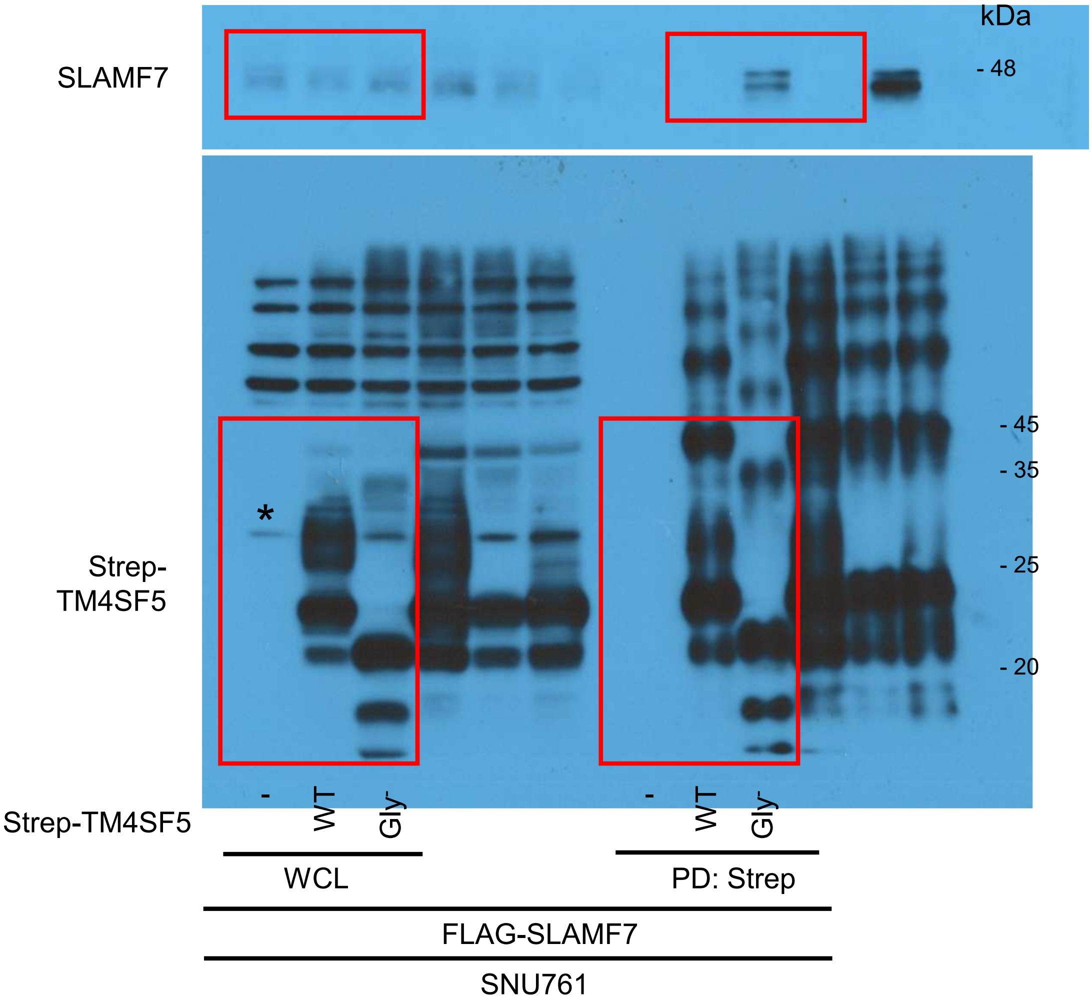

kDa
SLAMF7
- 48
Strep-
TM4SF5
- 45
- 35
*
- 25
- 20
Strep-TM4SF5
-
WT
Gly
-
WT
Gly
WCL
PD: Strep
FLAG-SLAMF7
SNU761

Figure 4h

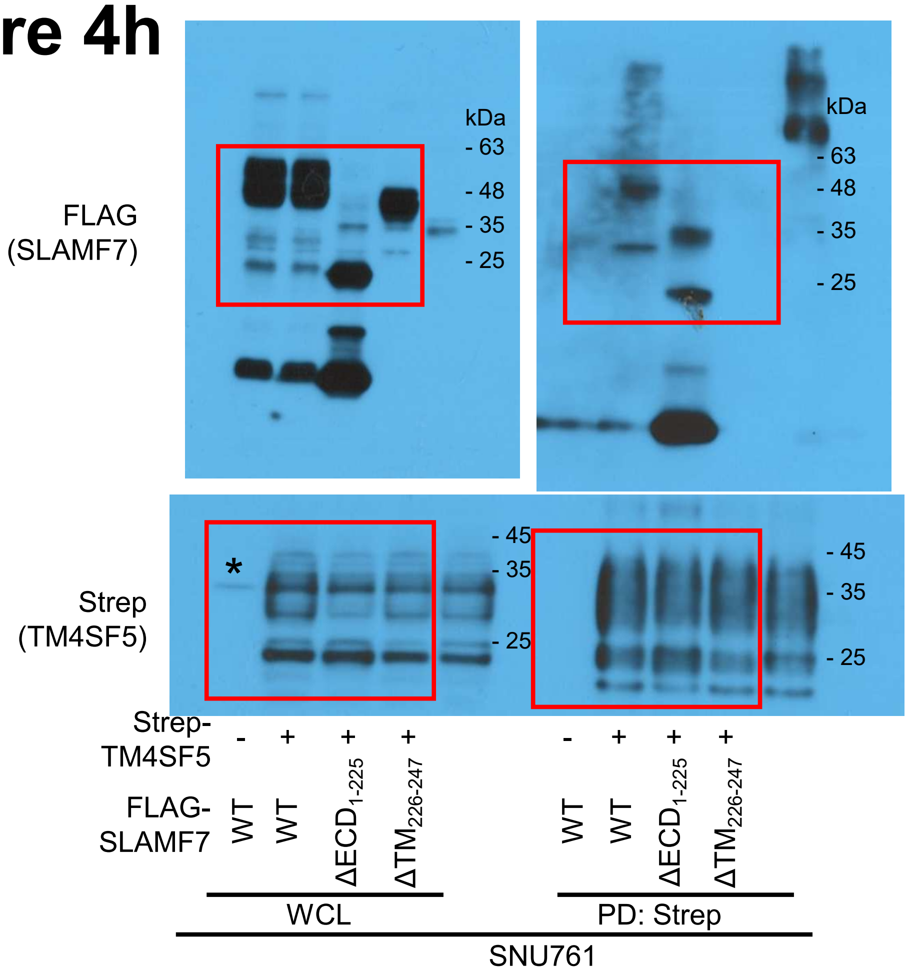

Figure 4i

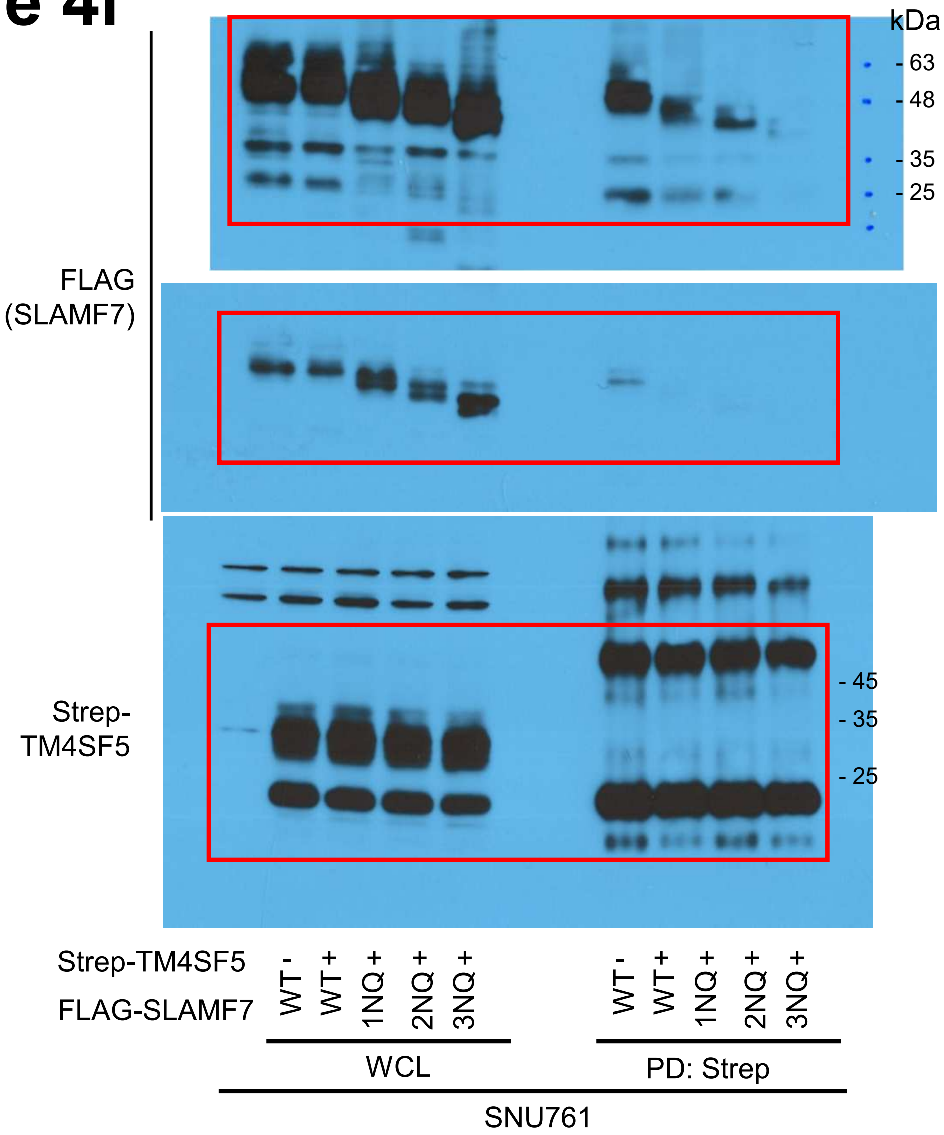

Figure 5a

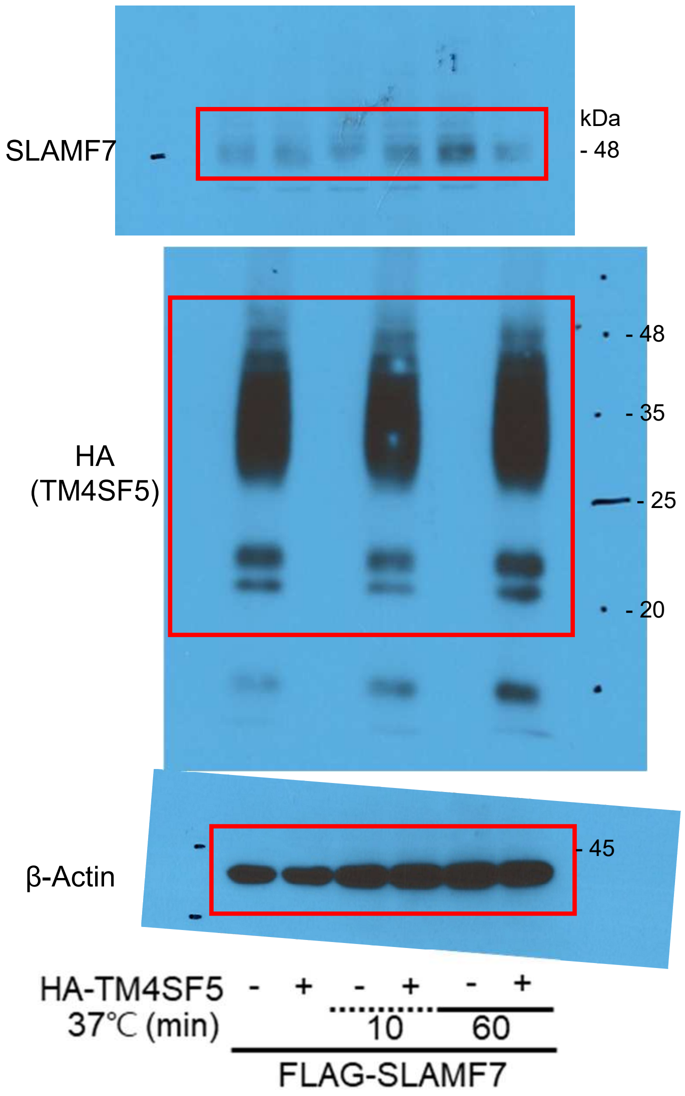

SLAMF7
kDa
- 48
HA
(TM4SF5)
- 48
- 35
- 25
- 20
β-Actin
- 45
HA-TM4SF5 - + - + - +
37℃ (min) 10 60
FLAG-SLAMF7

Figure 5d

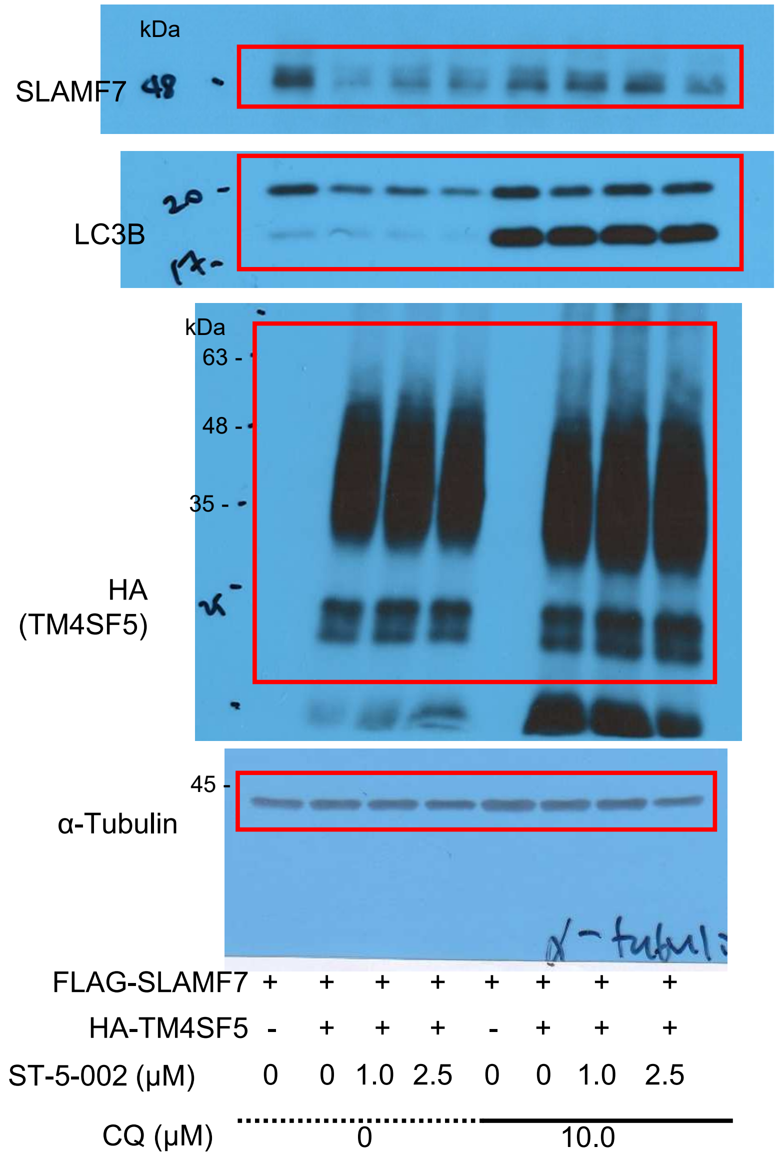

Figure 5e

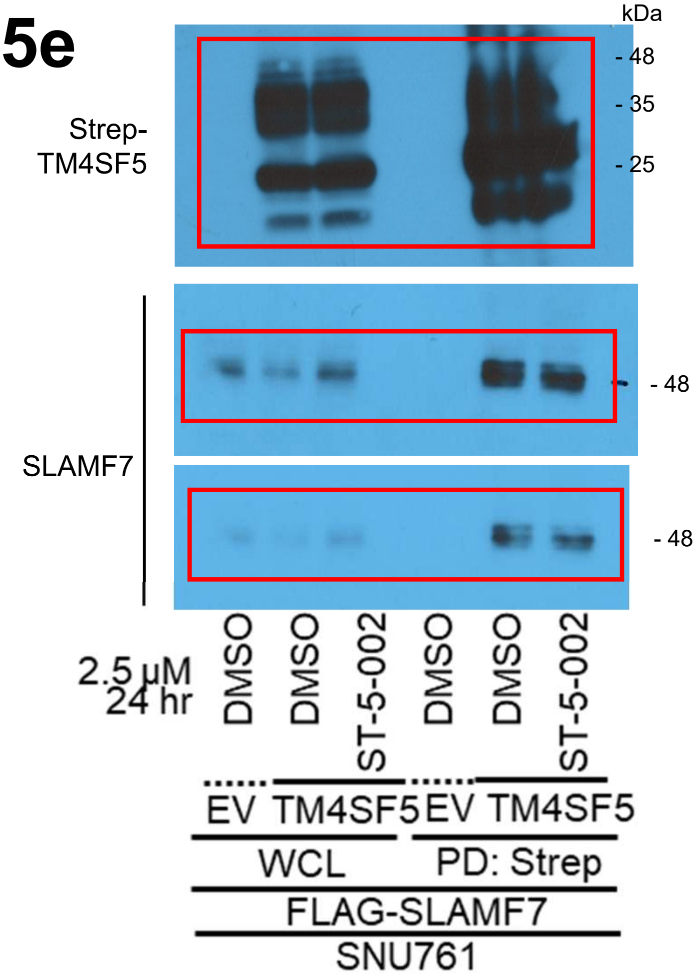

Figure 5f

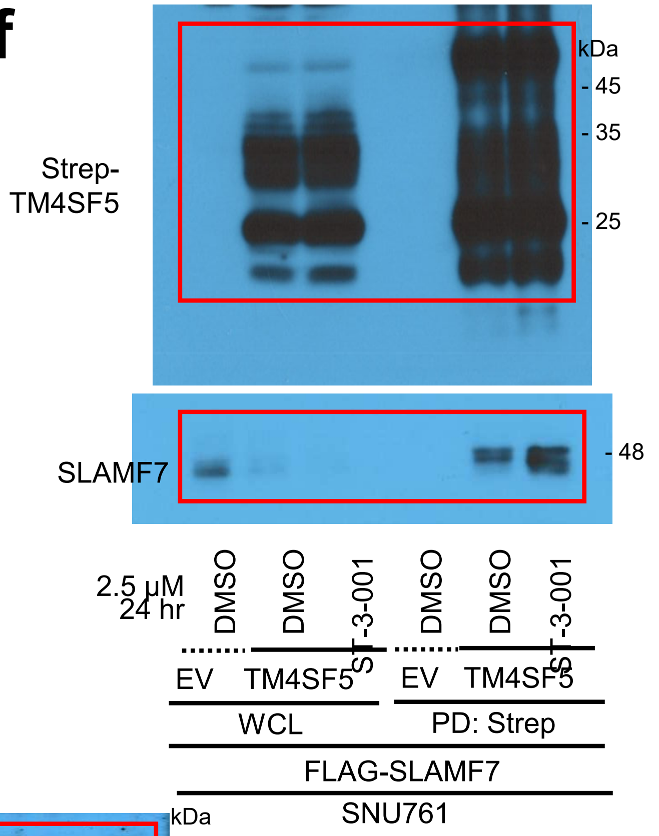

Figure 6e

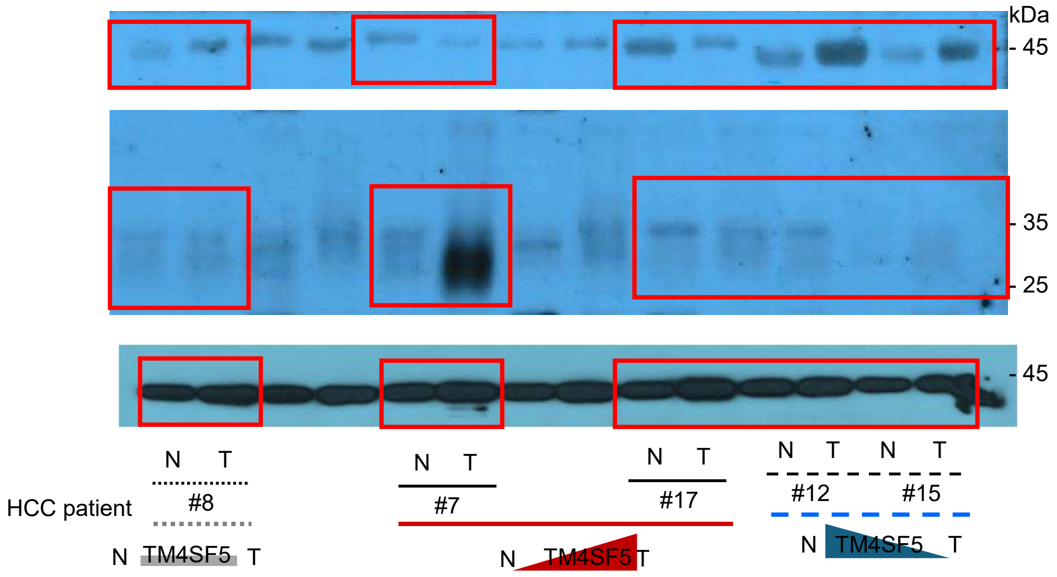

Figure 6f

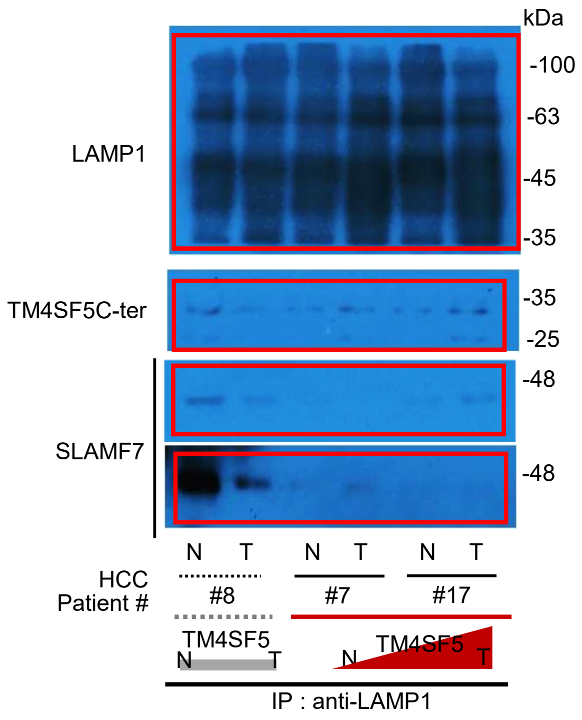

Figure S1a

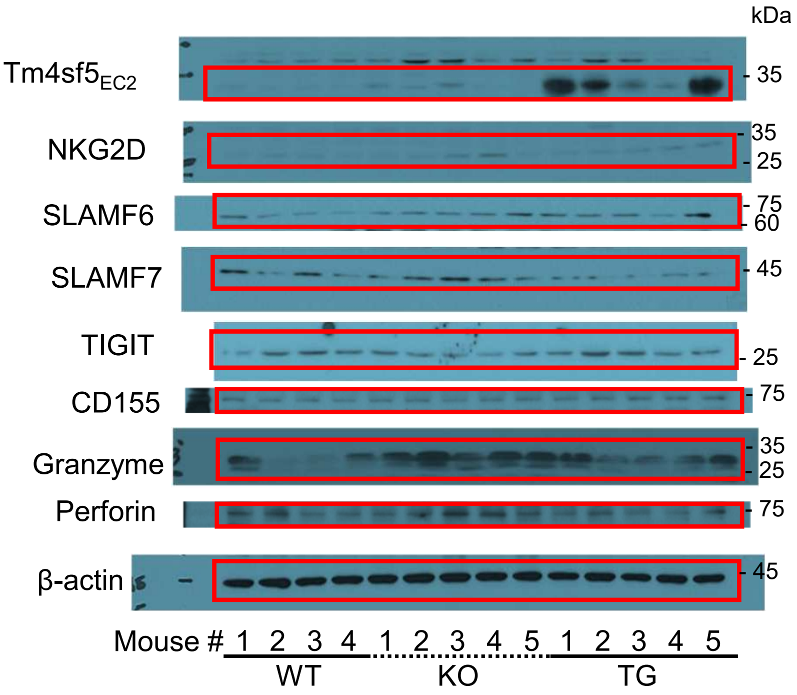
kDa
Tm4sf5EC2
35
NKG2D
35
25
SLAMF6
75
60
SLAMF7
45
TIGIT
25
CD155
75
Granzyme
35
25
Perforin
75
β-actin
45
Mouse # 1 2 3 4 1 2 3 4 5 1 2 3 4 5
WT
KO
TG

Figure S4a

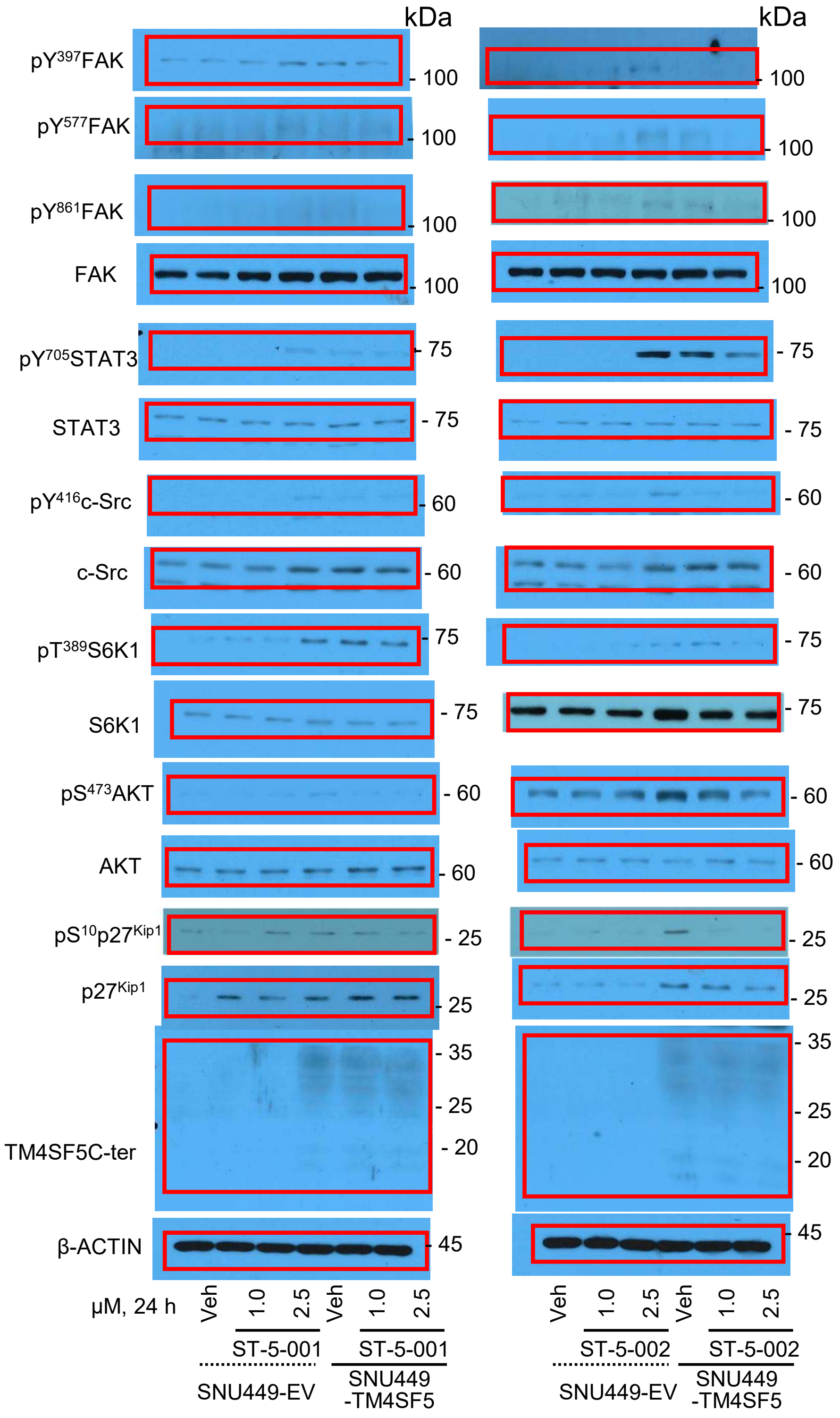

kDa
pY397FAK
pY577FAK
pY861FAK
FAK
pY705STAT3
STAT3
pY416c-Src
c-Src
pT389S6K1
S6K1
pS473AKT
AKT
pS10p27Kip1
p27Kip1
TM4SF5C-ter
β-ACTIN
- 100
- 75
- 60
- 35
- 25
- 20
45
μM, 24 h
Veh
1.0
2.5
ST-5-001
SNU449-EV
SNU449
-TM4SF5
ST-5-002

Figure S4b

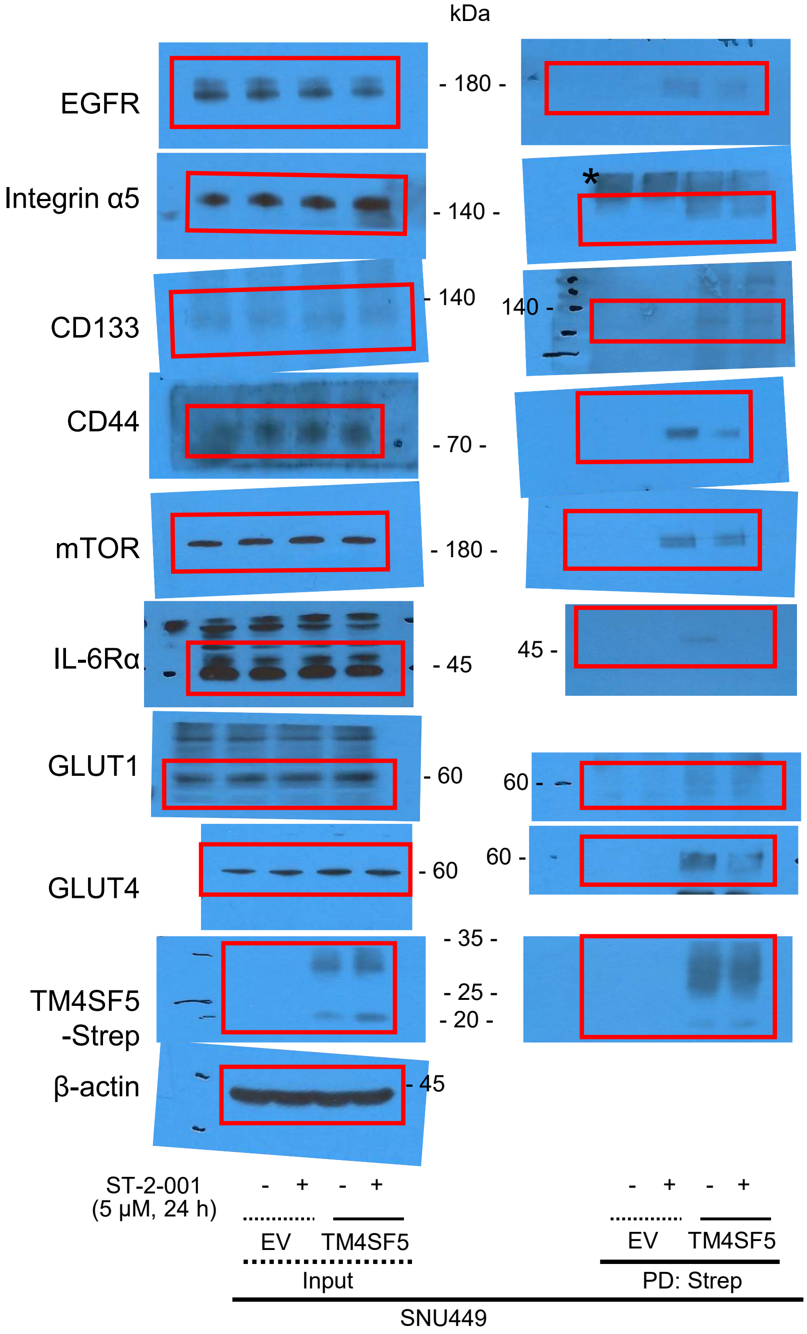

kDa
EGFR
- 180 -
Integrin α5
- 140 -
*
CD133
- 140
140 -
CD44
- 70 -
mTOR
- 180 -
IL-6Rα
- 45
45 -
GLUT1
- 60
60 -
GLUT4
- 60
60 -
TM4SF5
-Strep
- 35 -
- 25 -
- 20 -
β-actin
- 45
ST-2-001
(5 μM, 24 h)
- + - +
- + - +
EV
TM4SF5
EV
TM4SF5
Input
PD: Strep
SNU449

Figure S5

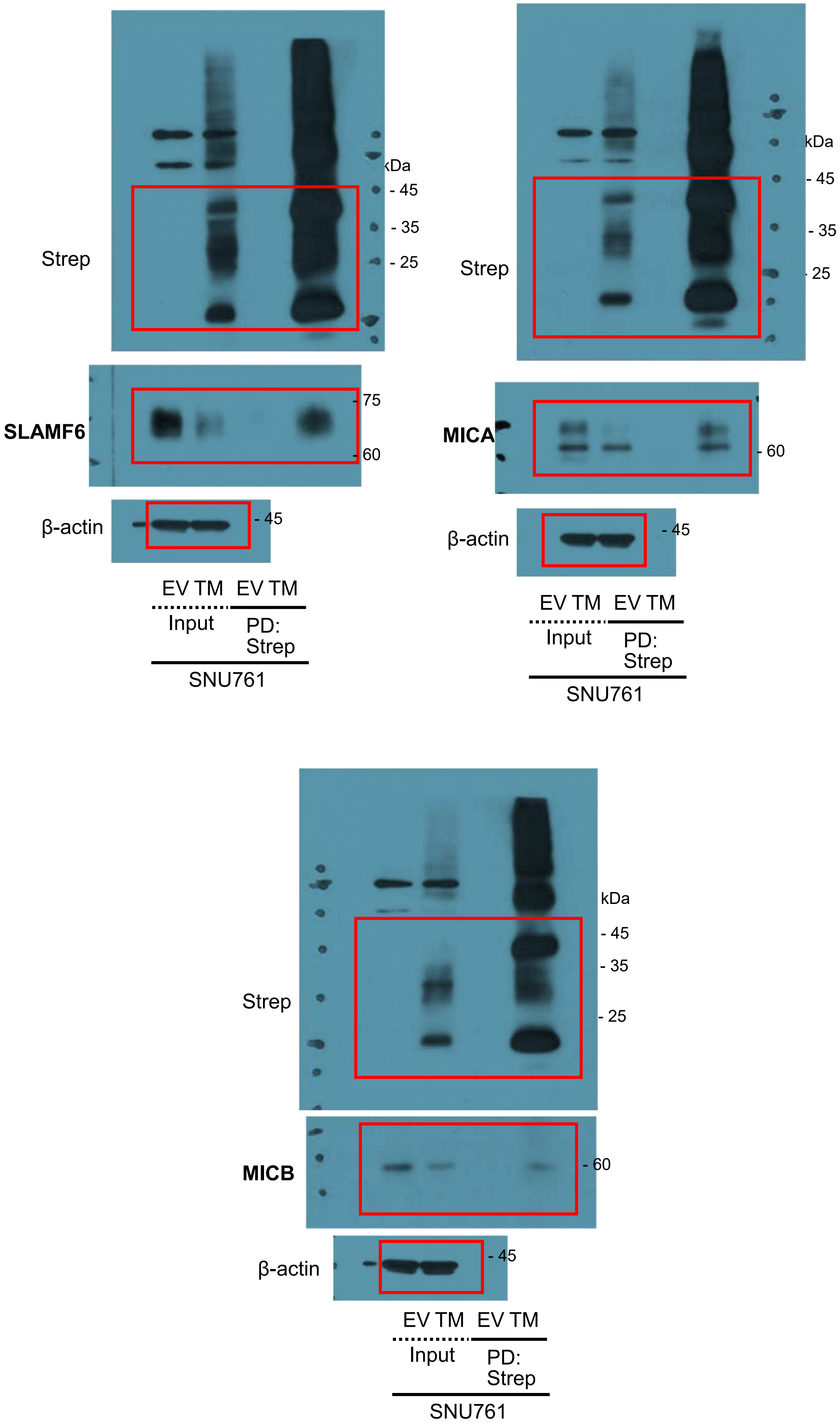
Strep
kDa
- 45
- 35
- 25
SLAMF6
- 75
- 60
β-actin
- 45
EV TM EV TM
Input
PD: Strep
SNU761
Strep
kDa
- 45
- 35
25
MICA
- 60
β-actin
- 45
EV TM EV TM
Input
PD: Strep
SNU761
Strep
kDa
- 45
- 35
- 25
MICB
- 60
β-actin
- 45
EV TM EV TM
Input
PD: Strep
SNU761

Figure S6

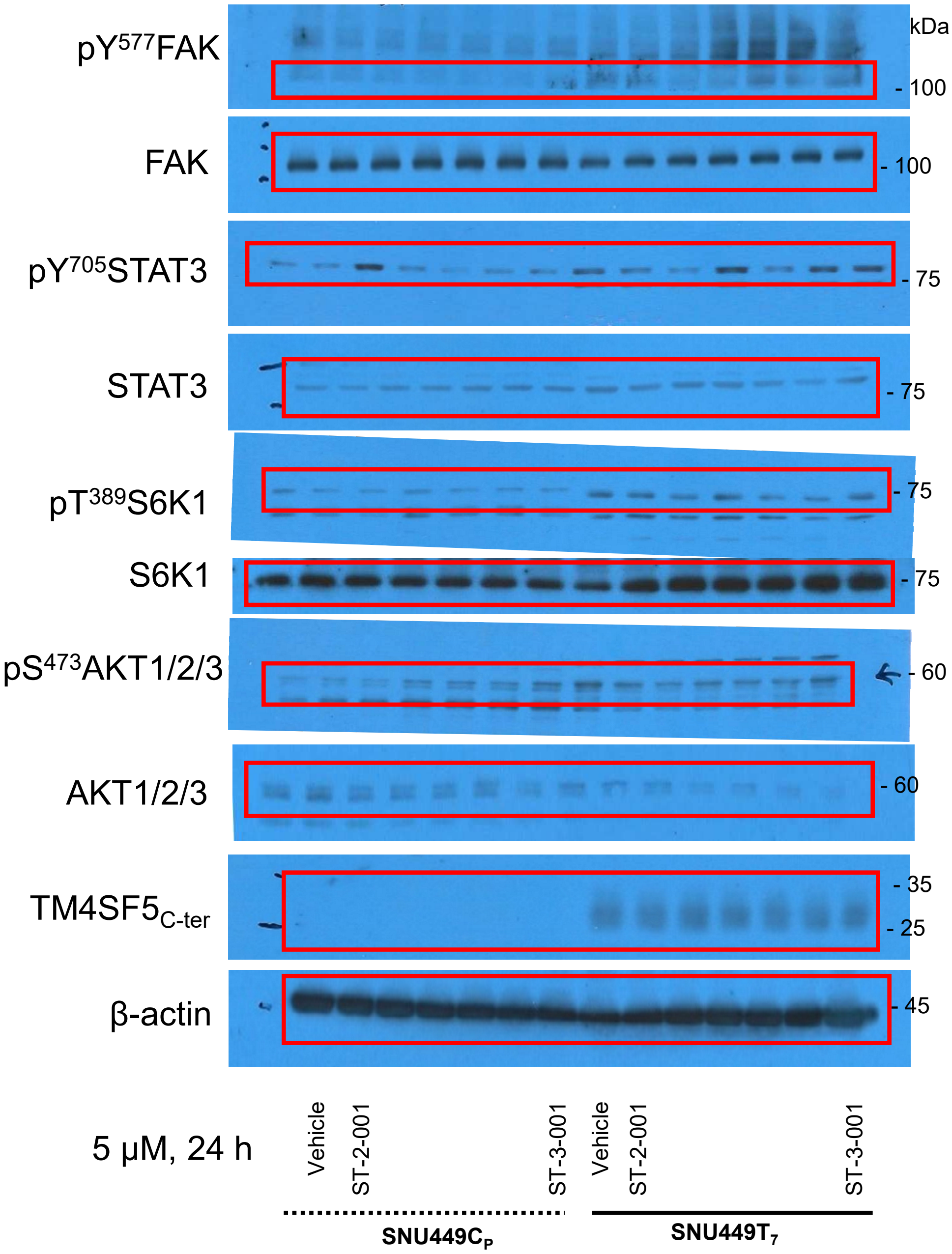

Figure S8c

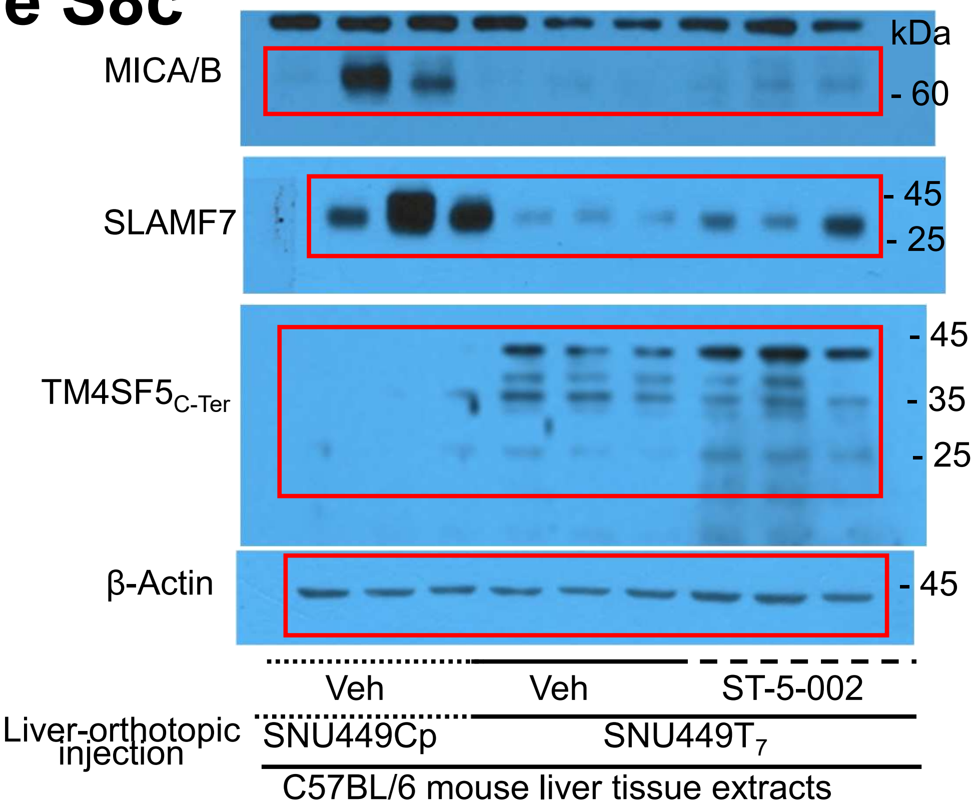

Supplement: Supplementary file 3 — Supplementary Uncut immunoblot gel images [file 41392_2024_2106_MOESM3_ESM.pdf]
